# Development of a Soy Protein Hydrolysate with an Antihypertensive Effect

**DOI:** 10.3390/ijms20061496

**Published:** 2019-03-25

**Authors:** Eric Banan-Mwine Daliri, Fred Kwame Ofosu, Ramachandran Chelliah, Mi Houn Park, Jong-Hak Kim, Deog-Hwan Oh

**Affiliations:** 1 Department of Food Science and Biotechnology, Kangwon National University, Chuncheon 200-701, Korea; ericdaliri@yahoo.com (E.B.-M.D.); fkofosu17@gmail.com (F.K.O.); ramachandran865@gmail.com (R.C.); 2 Erom Company Limited, R&D Center, 111, Toegye Nonggong-ro, Chuncheon-si, Gangwon-do 24427, Korea; mhpark@yahoo.com (M.H.P.); jhkim@yahoo.com (J.-H.K.)

**Keywords:** antihypertensive peptides, functional food, food-derived, fermentation

## Abstract

In this study, we combined enzymatic hydrolysis and lactic acid fermentation to generate an antihypertensive product. Soybean protein isolates were first hydrolyzed by Prozyme and subsequently fermented with *Lactobacillus rhamnosus* EBD1. After fermentation, the in vitro angiotensin-converting enzyme (ACE) inhibitory activity of the product (P-SPI) increased from 60.8 ± 2.0% to 88.24 ± 3.2%, while captopril (a positive control) had an inhibitory activity of 94.20 ± 5.4%. Mass spectrometry revealed the presence of three potent and abundant ACE inhibitory peptides, PPNNNPASPSFSSSS, GPKALPII, and IIRCTGC in P-SPI. Hydrolyzing P-SPI with gastrointestinal proteases did not significantly affect its ACE inhibitory ability. Also, oral administration of P-SPI (200 mg/kg body weight) to spontaneous hypertensive rats (SHRs) for 6 weeks significantly lowered systolic blood pressure (−19 ± 4 mm Hg, *p* < 0.05) and controlled body weight gain relative to control SHRs that were fed with physiological saline. Overall, P-SPI could be used as an antihypertensive functional food.

## 1. Introduction

High blood pressure (hypertension) is a chronic degenerative disease and the leading risk factor for chronic kidney disease and cardiovascular diseases [1]. Uncontrolled hypertension can result in chronic damage to the vascular system, myocardial strokes, and even death [2]. For this reason, several pharmacological and nonpharmacological strategies aimed at reducing the incidence of this disease have been implemented. Over the years, several functional foods have been developed from different food proteins to be used as nonpharmacological treatments of high blood pressure [3]. More so, many food-derived bioactive peptides have demonstrated antihypertensive effects. Such peptides commonly inhibit angiotensin-converting enzyme (ACE) activity and/ or reduce renin activity [4,5]. Soybean proteins are among the most common plant substrates used for food-derived antihypertensive peptide development [6,7]. Soy proteins constitute about 35–40% of the total dry weight of the bean and the major storage proteins are glycinin (11S globulin) and β-conglycinin (7S globulin). These storage proteins account for about 65–85% of the total soy proteins [8]. Recent studies have shown that consumption of fermented soybean meal can reduce the risk of cardiovascular disease mortality, cardiovascular disease, stroke, and coronary heart disease risk [9,10]. However, to release bioactive peptides from parent proteins, enzyme treatment and fermentation are the two most common methods used. While enzyme hydrolysis saves time and enhances scalability and predictability of peptides, fermentation (though a relatively slow process) is a cheaper strategy to generate various bioactive peptides with diverse activities. Combining the two methods could enhance the number and kinds of peptides released from the parent protein. A number of lactic acid bacteria such as *Pediococcus pentosaceus* and *Lactobacillus casei* have been used to ferment soy proteins to release antihypertensive peptides of high potency [2,11,12]. In an earlier study, we found that *Lactobacillus rhamnosus* EBD1 isolated from Korean fermented soybean (doenjang) had strong proteolytic activity and could be helpful in generating bioactive peptides.

Therefore, to develop a soybean product with a strong antihypertensive effect, we first hydrolyzed soy protein isolates (SPI) with Prozyme and subsequently fermented the hydrolysate with *Lactobacillus rhamnosus* EBD1 to obtain a product we named P-SPI. This combined strategy enhanced the degree of hydrolysis of the proteins. The long term effect of P-SPI consumption on the systolic blood pressure of spontaneous hypertensive rats (SHR) was studied.

## 2. Results

### 2.1. The Extent of Hydrolysis

**Figure 1:**
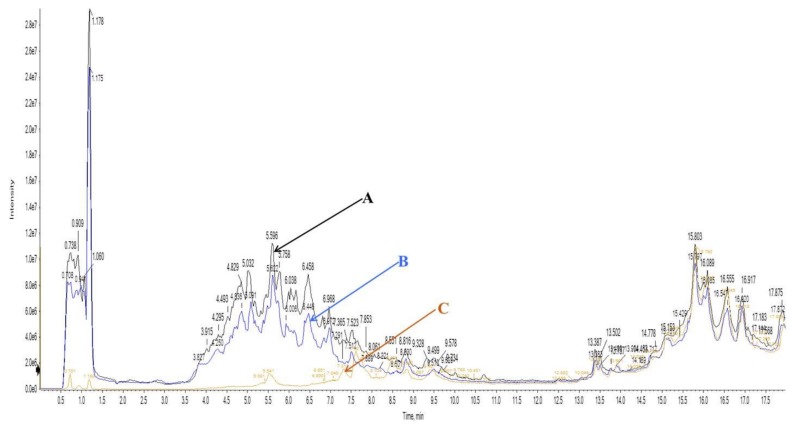
RP-HPLC chromatograms of various stages of sample preparation: (**A**) represents the chromatogram of raw soy protein isolates (SPI), (**B**) represents the Prozyme treated SPI and (**C**) represents the product (P-SPI).

*L. rhamnosus* EBD1 was able to grow on SPI as a sole nitrogen source and both Prozyme and *L. rhamnosus* digested SPI to various extents. Analysis of hydrolysates by RP-HPLC showed that approximately 15% of the substrate was hydrolyzed by Prozyme after treatment for 1 h. However, subsequent fermentation of the hydrolysate with *L. rhamnosus* EBD1 for 48 h resulted in hydrolysis of approximately 55% of the initial SPI concentration (Figure 1).

### 2.2. ACE-Inhibitory Ability of Hydrolysates and Fermentates

Both the enzyme hydrolyzed and fermented hydrolysates inhibited ACE to various extents (Table 1). As shown, fermentation of the enzyme hydrolyzed samples improved the ACE inhibitory activity from 60.8% to 88.24%.

### 2.3. ACE Inhibitory Peptides from P-SPI

**Figure 2:**
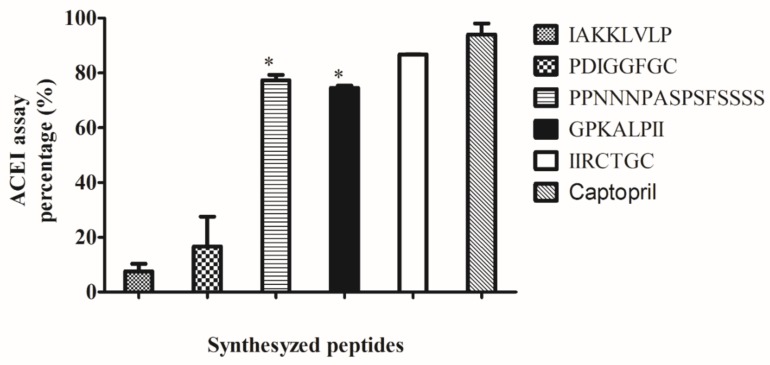
The ACE inhibitory ability of selected peptides from P-SPI. Bars represent the means of three replicates (*n* = 3) ± SD, *p < 0.05.

All the peptides identified in the peptide profile are displayed in Supplementary Table S1. Since 3008 peptides were identified and could not be individually synthesized/eluted and tested in vitro for ACE inhibitory activity, the peptide sequences were screened using an in silico platform (http://crdd.osdd.net/raghava/ahtpin/index.php) developed by Kumar et al. [13] to predict potential ACE inhibitory peptides. Although many potential ACE inhibitory peptides were identified, peptides IAKKLVLP, PDIGGFGC, PPNNNPASPSFSSSS, GPKALPII and IIRCTGC were most abundant. The peptides were synthesized and their ACE inhibitory activities were confirmed in vitro. Among the peptides tested, IIRCTGC showed the strongest inhibitory activity of 83 ± 0.9%, followed by PPNNNPASPSFSSS and GPKALPII, which were not significantly different in their inhibitory abilities (*p* > 0.05) (Figure 2). Meanwhile, peptide PPNNNPASPSFSSSS showed an inhibitory activity of 18 ± 7%, while IAKKLVLP displayed the least inhibitory activity of 10 ± 3%. Captopril (the positive control) showed the strongest inhibitory activity of 94 ± 4%.

### 2.4. The Effects of Gastrointestinal Enzymes on ACE Inhibitory Activity of P-SPI

**Figure 3:**
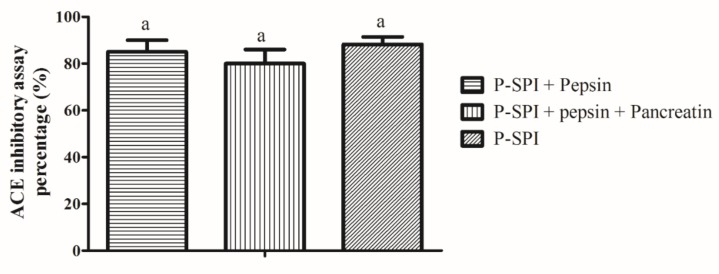
The effects of gastrointestinal enzymes on ACE inhibitory activity. Bars represent means of three replicates (*n* = 3) ± SD, ^a^ *p* < 0.05.

When P-SPI was subjected to pepsin digestion, its ACE inhibitory activity was not significantly altered. Also, subsequent treatment of the peptides with pancreatin did not affect the ACE inhibitory ability (*p* > 0.05) as shown in Figure 3.

### 2.5. Blood Pressure Reducing Effects of P-SPI

**Figure 4:**
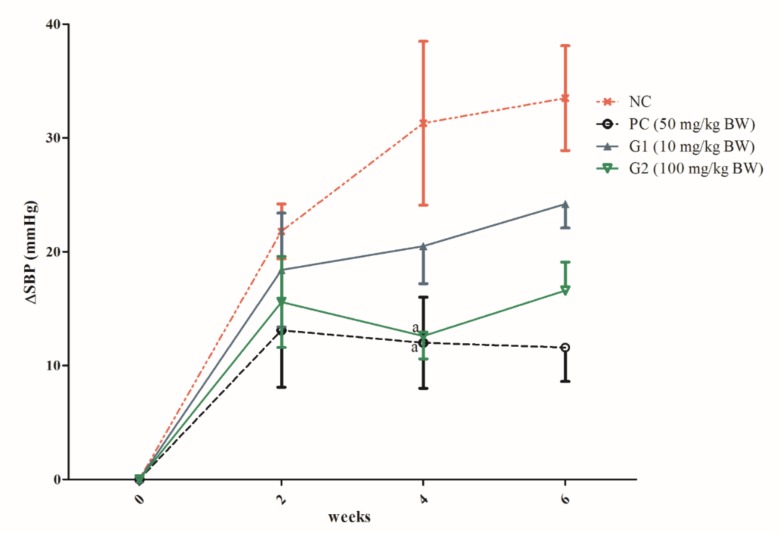
Systolic blood pressure changes from the baseline are expressed in absolute values (mmHg) and data are mean ± SEM from 5 determinations. Data points with the same alphabets are not significantly different (^a^ *p* > 0.05) using one-way ANOVA followed by Duncan tests. NC: Negative control, PC: Positive control, G1: Group 1, G2: Group 2.

The mean SBP measured for all the SHRs from the four experimental groups prior to treatment (zero time) was 179 ± 5.6 mm Hg (*n* = 20). Oral administration of P-SPI at a dose of 100 mg/kg resulted in a significant reduction in SBP when compared to the lack of SBP reduction by physiological saline. The decrease in SBP was maximal at the 4th week of oral administration (−19 ± 4 mm Hg). By contrast, oral administration of 10 mg/kg P-SPI induced a slight decrease in SBP which was maximum at the 4th-week post-administration (−11.2 ± 2 mm Hg). However, all the P-SPI doses reduced SBP significantly when compared to the negative control group and maintained lower blood pressures throughout the feeding period. 50 mg/kg captopril administration resulted in an SBP reduction of 22 mm Hg (Figure 4).

### 2.6. Effects of P-SPI Consumption on Body Weight Gain

**Figure 5:**
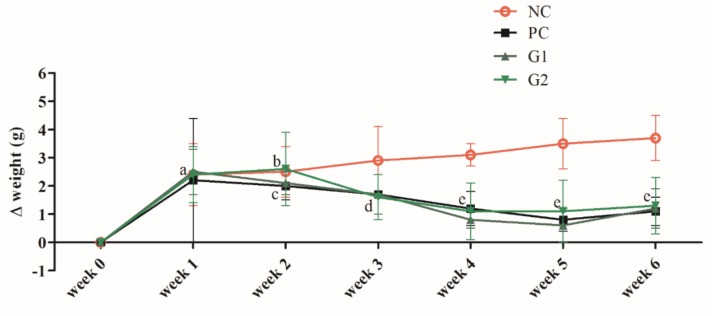
Time course of weight changes (Δ weight) after oral administration of physiological saline, 50 mg/kg, and 100 mg/kg body weight of P-SPI. Each data point represents mean ± SEM from 5 determinations, and data points with the different alphabets are significantly different (*p* < 0.05) using one-way ANOVA followed by Duncan tests. NC: Negative control, PC: Positive control, G1: Group 1, G2: Group 2.

P-SPI (10 mg/kg and 100 mg/kg) and captopril administration significantly reduced weight gain from the 2nd week to the 4th week of feeding (*p* < 0.05) relative to SHRs that were given physiological saline. The weights of the rats in these groups were however maintained after the 4th week up to the 6th week of feeding (Figure 5).

### 2.7. Effects of P-SPI Consumption on Feed Intake

**Figure 6:**
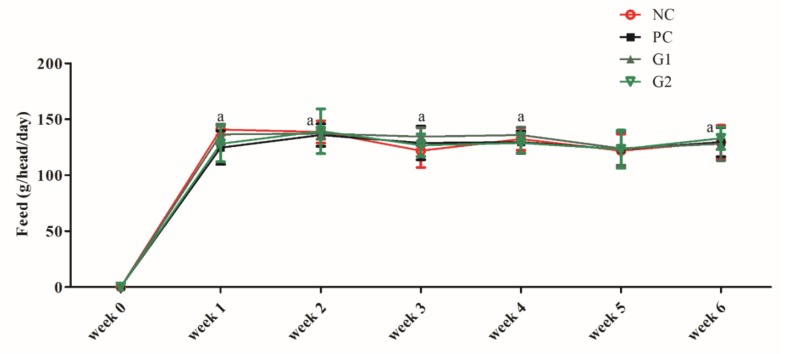
Each data point represents mean ± SEM from 5 determinations, and data points with the different alphabets are significantly different (*p* < 0.05) using one-way ANOVA followed by Duncan tests. NC: Negative control, PC: Positive control, G1: Group 1, G2: Group 2.

Generally, neither the administration of P-SPI nor captopril affected the quantity of feed intake of SHRs (Figure 6). The quantity of feed consumed by the rats in the test and control groups was not significantly different throughout the course of study (*p* > 0.05).

## 3. Discussion

To generate bioactive peptides from soy proteins, proteolytic enzymes or microorganisms would be required to release the peptides from the parent proteins. The use of fermented soybean or enzyme hydrolyzed soy proteins for reducing high blood pressure is well recognized. However, very few studies (if any) have exploited the combined effects of proteolytic enzymes and fermentation for developing antihypertensive foods.

### 3.1. Hydrolysis and Fermentation of Soy Proteins

Hydrolysis with proteases and subsequent fermentation reduces the time needed for efficient substrate hydrolysis when only fermentation is employed. Also, this strategy allows the generation of new antihypertensive peptide sequences that would not have been generated if only a single method was applied. The combined processing method in this work enhanced hydrolysis of the soy protein compared to the enzyme treatment alone. *Lactobacillus rhamnosus* is known for its well-developed protein degradation machineries with which it hydrolyzes proteins. Peptides generated by the cell-envelope proteinase hydrolysis are transported into the bacterial cell for further hydrolysis by peptidases so as to meet its nitrogen requirements [14,15]. The cell number increases from 2 × 10^8^ cells to 10^9^ cells after 48 h incubation.

### 3.2. Effects of P-ISP on ACE Activity

The product obtained (P-SPI) displayed strong ACE inhibitory ability of 88.24 ± 3.2% (IC_50_ = 0.592 mg/mL) compared to raw SPI. Using LC-ESI-TOF-MS/MS, we identified 3008 peptides in P-SPI samples which were generated by the processing method. In silico screening of peptides using the AHTpin software available at http://crdd.osdd.net/raghava/ahtpin/index.php revealed many potential ACE inhibitory peptides, among which IAKKLVLP, PDIGGFGC, PPNNNPASPSFSSSS, GPKALPII, and IIRCTGC were most abundant. Since these 5 peptides satisfied some of the common structural features described for many food-derived ACE inhibitory peptides [16,17,18], they were synthesized and their inhibitory activities were tested in vitro. As seen in Figure 2, only PPNNNPASPSFSSSS, GPKALPII, and IIRCTGC were strong ACE inhibitors, while IAKKLVLP and PDIGGFGC were weak inhibitors. Earlier studies about structural-activity relationships between peptides and ACE inhibition indicated that peptides whose C-terminal tripeptides are hydrophobic show a stronger binding ability to ACE [16], and this could account for why GPKALPII showed good ACE inhibition. Also, peptides with branched-chain aliphatic amino acids or hydrophobic amino acid at the N-terminal have been shown to be good competitive inhibitors of ACE. These criteria make IIRCTGC and PPNNNPASPSFSSSS good ACE inhibitory candidates [17,18].

### 3.3. Effect of Gastrointestinal Enzymes on P-ISP Activity

In the gut, ingested peptides encounter gastrointestinal enzymes and may be hydrolyzed. This may either result in loss of activity or generation of other potent peptides. Treatment of P-SPI with gastrointestinal enzymes in vitro, however, did not significantly affect ACE inhibitory activity (*p* > 0.05), indicating that the ACE inhibitory peptides were either resistant to gastrointestinal enzyme digestion or retained their activity even after digestion.

### 3.4. The Effect of P-ISP Consumption on Systolic Blood Pressure

Recent studies have indicated that systolic blood pressure is a better factor for predicting cardiovascular disease than diastolic blood pressure [19,20]; hence, reducing SBP reduces the risk of CVD. Results from this study showed a clear reduction in SBP when SHR were fed with P-SPI (10 mg/kg BW and 100 mg/kg BW) for six weeks. Nevertheless, the effect of P-SPI was less pronounced than the effect of captopril (a standard antihypertensive drug). However, this study was aimed at developing a functional food that could prevent or reduce high blood pressure rather than curing the condition. Compared to synthetic antihypertensive drugs, food-derived antihypertensive peptides have been reported to have no side effects, have higher tissue affinities, and maybe more slowly cleared from tissues [21].

### 3.5. The Effect of P-ISP Consumption on Feed Satiety and Body Weight Gain

Many studies have found a strong association between obesity and hypertension in humans [22,23,24]. This is because an increase in body weight seems to be followed by an increase in blood pressure. However, whether obesity precedes hypertension or hypertension leads to obesity still remains unclear. Yet, due to the association between these two conditions, we studied the effect of P-SPI consumption on SHR body weight. Relative to the untreated group, P-SPI reduced SHR body weight gain significantly from the second week to the fourth week of feeding. SHR body weight was, however, maintained (*p* > 0.05), though they were continuously fed with P-SPI from the 4th–6th weeks. A similar observation was made among SHRs administered with captopril.

Some studies have shown that certain bioactive peptides decrease appetite and lead to reduced food intake, resulting in reduced weight gain [25,26,27]. For this reason, we checked whether P-SPI consumption affected feed intake relative to control groups. It was observed that P-SPI consumption did not have any significant effect on the quantity of feed consumed by the rats (Figure 6). It is therefore possible that the reduction in weight gain might have been caused by other reasons apart from increased satiety.

The relationship between long term soy protein hydrolysate consumption and blood pressure has been discussed in many previous reports. For instance, Yang et al. [28] reported that pepsin hydrolyzed soy peptides reduced SBP (up to −35 mmHg) after SHR were fed for twelve weeks. Rhyu [29] also observed an SBP reduction of about −13 mmHg when SHR were fed with fermented soybean paste for seven weeks. These studies, however, used low molecular weight peptides mixed with some other foods. We believe our study is a better representation of how food could be processed and directly consumed as a functional food for reducing high blood pressure. Our results are similar to Wu et al. [30], who recorded an SBP reduction of about −20 mm Hg when SHRs were fed with 100 mg/kg BW of soy proteins hydrolyzed with Alcalase. It is, however, obvious that the different enzymes used for SPI hydrolysis result in different peptides with different potencies for ACE inhibition. For this reason, different treatments would result in different abilities to lowering blood pressure. Meanwhile, any small reduction in high blood pressure could beneficially reduce the risk of cardiovascular diseases [31].

In conclusion, our data demonstrates that Prozyme hydrolysis followed by *Lactobacillus rhamnosus* EBD1 fermentation enhanced bioactive peptide generation and improved ACE inhibition. Consumption of P-SPI could therefore be helpful in reducing high blood pressure in humans. Meanwhile, studies about the mechanism(s) by which P-SPI reduces blood pressure are warranted.

## 4. Materials and Methods

### 4.1. Chemicals and Cultures

Soybean protein isolates (Pro-Fam^®^) were obtained from Archer Daniels Midland Company (ADM, Decatur, Illinois, USA). Hip-His-Leu, ACE (from rabbit lung), Pepsin (from porcine gastric mucosa), and Pancreatin (from porcine pancreas) were obtained from Sigma-Aldrich (Yongin, Korea). Prozyme 2000P was obtained from Bison Corporation, Gyunggi-Do, Korea. *Lactobacillus rhamnosus* EBD1 was obtained from the Department of Food Science and Biotechnology (Chuncheon-si, Gangwon-do, Korea) and used for this study because it showed strong proteolytic ability in our earlier study [32]. The bacteria stock culture was maintained at −80 °C in de Man, Rogosa, and Sharpe (MRS) broth (Difco, Hongcheon, Korea), containing 20% glycerol (*v*/*v*). The culture was streaked on MRS agar and cultured at 37 °C for 24 h. A single colony was then transferred into MRS broth at 37 °C and harvested at the exponential phase of growth.

### 4.2. Preparation of Protein Hydrolysates and Fermentation

SPI was hydrolyzed with Prozyme according to the enzyme manufacturer’s instructions. Briefly, 20% (*w*/*v*) of SPI in distilled water was prepared, and the pH was adjusted to 7. The protein was digested by 3% Prozyme at 55 °C for 1 h. The sample was then autoclaved at 121 °C to stop the enzyme activity and to sterilize the sample. *Lactobacillus rhamnosus* EBD1 (2 × 10^8^ cfu/mL) in the starter culture was inoculated into a 500 mL Erlenmeyer flask containing the hydrolyzed SPI. Cultivation was carried out at 37 °C with 150 rpm of agitation. After 48 h incubation, the fermented sample (P-SPI) was freeze-dried with a TFD5505 table top freeze dryer (ilshinBioBase Co. Ltd., Dongducheon-si, Korea) and stored at −20 °C for further analysis.

### 4.3. Determination of Extent of Proteolysis

The degree of proteolysis of the SPI samples (raw SPI and P-SPI) were analyzed as reported earlier [33] with slight modifications. Briefly, SPI hydrolysates were analyzed by reversed-phase high-performance liquid chromatography (RP-HPLC) using a Waters system (Waters Corporation, Milford, MA, USA) equipped with a 1525 Binary HPLC pump, a 2996 Photodiode Array Detector, and a 717 plus Autosampler. An aliquot (90 μL, 10 mg/mL) of the sample was applied to a Symmetry^®^ C_18_ 5 μm, 4.6 × 150 mm column (Waters, Milford, MA, USA). The column was developed at a flow rate of 1 mL/min at 40 °C. Elution was performed with a linear gradient of solvent B (acetonitrile with 1% TFA) in solvent A (water with 1% TFA) from 0–80% in 60 min. Detection of peptides and proteins was carried out at 214 nm. The extent of proteolysis was calculated by expressing the chromatographic peak areas of either enzyme treated alone or enzyme treated and fermented ISP hydrolysates as a percentage of that of raw SPI.

The degree of hydrolysis after enzyme treatment was calculated as:

(1) $$\mathrm{Degree}\;\mathrm{of}\;\mathrm{hydrolysis}\;=\;\frac{100\%\;\times \;\left(\mathrm{Peak}\;\mathrm{area}\;\mathrm{of}\;A-\mathrm{Peak}\;\mathrm{area}\;\mathrm{of}\;B\right)}{\left(\mathrm{Peak}\;\mathrm{area}\;\mathrm{of}\;A\right)}$$

The degree of hydrolysis after enzyme treatment and fermentation was calculated as:

(2) $$\mathrm{Degree}\;\mathrm{of}\;\mathrm{hydrolysis}\;=\;\frac{100\%\;\times \;\left(\mathrm{Peak}\;\mathrm{area}\;\mathrm{of}\;A-\mathrm{Peak}\;\mathrm{area}\;\mathrm{of}\;C\right)}{\left(\mathrm{Peak}\;\mathrm{area}\;\mathrm{of}\;\mathrm{raw}\;A\right)}$$

where A represents the chromatogram of raw SPI, B represents the Prozyme treated SPI, and C represents P-SPI.

### 4.4. In-Vitro Assay for ACE Inhibitory Activity

ACE inhibitory activity was determined by the procedure described by Cushman & Cheung, [34]. Briefly, 20 μL of ACE inhibitor solution with 50 μL of 5mM HHL in 100mM sodium borate buffer (pH 8.3) containing 0.3M NaCl was incubated at 37 °C for 5 min. To initiate the reaction, 10 μL of 0.1 U/mL ACE solution was added, and the mixture was incubated at 37 °C for 30 min. The reaction was terminated by adding 100 µL of 1M HCl, and the reaction mixture was mixed with 1 mL ethyl acetate. The mixture was vortexed for 60 s and centrifuged at 2000× *g* for 5 min. The ethyl acetate layer (0.8 mL) was transferred to a 1.5 mL Eppendorf tube and evaporated in a water bath. The hippuric acid (HA) in the tube was dissolved with distilled water (0.8 mL). The amount of HA formed was measured at 228 nm using a biospectrometer (Eppendorf Biospectrometer^®^ fluorescence, Eppendorf Korea Ltd. Korea). The amount of HA liberated from Hip-His-Leu under this reaction conditions without an inhibitor was used as a control. The extent of inhibition was calculated as

ACE inhibition = 100 % × [(B − A)/B]

where A is the optical density in the presence of ACE and ACE inhibitory component and B is the optical density without ACE inhibitory component.

For the determination of IC_50_, series of dilutions containing 5000 μg/mL, 500 μg/mL, 50 μg/mL, 5 μg/mL, 0.5 μg/mL, and 0.05 μg/mL of P-SPI samples were prepared. The amount of peptides required to suppress 50% ACE activity was calculated from the regression curves observed for each fraction.

### 4.5. Identification of Peptides by Mass Spectrometry

Liquid chromatography-electrospray ionization-quantitative time-of-flight tandem mass spectrometry experiments (LC-ESI-TOF-MS/MS) were carried out at the National Instrumentation Center for Environmental Management of Seoul National University in Korea, according to an earlier method [35]. Analysis was done using high-performance liquid chromatography (UltiMate 3000 Series system, DIONEX Technologies, Sunnyvale, CA, USA), an integrated system comprising an auto-switching nano pump, an autosampler (TempoTM nano LC system; MDS SCIEX, Seoul, Korea), and a hybrid quadrupole-time-of-flight (TOF) mass spectrometer (QStar Elite; Applied Biosystems, USA) fitted with a fused silica emitter tip (New Objective, Woburn, MA, USA). To ionize the samples, nano-electrospray ionization was used. 1.5 g of the P-SPI was dissolved in 50 mL of double distilled water. Fractions (1.5 μL) of the sample were injected into the LC-nano ESI-MS/MS system. The sample was trapped on a ZORBAX 300SB-C18 trap column (300-μm i.d × 5 mm, 5-μm particle size, 100 pore size, Agilent Technologies, Santa Clara, California, USA, part number 5065-9913) and washed for 6 min with gradient with 98% solvent A [water/acetonitrile (98:2, *v*/*v*), 0.1% formic acid] and 2% solvent B [Water/acetonitrile (2:98, *v*/*v*), 0.1% formic acid] at a flow rate of 5 μL/min. The peptides were separated on a ZORBAX 300SB-C18 capillary column (75-μm i. d × 150 mm, 3.5 μm particle size, 100 pore size, part number 5065-9911) at a flow rate of 300 nL/min with a gradient at 2%–35% solvent B over 30 min, then from 35%–90% over 10 min, followed by 90% solvent B for 5 min, and finally 5% solvent B for 15 min. Electrospray was performed at an ion spray voltage of 2000 eV through a coated silica tip (FS360-20-10- N20-C12, PicoTip emitter, New Objective). The peptides were analyzed automatically using Analyst QS 2.0 software (Applied Biosystems, Seoul, Korea). The range of m/z values was 200–2000. Peptides of interest were ordered at >95% purity from GL Biochem (Shanghai, China) Limited (Shanghai, China).

### 4.6. Effects of Gastrointestinal Enzymes on P-SPI (In Vitro)

A two-stage simulated gastrointestinal digestion was carried out on P-SPI similar to an earlier report [2]. Pepsin (0.2 mg) was added to 10 mL of 1 mg/mL P-SPI solutions and adjusted to pH 2.0 using 1 M HCl. The samples were incubated at 37 °C. After 120 min, the pH was raised to 7.5 by adding 1 M NaOH. Pancreatin (0.2 mg) was added and the samples were further incubated at 37 °C for 180 min. The reaction was stopped by heating at 80 °C for 10 min in a water bath, followed by cooling at room temperature. The samples were analyzed for their ACE inhibitory abilities.

### 4.7. Long-Term Effect of P-SPI Consumption on SHR Blood Pressure

All animal experimental procedures were in accordance with the ethical procedures and scientific care by Kangwon National University-Institutional Animal Care and Use Committee (approval no. KW-151127-1, 13 August 2018). Twenty male SHRs weighing 250–300 g were used (Charles River Laboratories, Barcelona, Spain). The rats were divided randomly into four groups. Rats were housed in temperature-controlled rooms (23 °C) with 12 h light/dark cycles and consumed tap water and standard diets ad libitum. Experimental procedures were conducted in accordance with the Kangwon National University animal ethics committee guidelines. Indirect measurement of systolic blood pressure (SBP) in awake restrained rats was carried out by the non-invasive tail-cuff method using computer-assisted non-invasive blood pressure equipment (NIBP 76-0173 unit with LE5160R cuff & transducer, Sang Chung Commercial Co., Ltd., Kangnam-Ku, Korea). The rats were kept at 37 °C for 15 min to make the pulsations of the tail artery detectable. By gastric intubation, each group of rats was either administered with 10 mg of P-SPI per kg body weight (BW), 100 mg of P-SPI per kg BW, captopril (50 mg/kg BW), or 750 μL physiological saline once daily. SBP was measured before peptide intake (week 0), at the 2nd, 4th and 6th weeks after intake. Each value of SBP was obtained by averaging five successful measurements without disturbance of the signal. Changes in SBP were calculated as the absolute difference (in mmHg) with respect to the basal values of measurements obtained just before starting the treatments.

### 4.8. Effect of P-SPI Consumption on Feed Consumption and Weight Gain

To assess the amount of feed consumed by the rats, the mass of feed supplied to the rats each morning was recorded, and the remaining feed in the feeding trough was weighed the next morning. The difference in mass was recorded as the amount of feed consumed by the rats.

For weight gain assessment, the weight of each rat in each group was recorded once every week (from week 0–6) and the changes in body weight were noted as weight gain or loss.

### 4.9. Statistical Analysis

Baseline systolic blood pressure was defined as the mean of the values measured in the first run-in period. Blood pressures, weight, and the amount of feed consumed were presented as the mean value ± standard deviations (SD) for all SHR in each group. The outcomes for each week between groups were analyzed with one-way ANOVA followed by Duncan tests. Differences were considered significant when *p* < 0.05. All statistical analysis was done using GraphPad Prism version 5.01 (GraphPad Software, Inc, La Jolla, CA, USA).

## Figures and Tables

**Table 1:** Angiotensin-converting enzyme (ACE) inhibitory activity of processed SPI

Sample	Inhibitory Activity (%)	IC_50_ (mg/mL)
Raw SPI	10.21 ± 4.0	n.d
Prozyme treated SPI	60.8 ± 2.0	0.980
P-SPI	88.24 ± 3.2	0.592
Captopril	94.20 ± 5.4	0.005

Data shows mean ± SD (*n* = 3). Values represent the means of three replicates ± S.D. n.d: Not determined.

## Supplementary Materials

Supplementary materials can be found at https://www.mdpi.com/1422-0067/20/6/1496/s1. Table S1: LC-ESI-TOF-MS/MS analysis of P-SPI-derived peptides.
