# Supplementary material for: Development of a Soy Protein Hydrolysate with an Antihypertensive Effect
Source: Int J Mol Sci. 2019 Mar 25;20(6):1496. doi: 10.3390/ijms20061496 (PMC6470933; doi:10.3390/ijms20061496)
Supplement: Supplementary file 1 [file ijms-20-01496-s001.pdf]

Table S1: LC-ESI-TOF-MS/MS analysis of P-SPI-derived peptides

Peptide Sequence	Prec m/z	Theor MW	Prec z
AAAQSQSSCTNVLVSLSPCLNYITGNSSTPSSGC	837.5884	3346.512	0
AAEFEPSS	419.2506	836.3552	2
AAEKTDMGYISLNGFLSQW	533.4506	2130.009	0
AAGKEMGLH	457.34	912.4487	0
AAGLLKLEGIHEGRK	796.5148	1590.921	2
AAHVTNEGLDAAGHAIGTAWAVF	760.3182	2278.113	3
AAKAFVRVWK	588.3919	1174.698	2
AAKAFVRVWK	588.3919	1174.698	2
AAKAFVRVWK	588.3919	1174.698	2
AAKYIIHNVELKSVK	571.7062	1711.999	3
AALALAVRAGSVL	606.3935	1210.74	2
AALFVIACHTG	551.893	1101.564	2
AAPVAFIAAVVIVV	670.461	1338.828	2
AAQVVNGCN	438.2853	874.3967	2
AAVKFRWGVRIPAEFKGNNAF	595.3783	2377.281	0
ACGKLRALN	473.3423	944.5226	0
ACGMQ	255.065	508.1774	2
ACPPPG	271.0599	540.2366	2
ACRICFNDSKVLLTQLCPNQKEAIGWYDNCMLRYSNRSIF	786.3422	4712.258	6
ACSAFRNAQTENLF	786.3457	1570.72	2
ACTTNNPDERIGIREAVRRLKAAR	678.4767	2709.457	0
AEFAHGNVQQSMEMLNCN	506.4749	2021.839	0
AEKKTGVTVTGQTTGGVKSV	975.0653	1948.048	2
AEMMKVFDDIKLCNCSPDIVTWNTLLAVFGQN	904.6917	3614.728	0
AFAHLETQFKF	669.9554	1337.677	2
AFCSTSSM	425.1347	848.3044	0
AFFTDFAD	467.2058	932.3916	0
AFGLQKN	389.2641	776.4181	0
AFGVFVYE	311.1828	930.4487	0
AFLEDA	333.1674	664.3068	2
AFLETIVNNR	588.8909	1175.63	2
AFNVAGS	333.1669	664.3181	2
AFQVLRGSKCGR	441.2959	1320.709	0
AFTGVHGYGFALV	669.9554	1337.677	2
AFTGVHGYGFALV	669.9554	1337.677	2
AFTVGSKQ	419.2505	836.4392	2
AFVASVSA	751.5147	750.3912	0
AGASYVSSALVTTVHHVTRTH	549.375	2193.129	0
AGCNAME	348.0698	694.2415	2
AGGVVILLLAVAAILRTL	588.4114	1762.144	3
AGISIGVVTGLLLLAF	772.4872	1542.939	2
AGKYDMGALCMHAHSEMLEALINDALDKGA	794.5619	3174.461	0

AGMSTKKYETK	415.2111	1242.628	0
AGNKVRP	371.2185	740.4293	0
AGPAGH	255.0648	508.2394	2
AGPAHG	255.065	508.2394	2
AGTPKKNEI	957.505	956.5291	0
AGYYDTEHTKNVRDWKEVF	786.6649	2357.108	3
AHARICSIIRK	634.45	1266.734	0
AHCPMCR	409.1619	816.3193	0
AHGFLVSDAACCPSGCNPNQKPCNNR	676.4974	2702.157	0
AHKANNNTQFE	425.2114	1272.585	0
AHLKSKKS	449.8242	897.5396	2
AHNAARSEVGVQNLAWDDTVAAFAQNYANQ	808.5825	3230.513	0
AHNGGLVPL	439.2902	876.4818	2
AHNIGETLQEK	620.4099	1238.626	2
AHNNGSKCKSSG	397.1817	1188.531	0
AHNNKRH	438.7846	875.4474	2
AHNRITN	413.2563	824.4253	2
AHQKLSELGFAE	665.4094	1328.673	2
AHQMTSCR	467.2123	932.3956	0
AHRKVDKPRFVSAF	553.3487	1656.921	3
AHRKVGKRR	554.3708	1106.69	2
AHSSVHK	383.166	764.3929	0
AHVADFGLAKFLQDTGTSECMSSIAGSYGYIAPEY	925.6397	3698.691	0
AHVELSSM	437.1919	872.4062	0
AIFGKQN	389.2645	776.4181	0
AIFYLKSF	494.8228	987.543	2
AIHPLMKKQ	533.3454	1064.617	2
AIKKAS	617.4013	616.3908	0
AIKKAS	617.403	616.3908	0
AIKKAS	617.4004	616.3908	0
AIKKAS	617.4045	616.3908	0
AIKKSNKLPQAVVLK	546.3979	1636.04	0
AINHDLH	819.4487	818.4035	0
AISSFARASK	519.4002	1036.567	0
AIVADDSDLAKIL	672.4331	1342.734	2
AIVHALL	736.5279	735.4643	0
AIYREVVADLKQAGA	802.5187	1602.873	2
AKAKTNFPTHA	593.3996	1184.63	0
AKANKARK	443.8049	885.5508	2
AKAYVVVNDSCYHQLISH	683.3071	2046.983	3
AKDLKLHTKQLRRTL	607.7579	1820.111	3
AKEQRKPYVVVFV	782.4858	1562.882	2
AKFDVKIT	461.3176	920.5331	2
AKHKVEFVDG	565.4086	1128.593	2
AKHKVEFVDG	565.4103	1128.593	2

AKISKFIPALN	601.4376	1200.723	2
AKITPIVILSC	579.425	1156.689	2
AKKRAKRQKK	621.4088	1240.82	2
AKLDKCRKLIK	658.4669	1314.817	2
AKLPRLRGVVLHSGI	808.5797	1615.005	0
AKNTVASKLGFG	596.908	1191.661	2
AKSQPSNSIPDRTLMIEEVGAEIEKQNMQES	865.6199	3458.666	0
AKSVHGYVIRKEMAG	549.3742	1644.877	0
AKTSRQLRK	544.4125	1086.662	2
AKVGLKAGGFFVLKE	782.5586	1562.919	0
ALALLHV	736.5279	735.4643	0
ALENNNPGFISIDCGSEAAYSHTETGIWF	786.5972	3142.398	0
ALHKEPDTEICASMLDSLNEC	580.5119	2318.023	0
ALIKATTSNYN	598.3967	1194.625	2
ALKKALKN	443.3014	884.5807	0
ALKKHGNN	441.2637	880.4879	2
ALKKHGNN	441.264	880.4879	2
ALLVMVKKKRGGGR	756.9897	1511.945	2
ALLVTPPAKVIQGV	703.5068	1404.871	2
ALQKRARVKK	599.3931	1196.783	0
ALQKRARVKK	599.392	1196.783	0
ALQKRARVKK	599.3928	1196.783	0
ALQVTSSSVRLVSSN	774.5001	1546.832	2
ALRNFAFA	455.311	908.4868	0
ALRVKPAHPIVGAVAPIF	619.4369	1855.12	0
ALRYQQCLEIISS	762.3227	1522.781	2
ALSSAKGERKLKTKPKQKATK	575.4101	2297.391	0
ALVVGEAEKRTISVIIK	609.4113	1825.104	3
ALVVLKENLVLTLF	786.5034	1570.97	2
ANGLLVLYITI	595.3799	1188.712	0
ANGNKVTVGVIQW	693.4857	1384.746	2
ANIKAIRAAS	507.8671	1013.598	2
ANKKKSQKGN	551.893	1101.626	2
ANLHITSSH	490.3563	978.4883	2
ANLVLF	676.4975	675.3956	0
ANNQPAAASVVTYDQNQYYSDKSGSYVANN	810.5562	3238.444	0
ANPPIFHR	476.3061	950.5086	0
ANPQTEGDLPKVT	685.4332	1368.689	0
ANSLIDMYA	499.2474	996.4586	0
ANTPLSHISPVVGR	724.4502	1446.794	2
ANVVWLHLGVNSG	683.4274	1364.72	0
APGGNGATG	351.1752	700.314	0
APIFN	281.1794	560.2958	2
APILGEARLLSVGLF	786.5034	1570.908	2
APKTGKTKPHKAKGEKK	925.5664	1849.09	2

APLISH	637.4276	636.3595	0
APLLPNK	752.5151	751.4592	0
APPLPV	593.4027	592.3585	0
APPLPV	593.4014	592.3585	0
APPLPV	593.402	592.3585	0
APQDPPIW	462.2543	922.4549	2
APQKN	279.2315	556.2969	2
APQNK	279.2306	556.2969	2
APRNKAKP	441.3132	880.5243	2
APSHGEASSSNNNTTMYGFQMQTS	637.4761	2546.044	0
APVCYVAG	390.2314	778.3683	2
APVPNFFVPMVQQGQQGQRPGGRRPGAVQQ	808.5799	3230.663	0
AQAALGVVFKLVEI	729.4785	1456.865	2
AQATKSRN	438.2853	874.4621	2
AQFFTSMLCHMKFGLFIFF	772.3473	2314.114	3
AQFRKKIATLS	631.9361	1261.751	2
AQHIIRKLF	563.37	1124.682	2
AQLLSISLVALGS	636.4635	1270.75	2
AQLQELDGEDARLADYFDVIAGTSTGGIVTAM	832.6314	3326.598	0
ARFPPNQI	471.8381	941.5083	2
ARILSEICIIVN	672.4331	1342.764	2
ARPAH	551.3547	550.2976	0
ARRAAFAF	455.3114	908.498	0
ARSLLSASRASH	628.4073	1254.679	2
ARVKPPKN	455.333	908.5556	2
ASAAVLWNK	959.5198	958.5236	0
ASAHNMS	367.1713	732.2861	0
ASGAASAPLRN	507.8672	1013.525	2
ASIAVGLRGTLLHVAIVQAAL	519.1124	2072.247	0
ASKKKNSAKSVR	652.4084	1302.773	0
ASNCD	255.0648	508.1588	2
ASNLQCGTFNTNLGCAEFGSIEQRGMFQRGTNSATTVSMGNRHV	1173.802	4691.148	0
ASQHLATLTGHQGP	709.4482	1416.711	0
ASQNVKHP	440.8135	879.4562	2
ASQTYKSNIELIENSQKS	681.3088	2040.985	3
ASRSKSKDRKANKDAQK	959.52	1917.05	0
ASVYKSLHNQIAPGQKWNPLNFVLGCSFL	808.5789	3230.67	0
ATAKTNTTIYQDVF	786.8363	1571.783	2
ATGTTVRSA	432.2802	862.4509	0
ATLSVVLNALAID	650.4203	1298.745	2
ATRRIVRPPKK	441.3724	1320.847	0
ATTTTNGGAMEELLSQLQSHVQ	772.6533	2315.106	3
ATVANCKQL	457.2691	912.5029	0
AVAARKKFRFKK	725.519	1448.909	2
AVADLRHKNLV	618.3949	1234.715	2

AVDEDNKGNYAKAF	771.3305	1540.716	2
AVFPNIYTAMANR	734.3217	1466.734	2
AVGKHIFLC	494.3195	986.5372	2
AVGYGIGFSH	504.3285	1006.487	2
AVIGSFGYIAPEYVQ	538.6729	1612.814	3
AVIHRDM	421.3191	840.4276	0
AVISIPAFSH	521.3857	1040.565	2
AVKKLNNLLFQQ	708.4875	1414.83	0
AVMILLAV	415.2706	828.5143	0
AVPLFH	683.4311	682.3802	0
AVQVELQPNRN	634.4502	1266.668	0
AVRKVASSFH	551.391	1100.609	2
AVTLVLVSYAMGDSAQDKQRCAESLAG	696.5413	2782.363	0
AYCFTFAF	485.1756	968.4102	2
AYINKAKNSS	548.3516	1094.572	2
AYKVLLEMQIGG	441.2802	1320.711	3
AYNCAIY	409.1609	816.3476	0
AYNNKAGST	463.3009	924.4301	0
AYYKDFNIEGCAVPGPANCASNPSNWWEGA	808.5771	3230.386	0
CAEAVLPDSCENTDSITSQDLF	786.6649	2357.004	3
CAITVRKSALQKLI	772.4888	1542.928	2
CAKSFAPFPNGC	311.145	1240.537	0
CAKSFAPFPNGC	311.1464	1240.537	0
CCGFGRKTNCT	397.1795	1188.484	0
CCGFGRKTNCT	397.1811	1188.484	0
CCHSQTEPIELNGQARFREKNPATAVAS	765.1545	3056.456	0
CCKNKAFKCQT	425.2106	1272.578	0
CDNARTQ	404.2053	806.3341	0
CFCALVPP	425.2108	848.3925	0
CFNENAYMAACVAVNTCF	493.385	1969.783	0
CFNIGLPSGK	518.3412	1034.522	2
CFRNTSFG	311.1452	930.4018	0
CFVSSTFCLIKAGAV	773.3507	1544.773	2
CGKRN	289.1524	576.2802	2
CGNSGGSKQ	419.2496	836.3447	2
CGSGLSGETISENGHHWIGLDISASMLNVAVER	860.6633	3438.63	0
CIHVCEVC	453.1677	904.3605	0
CIRRTP	373.2708	744.4065	0
CKQVLEERN	559.8896	1117.555	2
CKRLFERRGFRIT	561.3934	1680.936	0
CLKKKGH	813.4637	812.4691	0
CLSLDVVFAIAFLV	378.212	1508.831	0
CMCGQ	271.0604	540.1495	2
CMVWFVVDYGACG	367.1705	1464.588	0
CNTDFFPEALQNSEVTSSSNCCYEEN	733.5419	2930.132	0

CPELVDRERCTDMAFSWTMQSCQVLY	786.5955	3142.333	0
CSSDLRIRRGLR	716.4594	1430.789	2
CTCTCSCP	409.161	816.2275	0
CTGYVISSKSG	551.3537	1100.517	0
CTKWSWPSCCATKCS	423.3606	1689.677	0
CTYINNPH	481.2371	960.4124	0
CTYINNPH	481.2378	960.4124	0
CTYINNPH	481.2376	960.4124	0
CTYINNPH	481.2368	960.4124	0
CYNCGLVFCHSCSS	381.2957	1521.551	0
DAKPAVTDVSKPSALAKK	913.5139	1825.031	0
DAMPTIKITHE	628.4073	1254.628	2
DCFGVQYFGYFSDDIGYSVQFGGPGGSCGN	786.5969	3142.275	0
DCFIVCVNCDSPKCEGDCKPCINCLSS	965.7225	2894.155	0
DDVGLVR	387.1916	772.4079	0
DEEEQPQCVETDKGCQRQS	736.3023	2205.908	3
DEEEQPQCVETDKGCQRQS	736.3023	2205.908	3
DEERVMFRSVGLRMKAYLPLGIRGVF	760.582	3038.631	0
DEERVMFRSVGLRMKAYLPLGIRGVF	760.5834	3038.631	0
DEERVMFRSVGLRMKAYLPLGIRGVF	760.583	3038.631	0
DEGPPS	301.1388	600.2391	2
DEKGRISTYKVEF	786.3473	1570.799	2
DFGAEYVIESSGVF	760.3215	1518.688	2
DFIKTKMGNRSDMGFSTPQGPRGSDDVSSN	808.5799	3230.472	0
DFLGKDG	376.2608	750.3548	2
DFQREVLDGTQDKIEDDHEENQRKR	776.1432	3100.445	4
DFRSLTLVSTDLMLRSAREF	786.3421	2356.221	3
DFRVSVKKIKWN	507.3302	1518.867	3
DGACSNAVVEAELDESVKSKEKKKKKNKTTNKKK	941.5072	3762.031	0
DGDFEEEEVWAVF	786.3393	1570.646	2
DGGDVSADPGGGSG	383.1669	1146.443	0
DGPPNSF	367.171	732.3079	0
DGRYAGSHSSYNGEVEEGMIPEEEGMI	736.5307	2942.233	0
DGTLLAPS	387.1799	772.3967	2
DHGAVPKLVKLL	645.4893	1288.787	0
DHMGVPLL	441.2967	880.4477	0
DHNHPLVVSPREARQTM	497.4146	1985.985	0
DHNNSF	367.1709	732.2827	0
DHNQAKESCNQESTPMPESS	555.4563	2217.89	0
DHNRRFKGYGHVEF	441.2958	1760.85	0
DHNSSSS	367.1697	732.2675	0
DHNSSSS	367.17	732.2675	0
DHNSSSS	367.1705	732.2675	0
DHNSSSS	367.1712	732.2675	0
DHRNCSS	409.1614	816.3058	0

DIKLEKILLCDDFCPKISDFRLAKLRKKEDMATMSRRKGT	943.345	4711.516	5
DIKLEKILLCDDFCPKISDFRLAKLRKKEDMATMSRRKGT	943.3455	4711.516	5
DIKLEKILLCDDFCPKISDFRLAKLRKKEDMATMSRRKGT	943.3455	4711.516	5
DIKLEKILLCDDFCPKISDFRLAKLRKKEDMATMSRRKGT	943.3455	4711.516	5
DKAQSTRPKGHC	443.2172	1326.646	0
DKHEKDESKDKNDC	423.3781	1689.726	0
DLHPFTVP	463.3022	924.4705	2
DLHSFDTELLPEMYGVHNEYLT	656.4903	2622.195	0
DLKPFFNSSHFS	357.2605	1424.673	0
DLPAVM	323.1442	644.3204	0
DLSTQTSGTLTRDWSPMSIC	550.4986	2197.998	0
DLTPSPAKPVRN	647.9415	1293.704	2
DNDGVSEMIVAVSYFFDHEYYDNQEHR	820.5967	3278.389	0
DNHVMETCFKLHGHPDWHLKGKTTPNAKVDAI	910.6662	3638.787	0
DNMNLGWKETNCSDDVLSNTSPINTPSH	773.1239	3088.35	4
DNNNKSTK	460.8006	919.4359	2
DPKLGVPLLLTIM	705.511	1408.836	0
DPNWK	659.2844	658.3075	0
DPSIGL	301.1421	600.3119	2
DQIMKRLVSAARR	772.5242	1542.878	2
DQIRQCS	425.2113	848.381	0
DQYYCLSKECLNQTS	449.3573	1793.76	0
DRAHAALPPGYRF	735.9954	1469.753	2
DRHRRRRSS	409.1624	1224.666	0
DSAHIPTKLFL	621.3947	1240.682	0
DSDPCNDHQPSVDVEVEKQ	535.9001	2139.902	4
DSRYQLVAKGK	632.9174	1263.694	2
DSWGSGKRKDKVTQEDNSGSGGWGANR	720.5243	2878.334	0
DTFLLGDLEDSDEAPDDGEGSFD	615.4917	2457.982	0
DTGHCEEGLQKAEESISIKRSFEAYFL	772.5836	3086.466	0
DTPEAT	317.2043	632.2653	2
DVAIYGGC	399.1407	796.3425	0
DVFWDLKGAKF	663.3461	1324.682	2
DVGHN	271.0604	540.2292	2
DVTSKPMPAVHQ	663.3461	1324.645	2
DWMKTAMGVSKQDLMET	493.3842	1969.895	0
DYENSYPWCKNES	409.3636	1633.636	0
DYMGQFG	409.1614	816.3112	0
DYNRMARSS	367.1712	1098.488	0
DYPPSHRPTG	376.2588	1125.52	0
EADDPGALAAVGCLRAISTILESVSSLPQLF	786.5973	3142.622	0
EAFPWSCNSSFMYTMENHNSVLGIREYA	821.592	3282.421	0
EAFRFDYEKMEEEFKIFV	786.3352	2356.109	3
EANTHFKYFKDNPDSNNPAYNTKKGIYD	1097.801	3290.527	0
EASSSAQELHCFSDFSFQEIKEATSNFNPSKKIG	941.7003	3762.747	0

EAYVIGHH	463.3274	924.4454	0
EAYVIGHH	463.3277	924.4454	0
ECQSEDVDFESEAKKQVCG	533.4521	2129.888	0
EDCESI	348.0698	694.248	2
EDVTILLS	445.2313	888.4804	0
EECCM	614.178	613.1546	0
EEEEVRCLRNCKHIFH	681.3104	2040.962	3
EEIDYELSKCQVEAESQPIF	786.3352	2356.078	3
EEIDYELSKCQVEAESQPIF	786.3353	2356.078	3
EELGNGKTRFYPVGPITQKRSIEETDESDKCL	910.6626	3638.789	0
EELSKKSG	439.2888	876.4553	2
EERPGKN	415.2109	828.409	2
EFGEKRQGT	351.1746	1050.509	0
EFKGKTLRCSLSETKHRLFIGNVPK	963.6014	2887.585	0
EGEEGSGGAAERLEEISSENSGPTRSRSEDDFEGGEAEPEDDDDAHGDN	857.219	5137.044	6
EGGVCSCIGSALCQEQSTPDDDVLEEEN	732.5478	2926.179	0
EGHFLSSWTMGGNP	760.3225	1518.656	2
EGISYQVTRI	389.2653	1164.614	0
EGKNWVKILVLTGS	772.4888	1542.877	2
EGKSLAEAEGLFFMETSALDSTNVKMAF	760.5823	3038.425	0
EHHEKK	404.2054	806.4035	0
EHKLFLVGLQK	656.4381	1310.771	2
EHKQQQWLRFNQSTK	653.4212	1956.992	0
EHNLQILF	507.3259	1012.534	0
EHVDDGAGKPHPKIHTIFSTECTTYFDW	808.5795	3230.477	0
EIAGEKAGIFKHRIPAF	628.7576	1883.042	3
EICIGVACGLHYLHAGAKRSIF	786.6649	2357.214	3
EIEKGRLIR	557.412	1112.667	2
EIQVLRKISTTLA	736.5004	1470.877	2
EIRQKME	467.2038	932.475	0
EISELQKEVTDLKNLVDDLNNKQGG	700.5533	2798.43	0
EISFVPTT	447.3458	892.4542	2
EIVYSGRVS	505.3469	1008.524	0
EKDNIDMDNVCSNLEYCVPR	786.3359	2356.013	3
EKGKKLTKQ	530.4028	1058.645	0
EKKK	532.3831	531.3381	0
EKKK	532.3836	531.3381	0
EKKKDTMHGKGNF	760.3215	1518.761	2
EKLKVFVVPH	598.3967	1194.713	2
EKSSDKMVPPEDEEWKKLEETILSHVVLIHN	919.6795	3674.85	0
EKVCAVLKGRAEELGKLYN	707.4871	2119.146	0
EKVKTF	376.313	750.4276	2
ELDYLSGSPIKDEVIQREEAIRKEYKELDTVW	963.7028	3850.963	0
ELEANEANGHNIDNEEYS	683.3071	2046.84	3
ELITGQRALEFGKAAN	573.3596	1716.916	3

ELKAIAPHS	483.3757	964.5342	2
ELKAIAPHS	483.3757	964.5342	2
ELKGRRVGKV	571.4282	1140.709	2
ELKLGEGGFGGVYK	727.4555	1452.761	0
ELKMLVALILS	615.4544	1228.747	2
ELKVRHKKPH	636.4652	1270.762	2
ELLELIDKKLS	650.9235	1299.765	2
ELNYSEMSSSNTYNSCSSGC	541.4408	2161.787	0
ELPEPKQP	469.3595	936.4916	2
ELQGTYIERFREFLEDLCN	594.5289	2374.127	0
ELRKLKQKF	595.379	1188.734	0
ELRKQGYN	504.3367	1006.52	0
ELTAPWTGVQIKVPVKFI	507.327	2025.166	0
ELTKPISLVY	581.9031	1161.665	2
ELVLKSAAKAFQAQ	752.4873	1502.846	2
EMPKIEKWVNVF	760.3215	1518.791	2
EPTPGKKLR	513.3854	1024.603	2
EQETAVIELQ	387.179	1158.577	0
EQGLVMDPPGGDSDDRKRKRDMLDLSDGDSD	859.5977	3434.532	0
ERMGECAAERWQK	797.2888	1592.719	2
ERNKGVEDQAYTNDMDCYYYSTSELPCKKHPSS	965.7186	3858.645	0
ERPCLLYSSSSK	685.4283	1368.671	2
ERSGLSEGIPSLPEYLADYCSL	600.4638	2398.136	0
ESISFGRF	471.8381	941.4607	2
ESNSVSHTIVPEQDFEQLPALPDAVACQFSALEK	925.6392	3698.777	0
ESSSNQSIN	483.1742	964.4098	2
ETLPNN	344.224	686.3235	2
EVDRKGKVRSLPGHEEFPDVGGKIVGSF	760.5843	3038.594	0
EVIDKIKEY	568.8928	1135.613	2
EVPVNGSF	425.2123	848.3916	0
EVVINPPKSLRPPGRPRKKRV	808.5792	2422.476	0
EYGKQRRIQKCG	367.1692	1464.762	0
EYGKQRRIQKCG	367.1703	1464.762	0
EYGKQRRIQKCG	367.1711	1464.762	0
EYHVCRNH	529.197	1056.456	2
EYNKSENSE	367.1707	1098.447	0
EYPYNNFLHH	667.2846	1332.589	2
EYRRKNNFHSD	367.1708	1464.686	0
EYVKQCRSS	367.1718	1098.513	0
EYVKQCRSS	367.1726	1098.513	0
EYVKQCRSS	367.1709	1098.513	0
EYVKQCRSS	367.1713	1098.513	0
EYVKQCRSS	367.1714	1098.513	0
EYVKQCRSS	367.1715	1098.513	0
EYVKQCRSS	367.1703	1098.513	0

EYVKQCRSS	367.1698	1098.513	0
EYVKQCRSS	367.1701	1098.513	0
EYVP	507.3534	506.2377	0
EYVPNGS	383.1667	764.3341	0
EYVPNGS	383.1655	764.3341	0
FAKQK	311.1871	620.3646	2
FANGINPKKPVQN	713.9822	1425.773	2
FATIVNKYKAAMIEDVPRIFEAVFQCTLEMITK	955.7556	3818.981	0
FCFGLILP	455.3096	908.483	0
FDIDKNLGKKCNA	367.1705	1464.74	0
FDNWWVGPQTEAKQNAGSGAS	563.2134	2249.014	4
FEALLQEFKTDYNQTHF	533.4501	2130.006	0
FECLHIASSGSYNHTDSC	493.3853	1969.794	0
FEDMISWMQEGGQVGIFDATNSSKQRRN	808.5803	3230.487	0
FEFESCSFDAVLCTVSVQYLQQPEKVF	786.5979	3142.467	0
FEFESCSFDAVLCTVSVQYLQQPEKVF	786.5954	3142.467	0
FELDDKEKQ	576.3842	1150.551	2
FENRCL	391.285	780.3589	2
FEPSVGVGVVFLVST	513.0022	1535.824	3
FESPEAKKQ	532.3572	1062.535	2
FEVVVSAGQK	532.3572	1062.571	2
FFGRMTFWW	639.4049	1276.585	0
FFKCLHKLKNAVKLH	609.4097	1825.055	3
FFNGDWNTGGSCDNT	409.3621	1633.61	0
FFNIRLSKG	541.3468	1080.608	2
FGAFGSGGGS	422.2924	842.3559	2
FGCFLVMDPNNPTSINSALRYWGCTIQAG	794.5611	3174.472	0
FGFQCNCS	453.1674	904.3207	0
FGGTAA	262.1445	522.2438	2
FGVVLLELVTGRNPNKAG	628.7576	1883.063	3
FHFLRAIGTA	378.2114	1131.619	0
FIGGLANLSQEQKYDRKQADMNLLMLF	786.598	3142.595	0
FIIAVVF	808.5798	807.4894	0
FIMGGFLN	449.8241	897.4418	2
FIPLIGGIFSRKKGS	810.5525	1618.956	0
FIPPYKQRVS	617.9318	1233.687	2
FIVGKGGR	417.292	832.4919	2
FIVYGVNLTFVN	693.459	1384.739	2
FKASADDVTQTPITCLNCSKCEYPCQQQPSPPLPYV	997.6994	3986.82	0
FKDLLSFFVEVKCAPSVTAANRVVNSLCSS	808.5796	3230.647	0
FKGKRVVFLKQLP	520.6934	1558.971	3
FKGLVDKRLKLRN	794.0364	1585.978	2
FKGMSSRN	463.8406	925.444	2
FKISRIKIKIQDSRF	627.1062	1878.12	3
FKKVGHKK	486.3776	970.6076	0

FKPLRKLL	507.8672	1013.675	2
FKRTRVLTA	546.3722	1090.661	2
FKRWK	764.5504	763.4493	0
FLASPFR	419.3158	836.4545	2
FLCPKEVRSGAKRI	802.5187	1602.903	2
FLEELMSLRRETWDTNPCSQENQLF	772.5826	3086.411	0
FLFLLASQDLIMVASRCFCCGKPLNPGGS	772.5833	3086.522	0
FLGGGFVKHMQENEFLQEVF	786.0136	2355.136	3
FLHGAMSF	455.2216	908.4214	0
FLIQMERCDN	634.8705	1267.569	2
FLKKCKNKAGKSNTM	425.2858	1696.912	0
FLKLGVKTRG	559.8896	1117.697	2
FLLLDTSLNN	575.3804	1148.608	2
FLLLLTAYSSA	599.8706	1197.665	2
FLNAANFS	442.3187	882.4236	2
FLNLEKLV	488.3311	974.5801	2
FLNLISA	777.5279	776.4432	0
FLPPNQRGTVTGRLLVLDGGK	560.4131	2237.264	0
FLPWQSWK	546.3722	1090.56	2
FLSLRKGT	461.3621	920.5444	2
FLSLSPLGCLPALRALNPEANKDGCFEAASA	794.5609	3174.584	0
FLSPCRGVSLTVPSGQLGPLDAT	772.3407	2314.199	3
FLSTPPIPSEILAKG	523.997	1568.881	3
FLVILSKKVKKKVLTTRTG	720.5245	2158.393	0
FMEM	279.1586	556.2025	2
FMEM	279.1577	556.2025	2
FMFFLFMY	573.219	1144.513	2
FNANPSPNLFSLLF	527.6783	1579.804	3
FNDK	262.1445	522.2438	2
FNEMRNKGC	367.1712	1098.459	0
FNENEIVDGIKAENLIGKGGSGN	594.5274	2374.177	0
FPGSEHPFVDRPLEPYVMPPRSGREMRPRARR	955.7519	3818.948	0
FQDTDLLQFVPNQAC	435.358	1737.803	0
FQELVEIGAKK	421.3194	1260.708	0
FQGKVAIVTAS	560.8615	1119.629	2
FQVTRLQPN	551.893	1101.593	2
FRARYPSAK	548.3518	1094.599	2
FRHATHI	441.3113	880.4668	2
FRILREFL	547.3422	1092.644	2
FRKRTRRR	588.3919	1174.727	2
FRNIFSKKGMK	678.4761	1354.754	0
FRRQRN	438.787	875.4838	2
FRYDETC	467.1001	932.3698	0
FRYDETC	467.2112	932.3698	0
FSCATADC	409.1611	816.2782	0

FSCFSS	339.1398	676.2527	0
FSFTYGDM	323.1442	966.3793	0
FSGPCKPGLVFQVDGTLMA	492.4608	1965.969	0
FSGVLHAGQRIFVL	772.5004	1542.867	2
FSKRLIHH	519.3438	1036.593	2
FSLAMRL	419.2496	836.4578	2
FSLEVILTLFAFKCM	441.2971	1760.925	0
FSPAILLRLF	588.8909	1175.707	2
FSPATRMKNNAPNFS	425.211	1696.799	0
FSPVILKNEM	393.2957	1176.621	0
FSQNMKNHDLNSS	381.1861	1520.668	0
FSQNMKNHDLNSS	381.1863	1520.668	0
FSQNMKNHDLNSS	381.1867	1520.668	0
FSQNMKNHDLNSS	381.1855	1520.668	0
FSQNMKNHDLNSS	381.1862	1520.668	0
FSSFSA	323.1455	644.2806	0
FSSLR	609.3946	608.3282	0
FSVLHPINKL	584.3805	1166.681	2
FTGGPGGGSG	397.18	792.3402	0
FTNGRVMPRSC	423.1969	1266.596	0
FTSNKENNST	381.1867	1140.505	0
FVAGKN	635.4109	634.3439	0
FVANNLNK	460.2895	918.4923	2
FWGFFPLFQAIF	760.3227	1518.77	2
FYALFFDNDNIF	763.3296	1524.693	2
FYMIKNGEAILPKKGYFIYGIAVLTKLDPAWGRKYK	1048.82	4191.311	0
FYNHVVA	425.2138	848.4181	0
FYRRAQAYIETGDYL	467.3149	1864.911	0
GAAGLLRR	813.53	812.498	0
GAFRPGVLTALVGVTG	758.0082	1513.862	2
GAMLVYDMTKRQSFDHMVKWLEELRGHADQNIVIMLI	1097.799	4387.21	0
GAPAKKKKLG	997.6907	996.6444	0
GARHWIETLQ	404.2066	1209.626	0
GASSSAGLEEKKEVK	760.3227	1518.789	2
GDAALNCLKIHWFGMSFFVLWTS	661.4838	2642.282	0
GDFMVPSQGDSSSATEEAPTSTTTSVTAVA	733.5429	2930.298	0
GDLTGIKKHYSRKHGEKK	521.3215	2081.149	0
GDNRAMSENEVEI	732.3097	1462.636	2
GDSSSVTEEAPTSTTTTVTAVTENPPGGGERRRKYRG	963.7054	3850.868	0
GEEVAECIDPAKKKELDGLRAEMPVALAEGDR	860.6635	3438.712	0
GEKVKKLAEQYA	455.3121	1362.751	0
GEKVKKLAEQYA	455.3097	1362.751	0
GEKVLVQFEDFANHNA	455.3086	1816.874	0
GELPHLK	397.2369	792.4494	2
GELPHLK	397.2369	792.4494	2

GELPHLK	397.2355	792.4494	2
GFGCKSVNAC	493.3046	984.4157	0
GFIDITIS	433.2914	864.4593	0
GFPAVI	302.1956	602.3428	2
GFSFSSQ	380.2463	758.3235	2
GGAPRGSFEN	496.3288	990.4519	2
GGDQIEGETSRIVGTYGYMAPEYAFDGLF	786.5981	3142.423	0
GGGFIIVEGFH	378.2117	1131.571	0
GGGGAHGG	285.0752	568.2354	2
GGGGGHQ	285.0752	568.2354	2
GGGGGYIGSVMKDGEGRTFNLSGRNGRKFL	772.5814	3086.547	0
GGGKKGKGRGGGGRGVRGV	566.3714	1695.972	3
GGGSCNKT	362.2159	722.3018	0
GGIVVLAIVLIFLYVV	563.3897	1687.069	3
GGKLVLHA	794.5627	793.481	0
GGKRVRP	769.5065	768.4719	0
GGLGGPGFGASLFPGLGGNNGMGGGGLNGLFGAGF	760.583	3038.435	0
GGMHAATGILTARGG	685.4283	1368.693	2
GGTHVAIAFEVP	599.3867	1196.619	2
GGWCS	255.0648	508.174	2
GHGGGKGNTDKDNESG	383.167	1528.65	0
GHGMLGHG	383.1657	764.3388	0
GHIPREIGKLSN	441.279	1320.715	3
GHIVTFKGC	481.2348	960.4851	0
GHKCGSG	323.1452	644.27	2
GHKCGSG	323.1448	644.27	0
GHKCGSG	323.1462	644.27	2
GHKRPIKKVKHSKS	815.5584	1628.995	2
GHKTIFG	380.2463	758.4075	2
GHMDNWSS	467.1009	932.3447	2
GHNKRRII	497.3201	992.5992	2
GHNKRRII	497.3198	992.5992	2
GHNNIGFS	423.1955	844.3828	0
GHNNLTASNKHPSLN	802.3647	1602.786	2
GHNNNPI	383.1645	764.3566	0
GHNNTGVF	423.1965	844.3828	0
GHQHNFEKNLLKCGW	453.3901	1809.873	0
GHRCCWGS	453.1673	904.3432	0
GHRDLILG	440.8135	879.4927	2
GHRKQINEYALD	481.9996	1442.727	3
GHSSNNSDYNPRSS	385.1812	1536.619	0
GHSTSYN	383.1652	764.309	0
GHSTSYN	383.1667	764.309	0
GHSTSYN	383.1665	764.309	0
GHSTSYN	383.1677	764.309	0

GHSTSYN	383.1641	764.309	0
GHSTSYN	383.1654	764.309	0
GHSVVTLFRF	581.9034	1161.63	2
GHTGPISRCCFSASGN	797.3348	1592.682	2
GHVSGPQLWDPHDFDFGKNFG	786.3356	2356.066	3
GIGKTYIWRTLAS	733.5206	1464.809	2
GIHINLKGFA	535.3581	1068.608	0
GIIGNYQQRNQ	430.906	1289.648	3
GIKPGK	599.3929	598.3802	0
GINSNITKRLAIRLPPS	925.5664	1849.09	2
GIPIFH	683.4321	682.3802	0
GIPQGFFMLGSYCQPNNKPLF	786.6715	2357.134	3
GIRNSTSSCKRI	441.279	1320.693	3
GIVLIHVKN	496.8749	991.6179	2
GKALLGRPLR	540.9018	1079.693	2
GKATILAIGKAF	595.4003	1188.723	2
GKEAIVAGFYH	596.3879	1190.608	2
GKERWPNSHFVNGEHALKFPLF	653.4203	2609.329	0
GKGKSTR	733.5419	732.4243	0
GKIIISAVSLILVVGV	527.6784	1580.028	3
GKKAPLRQR	527.4025	1052.657	2
GKKEKRH	441.8127	881.5195	2
GKKITDDCPNSDEIAYRVYVTSNDAILS	772.5836	3086.487	0
GKLNVHLIAH	551.3546	1100.646	0
GKLVKPKYYKTIKCSITLDPK	808.5812	2422.402	0
GKNKVS	632.4734	631.3653	0
GKPLPKF	786.5981	785.48	0
GKQSQKHLPIV	617.9318	1233.719	2
GKRIVHV	808.5808	807.5079	0
GKRIVHV	808.5801	807.5079	0
GKRLATY	808.5796	807.4603	0
GKRSTFL	808.581	807.4603	0
GKTAASIAASH	507.3645	1012.53	2
GLEILAIP	413.2662	824.5007	0
GLFTYLGKRTRIF	786.4819	1570.898	2
GLGIHHSGVEVHGVEYAFGAHDYPTSGVF	760.5824	3038.431	0
GLGIHHSGVEVHGVEYAFGAHDYPTSGVF	760.5839	3038.431	0
GLGIHHSGVEVHGVEYAFGAHDYPTSGVF	760.5826	3038.431	0
GLLGLGGGFILGPLFI	772.5028	1542.917	2
GLLGLGGGFILGPLFI	772.4838	1542.917	2
GLLRMNSS	439.2769	876.4487	2
GLNPISLLIDCAKCVASGSI	494.3182	1973.033	4
GLNQCGSF	413.2634	824.3487	0
GLPLIIQFI	507.3273	1012.632	0
GLQEKGVALTKVPKVKF	461.3222	1841.114	0

GLRGCQKW	474.3152	946.4807	2
GLRKILKKFD	609.395	1216.766	0
GLSFNLIKLKSFENMVAV	507.3253	2025.097	0
GLVIKKKGPVF	593.4408	1184.765	2
GLYKLSKL	461.3294	920.5695	2
GMPLPKDPSKKGNLRIKFN	535.9075	2139.199	4
GMRFASQAILSG	413.2646	1236.629	0
GNFLKK	706.5034	705.4174	0
GNLKTCSC	413.2641	824.3521	0
GNPGGYGSG	383.1653	764.309	0
GNPGGYGSG	383.1665	764.309	0
GNTELNFS	441.2074	880.3926	0
GPCPAA	258.1097	514.2209	2
GPDHLAALAPLSIGRTRMESAAVGALWGCGHDAGQVIFGLIFL	1097.803	4387.272	0
GPHEKRN	419.2496	836.4253	2
GPHLHHI	405.7843	809.4296	2
GPHLSAGAK	419.2509	836.4504	2
GPIIGGPFTL	494.3195	986.5437	2
GPILSH	623.4113	622.3439	0
GPILSH	623.4113	622.3439	0
**GPKALPII**[d]	808.579	807.5219	0
**GPKALPII**[d]	808.5803	807.5219	0
**GPKALPII**[d]	808.5765	807.5219	0
**GPKALPII**[d]	808.5791	807.5219	0
**GPKALPII**[d]	808.5786	807.5219	0
GPKGILINVGR	562.3683	1122.687	2
GPKGLPIL	794.5639	793.5062	0
GPKKLPLIGNM	584.3805	1166.685	2
GPKKPIF	786.5978	785.48	0
GPKKPIF	786.5953	785.48	0
GPKLQKAIDQ	549.415	1096.624	2
GPKNVHVVPAASN	645.4635	1288.689	2
GPLGIEEGPILWAS	360.3619	1437.75	0
GPQPIRRTG	491.3723	980.5516	2
GPQRRKRR	527.4025	1052.643	2
GPRIH	579.3846	578.3289	0
GPRNKYL	424.273	846.4712	2
GPRNKYL	424.273	846.4712	2
GPSEMVFRCQACNRTSELL	535.9001	2139.986	4
GQDQDNQEFPFNFSTQKISIFNKISTTMIATGYKIECCMAF	786.172	4711.174	6
GQGGSIPSEETVKLLDMIKSGGVNLGLS	697.4454	2785.453	0
GQGHNIKVTSFKVG	491.3334	1470.794	0
GQPPLAIL	808.5806	807.4854	0
GQPPLAIL	808.579	807.4854	0
GQPPLAIL	808.5806	807.4854	0

GQPPLAIL	808.5801	807.4854	0
GQQLRPGAAGQGTLTLPARGGCGRGAPADEVAAAG	808.5803	3230.633	0
GRGGAGSKRSRAAEVHNLS	637.4256	1908.999	0
GRGTPRGAATKAC	623.4101	1244.641	0
GRGTPRGAATKAC	623.4108	1244.641	0
GRGVPPV	681.4513	680.397	0
GRHRCEHICHVGPC	802.3647	1602.708	2
GRNAIETFN	511.3155	1020.499	2
GRRGSLHLIRAS	661.9583	1321.769	2
GRSLGRPEGVLINGKTAK	618.4541	1852.064	0
GRSLGRPEGVLINGKTAK	618.455	1852.064	0
GRVKIRGIKIEL	691.4706	1380.893	2
GRYKLSSQA	505.3488	1008.535	0
GRYQWKDG	505.3503	1008.478	2
GSFGRTFCKCWAC	367.1716	1464.61	0
GSGLLVGYWPAYLFS	544.0082	1628.824	3
GSGNGNSSAYPSSYGSSEYDTMQYN	656.5271	2622.009	0
GSKKGGGSLFSASAFDAIDDDADGEVVDDKN	772.582	3086.395	0
GSKRRKN	423.361	844.4991	0
GSKRRKN	423.3625	844.4991	0
GSKRRKN	423.3555	844.4991	2
GSKRRKN	423.3611	844.4991	0
GSKRRKN	423.3616	844.4991	0
GSLLRGLILLL	584.3801	1166.775	2
GSLMFFIGGLTNL	685.4353	1368.711	0
GSPGHS	271.0602	540.2292	2
GSPRRFMRT	554.3709	1106.577	2
GSRRKNK	423.3621	844.4991	0
GSRRRLRVF	573.9063	1145.69	2
GSSRGQSDNFPAVNTPSWASSNEIISESSEHSA	859.5993	3434.525	0
GSSTSIPGGNFSLNPPAAPTGTT	533.4507	2130.023	0
GSSTSIPGGNFSLNPPAAPTGTT	533.4516	2130.023	0
GSVKFHRFSKLQNLEK	959.5653	1917.059	0
GSVLSVAASRGELKLG	772.5242	1542.873	2
GSWVKKRKKVVG	686.4485	1370.851	2
GTHEYLAPEIIKGEGHGAAVDWWTFGVF	772.5815	3086.493	0
GTKAERFILGKKNKGGGVRRK	767.4547	2299.371	0
GTPALKRRTVI	606.3938	1210.751	2
GTRKR	617.4037	616.3769	0
GTRSPTKKRIK	636.4635	1270.783	2
GTSNNSKSS	441.2069	880.3886	0
GTSQRDDTAMSGTDQALSSSSEDSRN	676.4967	2702.132	0
GVALGVNTSYVSG	612.4135	1222.619	2
GVGPLLKLIK	519.399	1036.701	0
GVKEFEARW	561.3951	1120.567	0

GVKRAS	617.4037	616.3657	0
GVKRLLVP	441.3724	880.5858	0
GVKRLLVP	441.3726	880.5858	0
GVKRLLVP	441.3714	880.5858	0
GVLGQQLKA	457.3395	912.5392	2
GVLGSDLCTEDGVCTREGDAELNGDVGFDGS	772.583	3086.308	0
GVMNKRVKQ	530.4023	1058.602	0
GVMNKRVKQ	530.4023	1058.602	0
GVMNKRVKQ	530.4029	1058.602	0
GVNKVVAAANKAANTARDAAVKAVQ	813.462	2437.34	0
GVRGINYYNELQTDTSLAIEAKLMKR	746.5674	2982.56	0
GVTVKILLLWLNIVKVYRQ	752.5138	2254.393	0
GVVGLKLLGK	983.7097	982.6539	0
GVVLYEILTG	532.3572	1062.596	2
GVVPLWAGVVITAFD	772.502	1542.845	2
GWIGLSILLLYAVF	522.3449	1563.907	3
GYGGYGGSDSSYGNSGAYGTVGGRTGSAPNN	722.5031	2886.208	0
GYKKILVLYTN	656.4257	1310.76	2
GYKWRKYGKKTVK	821.5936	1640.952	0
GYLPFGFG	429.2804	856.4119	2
GYLSGEKGVHYMIRGSPNESSQLEASS	721.504	2882.351	0
GYNPPTI	381.1858	760.3755	0
GYNYGLTRASNYLNSYAQGYNMSPIGGYG	788.6058	3150.414	0
GYQGYMANHMATGHGAAGLGTFSEPGKANLETER	881.6119	3522.605	0
GYQKFSATPNYSS	367.1727	1464.652	0
GYRNDLAYLKSKVDAGADLIVTQLF	693.4746	2769.47	0
GYSGGDVKREITGVLFHQVCYVWQWK	764.5488	3054.517	0
GYSGGDVKREITGVLFHQVCYVWQWK	764.5483	3054.517	0
GYSHRERVRRGTR	544.0082	1628.872	3
GYSYQ	309.2033	616.2493	2
GYTITSSTSLVAIKKAICFH	535.9001	2139.14	4
HAAEILVEFFEKS	760.3215	1518.772	2
HACIIHR	425.2845	848.4439	0
HACTSC	311.1449	620.2047	0
HAEYECHGPG	367.1692	1098.419	0
HAGCNNNDVSWCSEEVSESKAE	599.4826	2393.949	0
HAGFNCG	353.1558	704.27	0
HAGFNCG	353.155	704.27	0
HAKCLEF	424.2717	846.4058	2
HAKCLEF	424.2741	846.4058	2
HAKNLHTL	467.3139	932.5192	0
HANGHANNEPGMAYQSI	453.3897	1809.785	0
HANGRLDKCQSIYV	535.3701	1602.794	3
HANNREANNTKFG	369.1863	1472.676	0
HANRLKSLPATFGN	509.3225	1524.816	3

HAQDDPFYLYQYCSSNR	527.4275	2105.89	0
HAQGSKMGPSSS	397.1822	1188.519	0
HARIFYDFARRRFA	609.4097	1824.965	3
HASGNYG	353.1546	704.2878	0
HATNKTS	380.2452	758.3559	2
HAVGCSNKFF	555.2747	1108.513	0
HAVGCSNKFF	555.2745	1108.513	0
HAVGCSNKFF	555.2704	1108.513	0
HAVLVETSIHISLLKASYHTSRQ	648.4639	2589.403	0
HCTTVLFNTEQRLYDSKLF	772.3166	2314.142	3
HDDSKLPSNPEEVEDLRRDS	585.4707	2338.067	0
HDNNHP	367.1712	732.2939	0
HDNNKRV	441.8273	881.4468	2
HDNRIHTVSRS	441.279	1320.665	3
HDQGGNTFHGLAPLQ	796.3735	1590.754	2
HDWASTSGPCGVTMFDEQLYA	772.3199	2313.967	3
HEARGIF	415.2111	828.4243	2
HECPKQ	371.2176	740.3276	0
HECSPRIVHRTF	371.2179	1480.736	0
HEKYAPLKLIIMSASL	605.4207	1813.017	0
HENAYQ	381.1875	760.314	0
HENSTEVPPSPASMCSCV	469.3839	1873.765	0
HETLSWNWPPWK	527.6784	1579.757	3
HFCFVCIMEWAKHESRCPICRQR	720.525	2878.322	0
HFLKIKEQKKYQ	398.325	1588.909	0
HFRQQPSPNPN	441.2072	1320.632	0
HGDGQC	309.1293	616.1911	2
HGDRTQQCA	339.1396	1014.43	0
HGEAGKTAIVI	548.3424	1094.608	2
HGEGNTF	381.1859	760.314	0
HGEGNTF	381.1873	760.314	0
HGFMTPKK	473.3407	944.4902	0
HGGMALETQGF	383.1679	1146.513	0
HGGMALETQGF	383.1644	1146.513	0
HGKGGSC	323.145	644.27	2
HGKNYGSS	425.2106	848.3777	0
HGKVKNELGN	548.3516	1094.583	2
HGLRFAVVVARF	686.4493	1370.794	2
HGNATENTDGDSEKES	423.3768	1689.671	0
HGNKILSL	441.3091	880.5131	2
HGNKKISLV	498.4093	994.5923	2
HGNKKLENAYEDA	497.2328	1488.684	0
HGNKNRR	441.3132	880.474	2
HGNNDFDFHDYDDTPPH	511.4313	2041.783	0
HGNSINR	399.2754	796.394	2

HGQQEKWFNNFHENLD	511.4308	2041.903	0
HGRERSPQIPYTGSL	425.2122	1696.865	0
HGRGGGALSGT	485.353	968.4788	2
HGRNARLYIIIAIVLTLILCL	595.3767	2377.44	0
HGSCTSCCKCA	367.17	1098.372	0
HGSWGKLGGGDGDEEQQGG	468.3878	1869.788	0
HGSWGKLGGGDGDEEQQGG	468.3876	1869.788	0
HHSHFRTKKTFSPFIMKTRRSCR	963.5959	2887.508	0
HHSSSSTNPGFLDALRSG	935.4463	1868.877	2
HIIHHSRH	518.8427	1035.548	2
HIMSLAKSF	517.3688	1032.543	0
HIQPRYCLKCG	439.8961	1316.648	3
HIRNSPPPQ	523.3565	1044.547	2
HIRPGTTTLNIVIH	786.507	1570.894	2
HITDIVPYTGQCQEEEEEEEESDN	941.7005	2822.135	0
HKNSGMIF	467.3138	932.4538	0
HLACKYL	424.2704	846.4422	2
HLENLRNQLNKC	494.6469	1480.757	3
HLKTIRQKGA	576.4098	1150.694	0
HLKTLKPFSQKF	737.4986	1472.85	0
HLLKITGKGVRGC	691.4508	1380.802	2
HLMIIIYS	495.3898	988.5416	0
HLNGAEGYA	311.1435	930.4196	0
HLNNVNKNDGGTAQNAGS	453.389	1809.835	0
HLPIHLAGKRGHVE	782.5003	1562.879	2
HLPIHLAGKRGHVE	782.4903	1562.879	2
HLQDSAF	409.1613	816.3766	0
HLQHKVRN	516.386	1030.579	0
HLSEDMIFLVF	338.3417	1349.669	0
HLYIVELIRAGIF	772.5015	1542.892	2
HMQSQAAGANGLVEGAVMESSGTENCQRN	992.7431	2975.292	0
HNFHPQAYNLCLHGLFGSLF	772.3166	2314.111	3
HNINASALRTMK	678.4592	1354.714	2
HNNNNNNNNSNCSSGYSSYGG	555.4573	2217.8	0
HNPEFRRYADCSA	783.3462	1564.684	2
HNRLKLRISRMF	786.5011	1570.888	2
HNSNTSFV	453.2078	904.4039	0
HNSNTSFV	453.1668	904.4039	0
HPALVK	664.4626	663.4068	0
HPARVPMWK	561.395	1120.596	0
HPCCAM	339.1404	676.2131	2
HPKPQAAK	438.787	875.4977	2
HPPPPIPQEPR	632.9174	1263.672	2
HPSSSM	323.1454	644.2588	2
HPSSSM	323.1453	644.2588	2

HPTLDS	335.2181	668.3129	0
HQNQAY	381.1869	760.314	0
HRHNGVSALV	545.3703	1088.584	2
HRIAPIVSLSG	575.4085	1148.667	0
HRRVYERRKR	728.4972	1454.844	2
HSGMANSTRSRSPISPRRTHNAIGSNEH	765.1543	3056.482	0
HSNNNIA	385.1807	768.3514	0
HSQHINMHYA	413.2647	1236.546	0
HTKDDAELLVEK	699.405	1396.72	0
HTKGCKCPPGFRGDGVHSC	497.2342	1984.882	0
HTKKVILDTAKR	705.5084	1408.851	0
HTNELLLGKALKGGV	388.2536	1548.899	0
HTNKSGGF	424.2725	846.3984	2
HTNKSGGF	424.2713	846.3984	2
HTNRHRTGAK	393.2857	1176.622	0
HTQNSIDF	481.2374	960.4301	0
HTSEVSKEDNSGKSSNNLN	512.4134	2045.925	0
HTVCTSFS	441.2663	880.3749	2
HTYAKLA	402.2576	802.4337	2
HVATSTDS	409.1623	816.3614	0
HVFEPH	383.1672	764.3606	0
HVFKKNPKAPI	639.9449	1277.761	2
HVGSFSQ	381.1862	760.3504	0
HVILGRTVEVKKAIPRSEQ	540.8618	2159.254	4
HVKELKRQTSLIAE	551.3677	1650.942	3
HVKLSNLCSNSHIMD	425.2136	1696.803	0
HVKLSNLCSNSHIMD	425.2114	1696.803	0
HVKPV	579.3829	578.354	0
HVLRQFYRECD	367.1704	1464.693	0
HVNEVEGLQIKK	465.2966	1392.773	3
HVNNDDVVSATC	425.2122	1272.54	0
HVNNDDVVSATC	425.2129	1272.54	0
HVNSKYCI	482.3031	962.4644	2
HVNVVRLLGFCADGFH	595.3783	1782.899	0
HVPAVVMQ	441.2631	880.4477	2
HVQAGQCGNQIGGKFWEVMCDEHGIDATG	772.5821	3086.343	0
HVSKNLRKGG	548.3508	1094.631	2
HVSNNVVVMFAG	425.2133	1272.629	0
HVSNNVVVMFAG	425.2123	1272.629	0
HVVANLT	753.4716	752.4181	0
HVYVFGGC	441.2627	880.3901	2
IAATLVGLQSVPGLII	522.346	1563.96	3
IADEHVGHDDFYNTKEASHMPSPGTAGLF	786.5986	3142.409	0
IADLLREYVDSGDTLEACRCIRELGVSF	786.5978	3142.543	0
IAIGRIQIVPRFA	485.3379	1452.893	3

IAKAAGILRARLGRKNTEQKDN	808.5791	2422.388	0
**IAKKLVLP**[d]	441.3728	880.611	0
**IAKKLVLP**[d]	441.3731	880.611	0
**IAKKLVLP**[d]	441.3731	880.611	0
**IAKKLVLP**[d]	441.3731	880.611	0
IAKKSKGG	788.6034	787.4916	0
IALSRSRERRKR	764.5496	1526.923	0
IAPLPPGWEKRSDAVTGK	961.5816	1921.042	0
IARKDLVPFDPGFPFKF	499.2473	1993.083	0
IARNLLASSFVVTL	752.4873	1502.882	2
IASVKLRIQTFKGFFVKKQ	560.4126	2237.341	0
ICLRDRLVTL	601.4376	1200.701	2
IDCGNSYYMPPKPRPSTCN	536.486	2141.933	0
IDETLTSDPVNYT	734.3203	1466.678	2
IDFPIVGGK	473.3423	944.5331	0
IDLNNTDQIKREI	786.3582	1570.832	2
IDTDSGRGVDTNSSEYM	462.4493	1845.769	0
IEKWILRR	557.4118	1112.682	2
IEKWILRR	557.4118	1112.682	2
IEPGLKVPL	483.3757	964.5957	2
IEPNLKHYGCVI	693.3795	1384.717	2
IETLRERVF	581.9031	1161.651	2
IFADNSNL	447.3059	892.4291	0
IFALSLSLIIPTVLAATA	605.4211	1813.096	0
IFILRPQS	487.326	972.5756	2
IFIQEAKYSFHLVQPGFHWIRLYF	760.5838	3038.596	0
IFIRKKAAERRAL	786.6035	1570.978	2
IFKDKKVVIFGLP	760.4844	1518.917	2
IFKSIFCLPCLLF	772.5119	1542.834	2
IFKSPLLDLLIKSG	772.4872	1542.939	2
IFLRSLKVKKALVKS	577.3688	1729.134	0
IFLVYIFSFLRN	766.5302	1530.86	0
IFMMYGAYSTTRRSAVSRIF	786.3359	2356.182	3
IFPTSLKKTG	546.3992	1090.639	0
IFPTSLKKTG	546.3964	1090.639	2
IFSKPCSPSRLCPFQLCVFDTKQVLSSG	772.5837	3086.539	0
IFTCSFTPGSCSLTAYSLTFPPIMRRFR	800.6101	3198.582	0
IFTVLK	720.5248	719.4582	0
IFVYPTKN	491.3724	980.5331	2
IFYKYN	424.2717	846.4276	2
IGAPPVIVLSSACA	433.2907	1296.711	0
IGKVLLSLVSVFF	711.4624	1420.869	0
IGLLAVPFLEN	593.441	1184.681	2
IGLLVKKFLIKINRN	590.4257	1768.145	0
IGNLTKRHH	538.3438	1074.605	2

IGSGKKNRKR	572.4003	1142.7	2
IGTPISRSS	459.3455	916.4978	2
IGVILRDSH	505.3503	1008.572	2
IHACDYK	425.2113	848.3851	0
IHCEDPTAIRKCPNNFA	483.2178	1928.887	0
IHGDSEMG	423.1962	844.3385	0
IHGLIVLLKKVT	667.4776	1332.886	2
IHGMGGVGKSTLARAVYNLHTDH	609.3947	2433.234	0
IHIARTGWFGAGLV	749.5216	1496.825	2
IHKLLKPH	493.3847	984.6233	0
IHLACKKGHVRVIN	794.564	1586.919	0
IHLGTISGLGSS	381.1863	1140.614	0
IIALSTIVLVGV	599.3932	1196.774	0
IIDPAIGLASTTLTLSGFDKFV	760.3191	2278.246	3
IIDVIVH	808.5809	807.4854	0
IIIVPRNSMSKDF	760.4857	1518.823	2
IIKKTPVVVGN	584.3698	1166.739	2
IIRALVGIISH	596.3898	1190.75	2
**IIRCTGC**[d]	383.1669	764.3673	0
**IIRCTGC**[d]	383.1652	764.3673	0
**IIRCTGC**[d]	383.166	764.3673	0
**IIRCTGC**[d]	383.1665	764.3673	0
**IIRCTGC**[d]	383.1668	764.3673	0
**IIRCTGC**[d]	383.1668	764.3673	2
**IIRCTGC**[d]	383.1663	764.3673	0
**IIRCTGC**[d]	383.1665	764.3673	0
**IIRCTGC**[d]	383.1644	764.3673	0
**IIRCTGC**[d]	383.1654	764.3673	0
**IIRCTGC**[d]	383.1652	764.3673	0
IISPGARQGLIPLAIP	808.5795	1614.982	0
IITGKFPSQY	385.2911	1152.618	0
IITLELLSTH	570.3698	1138.66	2
IIVYLVELLK	601.9441	1201.769	2
IIYTLVLI	474.3148	946.6103	2
IKAALARR	449.8242	897.5872	2
IKAHVPLRFA	576.3879	1150.698	2
IKELPASIGSL	564.3589	1126.66	0
IKFKVGTQI	517.37	1032.633	0
IKGMKGNRAF	561.3962	1120.618	0
IKGQWNKEEDRKLIRLVKQ	794.5633	2380.37	0
IKHKLGNMASI	606.3938	1210.686	2
IKKRVFSKWDQVKDPEPEPESESESKSTVN	876.659	3502.758	0
IKKSAKVRT	515.8644	1029.666	2
IKLGVLVEGLHKRCS	551.3589	1650.96	3
IKLLCSQVRF	603.9162	1205.695	2

IKLLRHADKN	604.4217	1206.72	2
IKNALVRAGAKH	639.4431	1276.773	2
IKNAVGRP	427.8114	853.5134	2
IKSKLSIGGTA	537.8771	1073.644	2
IKSKLSIGGTA	537.8772	1073.644	2
IKTFFPVEATSTSFQSYPPAPPDLGHQDLRLSLQS	969.732	3874.942	0
ILALIVSVRPKN	661.9582	1321.845	2
ILALIWVWKK	635.412	1268.801	0
ILALIWVWKK	635.414	1268.801	0
ILCDIDIILLMFAPNGKPSLCRGRHSNF	786.5979	3142.624	0
ILELLLQIPL	582.874	1163.753	2
ILGLAKKN	428.8013	855.5542	2
ILGVPKLRATRTVF	786.0116	1569.972	2
ILGVPKLRATRTVF	786.0134	1569.972	2
ILGVPKLRATRTVF	786.0121	1569.972	2
ILGVPKLRATRTVF	786.0121	1569.972	2
ILHEISPASNNSSE	749.3179	1496.711	2
ILKKNNSF	482.3006	962.5549	2
ILKKNNSF	482.3076	962.5549	2
ILKPVFTQR	551.3518	1100.671	0
ILLGVVF	760.5826	759.4894	0
ILLLQGHLGRR	638.4378	1274.794	2
ILQRLLEEREKAANMDEERGEEPEKY	794.5638	3174.562	0
ILRIVF	760.5831	759.5007	0
ILRLTKN	429.2805	856.5494	2
ILRTGKSIN	501.3514	1000.603	2
ILSAISNK	423.3612	844.5018	0
ILSGPMQSVGNHGGRNNPD	975.4387	1948.917	2
ILSLVIPDK	499.3707	996.6219	2
ILSRPKLSSAISLN	500.315	1497.888	3
ILSWVLNVSKVLK	500.3179	1497.928	3
ILSWVLNVSKVLK	500.3179	1497.928	3
ILSWVLNVSKVLK	500.315	1497.928	3
ILSWVLNVSKVLK	500.315	1497.928	3
ILTLFTSS	441.2957	880.4906	0
ILTLFTSS	441.2972	880.4906	0
ILTLFTSS	441.2999	880.4906	2
ILTLKYPIEHGIVSN	566.3714	1695.956	3
ILVLSRTN	458.3033	914.5549	2
ILVPGHRYILISFRHVEKLKRKLLEEEKNKEATGVVLETK	943.5485	4712.722	5
ILVPRWWNN	599.3914	1196.646	0
ILVVDALYIG	538.3438	1074.632	2
IMLVFPLLNF	603.9162	1205.688	2
IMSLDS	333.1674	664.3102	2
INALKGVCRMS	596.3898	1190.626	2

INLSHCS	387.1799	772.3538	2
INSKAGKKIFS	596.908	1191.698	2
INVNIDSRDVA	608.3836	1214.626	0
INVVRLLGYCAKGIHRALVYNFFPNGSLQSIIFP	955.754	3819.081	0
INYKLMKK	519.3917	1036.61	2
IPAAAQSPALISH	638.4367	1274.698	2
IPAAAQSPALISH	638.4378	1274.698	2
IPAAAQSPALISH	638.4378	1274.698	2
IPAAAQSPALISH	638.4378	1274.698	2
IPAAAQSPALISH	638.4378	1274.698	2
IPALKRKR	491.3723	980.6607	2
IPCQRFALNIF	441.2976	1320.701	0
IPDGHRA	383.1657	764.3929	0
IPETFSST	441.2067	880.4178	0
IPFLWHVF	529.8806	1057.575	2
IPGDNFC	383.1666	764.3163	0
IPGIDIHLN	496.3276	990.5498	2
IPKLVRLGPLLEHT	529.3612	1584.972	3
IPKYLRKKLA	615.4544	1228.802	2
IPLDGRFIRKCKQ	795.4202	1588.887	2
IPLISNGAHAIGL	638.4367	1274.735	2
IPLPELQGLKTL	661.4837	1320.802	0
IPLVMFIGVN	551.893	1101.626	2
IPNDHCLTLLPLVDS	413.2641	1648.85	0
IPNIWFFFQFLGSTT	455.3122	1816.919	0
IPNLFSC	397.1809	792.384	0
IPNNFHLKGV	569.8592	1137.63	2
IPPLVISGSM	507.3359	1012.563	2
IPPPKKLKRK	610.9236	1219.813	2
IPQFYPNYTFTTHDLSEYPVPLLNGG	746.5642	2982.444	0
IPRGKGRAC	479.3268	956.5338	2
IPRNNFVGQVPIGTHI	441.2965	1760.969	0
IPRSSNSSN	481.2391	960.4625	0
IPSPSEKGLLASCKTLRHVKQIHAQ	686.1771	2740.517	0
IQKDPLILSRIS	691.9681	1381.829	2
IQLPSGVKKLI	598.3967	1194.77	2
IRGKQNGKPKGS	635.4152	1268.731	2
IRGNKGYRISS	625.9298	1249.689	2
IRLAKQTEKFK	681.4926	1360.819	2
IRLLAKRNADNR	720.4721	1438.848	2
IRQLQSTQ	487.329	972.5352	2
IRSFIIFL	504.8725	1007.617	2
IRVQNSRQADVVF	511.3525	1530.827	3
IRVTTHCRR	571.4282	1140.63	2
IRVTTHCRR	571.4282	1140.63	2

IRYRLWRN	588.8909	1175.668	2
ISDNVRSKASELTKLLSHPQSF	615.1352	2456.302	0
ISFQNGFA	295.1497	882.4236	0
ISIFNKIS	461.3176	920.5331	2
ISKQNCT	397.1832	792.38	0
ISKQNCT	397.1818	792.38	0
ISLHRR	781.4693	780.4719	0
ISPDDDWFRETSIHSLQ	682.6306	2044.949	3
ISPRVRVRACVAS	707.4881	1412.804	0
ISRIMKKALPPN	684.4717	1366.812	2
ISRPFKKRNKN	694.4474	1386.821	2
ITAKSIPSH	477.3178	952.5342	0
ITGNKRPSH	505.3503	1008.546	2
ITRPPSA	371.2272	740.4181	2
ITVLIDRSLLKVE	750.0111	1497.913	2
IVAAKKMS	424.2741	846.4997	2
IVAAKKMS	424.2741	846.4997	2
IVAHRLSTVRN	633.4165	1264.737	2
IVDCIVQMIKCKVGSIKSGWRSVFMIF	772.5819	3086.631	0
IVDKQRLLIKK	677.4432	1352.887	2
IVEAHAW	413.2641	824.4181	0
IVGVVGSCSHS	522.8693	1043.507	2
IVIKDMLNHH	407.2381	1218.654	0
IVILGVF	760.5825	759.4894	0
IVIYKKPFPSRRTWLYGYMNRDRDYELENGLALFGS	1092.845	4367.231	0
IVKDIVSKNVIVQ	727.998	1453.887	2
IVKKRKPYHH	653.4205	1304.783	0
IVKTLNASVTQHTLEIHFYWAGKGTTGIPTR	860.6639	3438.841	0
IVLLLQGLK	997.6873	996.6583	0
IVLVIAVKVGILICVCRRRN	746.5658	2236.375	0
IVPCYGRDYGRIMEDPKGNLLRGVGNF	760.5829	3038.522	0
IVPGNNNSF	481.2386	960.4665	0
IVTYNILLRAF	661.9583	1321.776	2
IVVIAVF	760.583	759.4894	0
IVVMQKLG	444.3045	886.531	2
IWNCLRDGDFEEEEVWAVF	786.3332	2356.047	3
IWNCLRDGDFEEEEVWAVF	786.3396	2356.047	3
IWNCLRDGDFEEEEVWAVF	786.3396	2356.047	3
IWNCLRDGDFEEEEVWAVF	786.3396	2356.047	3
IWNCLRDGDFEEEEVWAVF	786.3353	2356.047	3
IWNCLRDGDFEEEEVWAVF	786.3353	2356.047	3
IWNCLRDGDFEEEEVWAVF	786.3445	2356.047	3
IWNCLRDGDFEEEEVWAVF	786.3445	2356.047	3
IWNCLRDGDFEEEEVWAVF	786.3375	2356.047	3
IWNCLRDGDFEEEEVWAVF	786.3359	2356.047	3

IWNCLRDGDFEEEEVWAVF	786.3359	2356.047	3
IWNCLRDGDFEEEEVWAVF	786.3359	2356.047	3
IWNCLRDGDFEEEEVWAVF	786.3393	2356.047	3
IWNCLRDGDFEEEEVWAVF	786.3392	2356.047	3
IYGANCPQW	351.1755	1050.459	0
IYLADRRSTG	576.3834	1150.61	2
IYLVQLLN	488.3311	974.5801	2
IYNGS	277.1797	552.2544	2
IYNGS	277.1796	552.2544	2
IYSFYKDIENSS	367.1709	1464.677	0
IYSFYKDIENSS	367.1708	1464.677	0
KAAHSVL	725.4802	724.4232	0
KAALPRGT	813.5314	812.4868	0
KAARGRP	755.4871	754.4562	0
KAATNNFDPANK	430.9041	1289.637	3
KAFNKDKA	461.3294	920.508	2
KAFNKDKA	461.3176	920.508	2
KAFNKDKA	461.3234	920.508	0
KAFNKDKA	461.3294	920.508	2
KAHRLARQ	490.3563	978.5836	2
KAILSELRHLNQ	711.504	1420.815	2
KAKHQNKKKKKNKK	579.3833	1735.106	0
KALKQAVQ	443.3029	884.5444	0
KASFRKM	434.3223	866.4796	0
KASPPTSSPPTTSPV	727.4592	1452.746	0
KASRVTRRKLSR	729.4785	1456.906	2
KASTNK	648.4652	647.3602	0
KASVVSLGDLNVKIV	771.4852	1540.919	2
KAYVI	593.4002	592.3585	0
KCASKYNQGTGMCSTIGNNNLC	577.4414	2305.991	0
KCGKLGNN	417.292	832.4225	2
KCGNIRDACKFFNTISDQA	533.4505	2129.999	0
KDDASVNILQTCILCEKKYHNSCTKE	746.5672	2982.425	0
KDRPYLLHTNI	685.4283	1368.751	2
KEKCYRDAACRGLDFMFGWYMGPLIKGEYSKT	941.7005	3762.782	0
KEKRKEGGHGGGP	668.9478	1335.701	2
KESLLNLRVAPHFG	527.6783	1579.883	3
KEVTVIEW	335.2177	1002.539	0
KFEQKGDKFVSKQVDNKKKKKL	663.4625	2649.533	0
KFGAVEDVVIKGSK	492.995	1475.835	3
KFGQFLARTLVRQ	782.5003	1562.905	2
KFIIIKEFKKE	712.0105	1421.865	2
KFIVKDRSH	565.4075	1128.64	2
KFKGKKW	461.3176	920.5596	2
KFLDISDNQDLGGSL	811.4214	1620.8	2

KFLKSGKWGI	582.4087	1162.686	2
KFQLRKAS	489.3072	976.5818	2
KFQPNLGSQNEVKE	809.4126	1616.816	2
KFVPFTRHSAQTT	507.3263	1518.794	0
KGEIFSVGKRR	638.9422	1275.741	2
KGEIPLL	769.5051	768.4745	0
KGFSEVANK	490.3563	978.5134	2
KGGAGKRLRKD	593.441	1184.71	2
KGGGGPVFVGQV	551.391	1100.598	2
KGGIQIWNW	551.3516	1100.577	0
KGGIVKDP	813.5314	812.4756	0
KGGKDLLLIKTKGG	714.4522	1426.887	2
KGKGKAAAFKRR	659.4801	1316.815	2
KGKKFKF	441.8273	881.5487	2
KGKKFKF	441.8273	881.5487	2
KGKLIGRGTFGSVFH	535.3588	1602.899	0
KGKVHSGI	413.266	824.4868	0
KGMLTGPVTILNWSFVRN	678.4742	2032.093	0
KGPWKRKKIKN	691.9694	1381.867	2
KGQVNKALHFH	639.943	1277.699	2
KGQVNKALHFH	639.9446	1277.699	2
KGSALSVLGR	494.3191	986.5873	2
KGSLPDGREVAVK	678.4593	1354.757	2
KGSSDILPLVKVQLKGS	590.425	1768.046	0
KGTFPKSCLLN	604.4393	1206.643	0
KGTVVTGGP	815.512	814.4549	0
KGVHVPQ	764.5493	763.4341	0
KGVRKSNKGG	515.8644	1029.604	2
KGVTGKQAP	443.3019	884.508	0
KGYGLTVSGR	519.344	1036.567	2
KHANIT	683.4255	682.3762	0
KHCNEATY	483.2541	964.4073	2
KHLKSL	725.4805	724.4595	0
KIAPIQVVIVPI	645.4649	1288.848	2
KIDKLSKVNHKNFV	835.5831	1668.968	2
KIFLNAVKA	502.373	1002.623	0
KIFTRCS	427.8114	853.448	2
KIIKRVVKQK	620.4359	1238.855	0
KIIKSH	725.4792	724.4595	0
KIIKSH	725.4794	724.4595	0
KIPKSLSNCTT	596.3547	1190.633	2
KIQAAFRGSLAR	659.4801	1316.768	2
KISRKHSSRKR	691.9694	1381.838	2
KIVKNAHK	469.3843	936.5869	0
KIVKNAHK	469.3844	936.5869	0

KIVKNAHK	469.3835	936.5869	0
KIVKPRPVQAA	603.9157	1205.761	2
KIVPVIIFFH	606.8972	1211.743	2
KIVTSKKVVG	529.8806	1057.686	2
KKALPPNGKIAK	632.9174	1263.803	2
KKATKFAGF	499.3528	996.5756	2
KKDARKHG	939.5948	938.541	0
KKFIVSWK	518.3412	1034.628	2
KKFLMKNRN	589.8901	1177.675	2
KKFLNALGKRYQVS	551.3677	1650.957	3
KKFRGWKKR	617.4026	1232.762	0
KKFRPIRSS	559.8898	1117.672	2
KKFRQNHLKMF	492.995	1475.818	3
KKGALLRGGKAM	615.4544	1228.744	2
KKGHEVLVQGG	576.3842	1150.646	2
KKGSGGITIKKTGQAL	794.0364	1585.952	2
KKGYKQTT	477.294	952.5342	0
KKHH	549.3716	548.3183	0
KKILSH	725.4806	724.4595	0
KKIRKVVIRK	423.361	1266.898	0
KKKIKTPV	941.6995	940.6433	0
KKKILQHQPVLIPGTKIKALQ	794.5617	2380.505	0
KKKKDDKKK	573.4016	1144.729	2
KKKKDDKKK	573.4016	1144.729	2
KKKRKKKKN	593.4408	1184.819	2
KKKSA	561.398	560.3646	0
KKKTKLQSPNFH	728.4804	1454.836	2
KKLAKKYH	508.3344	1014.634	2
KKLRTILLLGKTNKSRLPHEVKMA	925.6382	2773.684	0
KKLRVYVSHKNLRVRAKAAVS	808.5814	2422.476	0
KKNANKSS	438.834	875.4825	2
KKNKTTNKKKDA	702.434	1402.826	2
KKQYELKFILNKHH	609.4097	1825.036	3
KKRIH	681.4527	680.4446	0
KKRKKKTY	540.354	1078.698	2
KKRVRFA	452.8041	903.5767	2
KKRVSHA	413.3217	824.498	0
KKRVSHA	413.3241	824.498	0
KKSKNPNK	472.3604	942.561	0
KKSKNPNK	472.3359	942.561	2
KKSKNPNK	472.362	942.561	0
KKTKKPKL	485.8537	969.6699	2
KKVEKKLRKLP	683.9714	1365.918	2
KKVSKAPK	443.3026	884.5807	0
KLAKKQVK	471.8371	941.6386	2

KLAKSLDSCNPTEQHV	443.3328	1768.878	0
KLAQRMTN	961.582	960.5175	0
KLCKFV	737.4983	736.4305	0
KLESLFKDLREAGYNPTTEF	786.6649	2357.19	3
KLFDCKQ	441.2663	880.4477	2
KLHKFP	769.5055	768.4647	0
KLHKFP	769.5057	768.4647	0
KLHLIEWSKN	634.4502	1266.709	0
KLLHIRVAGFV	647.9318	1293.792	2
KLLKSH	725.4797	724.4595	0
KLLLLLPVDH	580.8969	1159.733	2
KLPILVYFH	565.4075	1128.67	2
KLPILVYFH	565.4102	1128.67	2
KLPIVLHFH	552.3599	1102.665	2
KLPIVLHFH	552.3599	1102.665	2
KLPLLVYFH	565.4103	1128.67	2
KLPPAKRK	469.3596	936.6233	2
KLQSVEQKQKEWPNAKNPRGEKRVCNARMKRELG	1009.75	4035.148	0
KLRDLRLFEN	652.4235	1302.741	2
KLRRCCHTEIVTVGPAKKEKEKEKVK	760.1953	3036.705	0
KLSERILK	493.8504	985.6284	2
KLTSVDGGCSLREHGLLCDEEIHARGSSIFTLEMACSS	1013.693	4050.891	0
KLVFTGVIVV	537.8772	1073.685	2
KLVLEKQQKK	621.4098	1240.787	2
KMKKK	662.4808	661.4309	0
KMLIELVK	487.329	972.6042	2
KNEIQRLQRIL	705.9837	1409.847	2
KNGGKKNKQKNK	686.4485	1370.811	2
KNHISVPCPKVADVMLVGGIQN	580.5118	2318.224	0
KNKKKSKKK	558.8851	1115.75	2
KNNRSSLSIFI	639.9438	1277.709	2
KNNSIALVRCASML	507.3302	1518.801	3
KNNSS	549.375	548.2554	0
KNPEITVH	469.3595	936.5029	2
KNPEITVH	469.3597	936.5029	2
KNQGHSAN	428.3026	854.3995	2
KNRRSCGF	484.2982	966.4818	2
KNVLSLHV	455.3105	908.5444	0
KNYEFKKI	535.3994	1068.597	2
KNYEFKKI	535.3578	1068.597	0
KNYEFKKI	535.3997	1068.597	2
KNYEFKKI	535.3997	1068.597	2
KPIDRLDIVSRIF	786.5068	1570.92	2
KPIDRLDIVSRIF	786.5068	1570.92	2
KPIDRLDIVSRIF	786.5068	1570.92	2

KPIVITPNKTY	637.4669	1272.744	2
KPLRIRLGGSL	605.4205	1208.772	0
KPPAH	549.3764	548.3071	0
KPPAH	549.3754	548.3071	0
KPPAH	549.3756	548.3071	0
KPPAH	549.3752	548.3071	0
KPPDH	593.4014	592.2969	0
KPRQHIKI	510.3441	1018.64	2
KPSWRILN	507.3506	1012.582	2
KQIFRQVD	517.3694	1032.572	0
KQINIEVEGLSVVKYYLSDYGYIESSGPFNNI	910.6649	3638.803	0
KQKKRKKSKLTT	737.5017	1472.952	0
KQVEIKRTIPRGAAGSNSKDFRTKKIFV	794.5652	3173.815	0
KQVNASRIVLQ	628.3962	1254.741	2
KRLVKVSKVMGKSGSRFGSGNNGSGMKSGKGSPVKSM	939.5971	3753.992	0
KRNLPGN	399.7809	797.4508	2
KRPPLR	766.5302	765.4973	0
KRQRRSKPCTDV	737.5011	1472.799	0
KRRLLQKK	535.3997	1068.724	2
KRRLVH	808.5794	807.5192	0
KRRRISIFSSSSIG	797.3939	1592.911	2
KRRVRGVLN	549.4149	1096.694	2
KRSVLANR	472.3303	942.5723	2
KRTKSKPKAQKALS	794.0364	1585.963	2
KRVTESLLSVLLL	735.9954	1469.918	2
KSANKKIFRFA	655.3788	1308.767	0
KSASKISRTLSPKTEGCYKKKRVTVETKVVMPKGKLNKSASA	1141.828	4563.572	0
KSATKN	648.4651	647.3602	0
KSFIKLWASK	604.4387	1206.713	0
KSFIKLWASK	604.4384	1206.713	0
KSFIKLWASK	604.4388	1206.713	0
KSHRTSRNRK	635.4152	1268.717	2
KSKKGS	634.4512	633.381	0
KSKKK	618.4543	617.4224	0
KSKKK	618.4554	617.4224	0
KSKKK	618.4552	617.4224	0
KSKKSARVQALIDQ	786.5385	1570.916	2
KSRKKKPEKKNGKKG	871.1207	1740.085	2
KSSHH	595.4042	594.2874	0
KSSPTEAGT	439.2769	876.4189	2
KSSSSSFSH	477.3173	952.425	0
KSVKNGRP	443.3322	884.5192	0
KSVVFIAQKQ	574.4295	1146.676	0
KSWNIGPVSA	529.8798	1057.556	2
KTAQGLLE	430.2892	858.4811	2

KTASKK	662.4801	661.4123	0
KTASKK	662.4808	661.4123	0
KTCGTR	333.1669	664.3326	2
KTGRLIISGLIYTE	782.5003	1562.903	2
KTGWPIVGVG	507.3645	1012.571	2
KTHKLHSH	494.3195	986.541	2
KTKKQLKP	485.854	969.6335	2
KTKNSSGRGKRV	659.4801	1316.764	2
KTLKHHRRSK	645.9706	1289.779	2
KTSSKEKQQ	532.3572	1062.567	2
KTVELRKQGVRFV	520.6935	1558.931	3
KVARTSFT	455.3105	908.508	0
KVDAAGKLPRRK	669.9557	1337.826	2
KVDYVFKVVL	605.4185	1208.717	0
KVEKKKKKKKV	750.0113	1498.045	2
KVGKLQGEK	493.8508	985.592	2
KVGRIIIQKTSMF	507.6702	1519.891	3
KVIDYIKRVY	648.9572	1295.76	2
KVLKVKHNSNLS	683.9713	1365.809	2
KVLVATKAKPN	584.9265	1167.734	2
KVPKPLKFLQQ	663.449	1324.823	2
KVPKPLKFLQQ	663.449	1324.823	2
KVRIPVLANGHRR	506.0185	1514.927	3
KVVGGMSFG	441.3091	880.4477	2
KVVNELVPNQ	570.3536	1138.635	2
KWAAKKALSLGV	636.4652	1270.776	2
KWRGSTRLLL	615.4544	1228.741	2
KWRGSTRLLL	615.4544	1228.741	2
KWRKLF	439.2902	876.5334	2
KWSFHV	402.2588	802.4126	2
KWSHKIEALII	669.4155	1336.787	2
KWVRLLYN	546.3968	1090.629	0
KYEIGKKFG	535.3994	1068.597	2
KYEKPLQDLK	421.3157	1260.708	0
KYGRVKHIFV	623.9572	1245.735	2
KYIKVKVRGKRSEKLQETMP	605.4387	2417.394	0
LAATAVDFPKTG	595.9193	1189.634	2
LADRILKTWPGIHRALNSTRITLLSDEILVQKLFFELFPF	786.172	4710.642	6
LAFLNLAQ	445.3332	888.5069	0
LAIDSGRHP	483.3032	964.509	2
LAKHLHFCEVCGKGFTRDANLRMH	700.5532	2798.368	0
LAKKQGLK	443.3014	884.5807	0
LAKKYMKRVA	604.4217	1206.727	2
LALCFLLRHLNRR	813.0806	1623.951	2
LAPTSCSS	383.1666	764.3375	0

LAQQKEY	440.2733	878.4498	2
LARELTCDIEFGKSLLVTGPNGSGKSSIF	760.5839	3038.575	0
LARRFALVPLGPPL	760.4823	1518.94	2
LAVFIARIGK	544.413	1086.691	2
LAWLVIEAGVGVAVF	772.5224	1542.881	2
LAWNFFYDVCPFEKIELNASKVYLSF	786.5985	3142.552	0
LCNNFVKK	965.5077	964.5164	0
LCNNFVKK	965.51	964.5164	0
LCNNFVKK	965.51	964.5164	0
LCQINKLPGSMKNTC	413.2621	1648.81	0
LCVENSMRHHCPICYEYLF	786.3332	2356.026	3
LCVENSMRHHCPICYEYLF	786.337	2356.026	3
LDIVPFDSCSKEYQNLYEEWFNYADA	794.5605	3174.38	0
LDLGRYNLGGTLAPELAH	637.4269	1909.006	0
LDLNGNNFS	497.2347	992.4563	0
LDNALKIFRR	623.4519	1244.735	2
LDPRVNRNPFTG	693.3832	1384.721	2
LDTGHCK	387.1799	772.3538	2
LDVVAYSS	427.2791	852.4229	0
LEAMKKARR	551.893	1101.644	2
LEAMKKARR	551.893	1101.644	2
LEASMLWRKR	645.4635	1288.707	2
LEAVRLAQMGNWSE	535.3577	1602.783	0
LEDVTSNRNNGNCS	381.3332	1521.648	0
LEFSFGVIPNVRAKGK	588.0316	1760.994	3
LEIALPAFRPKSQISNSS	653.4001	1957.063	0
LEKERAAAATAADETMAMISRLQEEKASMELE	881.6136	3522.7	0
LEKKELSKPFA	645.4649	1288.739	2
LELHQK	767.4534	766.4337	0
LEMVKAKIKAA	601.4376	1200.726	2
LERRNSIKKLS	672.4336	1342.804	2
LETYVFAMFDENQKQPEFEKFWGLF	786.5984	3142.479	0
LETYVFAMFDENQKQPEFEKFWGLF	786.5992	3142.479	0
LETYVFAMFDENQKQPEFEKFWGLF	786.599	3142.479	0
LEVLVISAQKGHGMQFPKGGWETDESMEQ	808.5814	3230.538	0
LFALLDIEKVKILG	786.6035	1570.97	2
LFALVAKQPT	544.413	1086.644	2
LFAPAYAGIVTS	605.4204	1208.644	0
LFAWRLFIKQ	661.4582	1320.771	2
LFHNLFLEALDLV	772.4872	1542.845	2
LFHNLFLEALDLV	772.5119	1542.845	2
LFIPNCS	397.1816	792.384	0
LFIPNCS	397.1814	792.384	0
LFIPNCS	397.1809	792.384	0
LFIPNCS	397.1801	792.384	0

LFKERNYFMKIT	795.4202	1588.844	2
LFKKWRR	517.3695	1032.635	0
LFKMLLEKAYN	685.4283	1368.748	2
LFLARDAVILA	601.4387	1200.723	2
LFLDEAPFNFNPF	786.4104	1570.734	2
LFLIIVAIVIVALF	772.5806	1543.015	0
LFLLGLQNVGKGNWRGISRN	561.3943	2241.25	0
LFLPPPLSF	515.8631	1029.59	2
LFLPQIKLPQTFV	772.486	1542.917	2
LFPWIHAS	485.8537	969.5073	2
LFRKGLGKSKKIHSVSA	619.4378	1855.116	0
LFRVRLEV	516.387	1030.629	0
LFVLSRDGKINIVDGDSGK	678.4709	2032.095	0
LGGSGKTTLAKAVGK	694.4346	1386.82	2
LGGSLSYACAGGDC	425.1359	1272.512	0
LGILIALILIIVLSVCRGF	507.3267	2025.279	0
LGKFLKSH	465.3147	928.5494	2
LGKLRGIKFMS	625.4224	1248.738	2
LGLGGLPALLL	518.8878	1035.669	2
LGLKVSKPRNPVR	732.4769	1462.91	2
LGLPRTRDF	537.8771	1073.598	2
LGQHAKHQP	508.3344	1014.536	2
LGRALALELAH	582.3703	1162.682	2
LGSVQSLRRVLLWKN	590.4256	1768.047	0
LGVLCACS	383.1667	764.3561	0
LGVPAH	593.4013	592.3333	0
LGVPAH	593.4017	592.3333	0
LGYKTVLK	461.3294	920.5695	2
LGYLNWK	447.3156	892.4807	2
LGYLNWK	447.3156	892.4807	2
LHFQLKTFILKGV	772.5016	1542.929	2
LHGQLQRIH	551.391	1100.62	2
LHKGQAV	753.4732	752.4181	0
LHLVLALLALTAGSAG	760.4844	1518.913	2
LHNNLVLH	480.3055	958.5349	2
LHQKLGVLQKSLKGM	840.5411	1678.992	2
LHRFSHVIV	554.371	1106.635	2
LHRLAIRS	483.3757	964.593	2
LHSLAILSR	505.3889	1008.608	2
LHTGSWYK	496.3276	990.4923	2
LIAGSLIKNPGGTIAPCG	421.3188	1680.923	0
LIFFLFCMDYW	749.3179	1496.687	2
LIGHLKVF	463.8407	925.575	2
LIGKAPPRN	483.3243	964.5818	2
LIGVIVGVLAFLLILLFILTFYCMRKRN	810.5573	3237.927	0

LIHGQKF	421.7935	841.481	2
LIIFSFLLLLFVPI	550.0435	1647.042	3
LILRFFGLGFT	642.4225	1282.744	2
LILRIRLRDYGALSRQ	648.4651	1942.159	0
LILTLKWCL	551.8805	1101.662	2
LILVSH	681.4529	680.4221	0
LILVSH	681.4543	680.4221	0
LIQAEVSS	423.7214	845.4495	2
LIQAEVSS	423.7214	845.4495	2
LISAYVRNR	546.3722	1090.625	2
LISKALAKK	486.3769	970.6539	0
LISKALAKK	486.3783	970.6539	0
LISKFKF	441.8273	881.5375	2
LITKQGKQ	458.3033	914.5549	2
LITSRHSLVSLLLI	522.3449	1563.971	3
LIYGTGNR	447.3446	892.4767	0
LKAGPVVGFV	493.8508	985.5961	2
LKAHRLAR	964.7108	963.609	0
LKAQTTGY	441.2959	880.4655	0
LKEIHDKFLALMIALVAAKN	560.4129	2237.297	0
LKEIKKTKKRHQ	513.0022	1535.962	3
LKENLSAAGKGKPPLTQTRR	722.5035	2164.244	0
LKFREEHKKPLF	786.4774	1570.898	2
LKGVSGNNNSST	295.15	1176.574	0
LKHGKKSL	910.6645	909.576	0
LKIALAAPVVTVA	633.4298	1264.812	2
LKILNLSHSRN	647.9415	1293.752	2
LKKGNLVRF	537.8772	1073.671	2
LKKLKQIDEVRK	499.9795	1496.94	3
LKKVIAMRSRMEF	537.0205	1607.9	3
LKLKMIKCLVKKKPPK	632.4701	1894.235	0
LKMISSF	413.3208	824.4466	0
LKNLFRIRVINTG	772.5016	1542.936	2
LKQPQLFKR	579.4249	1156.708	2
LKSIKSSH	450.2958	898.5236	2
LKSPVWRG	471.8381	941.5447	2
LKSRPRPSSPSPGSALRRPNAAARNGDAVPGRVRVAV	955.7527	3819.132	0
LKVSPVVI	427.8124	853.5637	2
LKVYTTKYKNAS	708.4626	1414.782	2
LKYIVEKNSKR	689.4901	1376.814	2
LLACKHMKN	529.3767	1056.557	2
LLAVGTTTSQVRGCGEASCDSIF	772.3142	2314.093	3
LLDYKKRRWQK	767.4558	1532.894	0
LLEIILGKRSH	639.943	1277.782	2
LLESEAPK	443.8075	885.4808	2

LLFLSSKASFKPPKSIRGG	678.4773	2032.183	0
LLFRVIRVF	581.9031	1161.739	2
LLFSVNASGQ	518.3412	1034.54	2
LLHLKTSA	441.8127	881.5334	2
LLHLNVAAC	477.3167	952.5164	0
LLHRAAK	808.5812	807.5079	0
LLIAVPKK	441.3717	880.611	0
LLIHCQRWLRIN	522.346	1563.882	3
LLIRVF	760.5828	759.5007	0
LLIRVF	760.5815	759.5007	0
LLKLRRH	468.3877	934.6188	0
LLKNSPTLLPLP	653.4484	1304.807	2
LLLFFCSH	490.3563	978.4997	2
LLLLFRKLKIAKLVNLV	997.6941	1993.354	0
LLLNLKK	421.3162	840.5797	0
LLLVGVF	760.5829	759.4894	0
LLLWILLMALI	656.4254	1310.84	2
LLNKFLSC	469.3263	936.5103	0
LLPPILFKFF	617.9318	1233.753	2
LLPPQQHHQ	549.3747	1096.578	0
LLPPQQHHQ	549.3761	1096.578	0
LLSFRKFS	499.3707	996.5756	2
LLSLRKCGR	523.3817	1044.623	0
LLSLSLYKRLIIAIEVK	986.7143	1971.25	2
LLSLSLYKRLIIAIEVK	986.7143	1971.25	2
LLSLVGNLGGIGG	585.3512	1168.682	2
LLTGQVVAVKRLN	705.9837	1409.872	2
LLTIATTISAVRLIVN	425.3231	1697.045	0
LLTSLLIIRFYR	503.3056	1506.929	0
LLVDGFPELSMT	441.279	1320.664	3
LLVFLKSYRN	626.8999	1251.734	2
LLVGTPGRILDLAKK	797.468	1592.998	0
LLVLVDSIRPNK	456.288	1365.834	3
LLVLVMDL	458.3033	914.5511	2
LLVTRNPKLKMI	713.4772	1424.89	2
LLYFPFKF	537.8771	1073.595	2
LLYKVVDMA	526.3282	1050.578	2
LMKLLTYESVEEMVVKRVKKKAM	689.1537	2752.542	0
LMKMEENGCLPNEFTYNVF	760.3191	2278.011	3
LMLSLKFGIIPIVV	772.0238	1541.962	2
LMSGGNSVRGSALVGS	746.4576	1490.751	2
LNEPKPYPEPKLF	786.4819	1570.84	2
LNHKKKLKCK	620.4098	1238.765	2
LNKVREICKCVLIG	794.5641	1586.9	0
LNKVREICKCVLIG	794.564	1586.9	0

LNLFSVQLRLGLF	760.4857	1518.892	2
LNLGDLVGKP	513.3769	1024.592	0
LPAFFKHNNFS	441.2966	1320.662	0
LPEPGKR	796.5392	795.4603	0
LPFPRRVFA	551.893	1101.645	2
LPGGPIDGKILG	568.8917	1135.66	2
LPGIPKQ	752.5135	751.4592	0
LPGRKKWVK	371.3259	1110.703	0
LPHVNAVGS	447.3156	892.4767	2
LPIEQQKLFVDGLIR	590.4251	1768.025	0
LPKDLFF	440.2733	878.4902	2
LPKYPPSKEM	397.1802	1188.621	0
LPLGLIF	772.5824	771.4894	0
LPLPISDPPKN	595.9193	1189.671	2
LPLPISDPPKN	595.9194	1189.671	2
LPLPISDPPKN	595.9194	1189.671	2
LPLVIKVIGRSLSNRP	441.3502	1761.099	0
LPLVPPTT	419.3159	836.5007	2
LPMLEHGL	455.311	908.479	0
LPNELNKLFYL	455.311	1362.755	0
LPNGVSHPSLNC	413.2631	1236.592	0
LPNGVSHPSLNC	413.264	1236.592	0
LPNIIILLPVEK	681.4929	1360.869	2
LPNNAPAW	441.7656	881.4396	2
LPNNFGSK	438.7872	875.4501	2
LPNNGFTF	455.2231	908.4392	0
LPNNGFTF	455.3093	908.4392	0
LPNNGFTF	455.3129	908.4392	0
LPNNGFTF	455.3108	908.4392	0
LPNNGFTF	455.3105	908.4392	0
LPNNGFTF	455.312	908.4392	0
LPNNGFTF	455.3106	908.4392	0
LPPELGKLDKLVSLD	546.3976	1635.945	0
LPPSREEQWKMAVAELSHKLLHATRKRDEAL	910.6646	3638.947	0
LPRRAKSKKR	620.434	1238.805	0
LPRSSSS	367.1724	732.3766	0
LPRSSSS	367.171	732.3766	0
LPRSSSS	367.1708	732.3766	0
LPRSSSS	367.1715	732.3766	0
LPRSSSS	367.171	732.3766	0
LPSILVPNLKR	625.4258	1248.792	2
LPSMVTPL	429.3164	856.4728	0
LPTEKLLVLGG	570.3664	1138.696	2
LPVILTSLIHS	604.8872	1207.718	2
LPVVMVASYT	540.355	1078.573	2

LQAGVGLSALDFSVF	762.3227	1522.803	2
LQARHNKA	469.3269	936.5253	0
LQGDSAGNHLKVAACCKHYTAYDLDNWN	777.5677	3106.403	0
LQHQGERLEEM	693.3832	1384.641	2
LQIYKTRN	518.3415	1034.587	2
LQLNNGKKKKI	642.4225	1282.809	2
LQPLPPLARQAS	645.9706	1289.746	2
LQVLPTAIGVF	579.4249	1156.686	2
LRDKVKSSLR	601.4387	1200.73	2
LREKKRNN	529.3716	1056.615	0
LRIGMGC	383.1667	764.3673	2
LRIGMGC	383.1651	764.3673	0
LRIMGGC	383.1667	764.3673	2
LRIMGGC	383.1657	764.3673	0
LRIQRAQRRR	676.9483	1351.839	2
LRIVVVLLPSLLHLR	871.1207	1740.15	2
LRIYKERN	546.3992	1090.625	0
LRMRRIVIQEL	713.9824	1425.86	2
LRNPLISN	463.8407	925.5345	2
LRPNFS	367.1708	732.3918	0
LRPNFS	367.1709	732.3918	0
LRPNFS	367.1712	732.3918	0
LRPNFS	367.1711	732.3918	0
LRPNFS	367.1702	732.3918	0
LRPNFS	367.1706	732.3918	0
LRSSAAPKRR	571.4282	1140.684	2
LRTGLDRRLTAWK	529.3276	1584.921	3
LRTLPYIFH	580.3847	1158.655	2
LRTLYKHH	534.3657	1066.604	2
LSDGLMGFYERMGDTLAHQ	535.9075	2139.972	4
LSDLAPNK	429.3167	856.4655	0
LSDLAPNK	429.3162	856.4655	0
LSDLRAQVAANQ	429.3166	1284.679	0
LSEEVRAAHKREF	786.4257	1570.822	2
LSEHPAGVYFDWAGLSARGVF	760.319	2278.117	3
LSGYLKKFN	535.3994	1068.597	2
LSGYLKKFN	535.3994	1068.597	2
LSIPLAKEGAV	549.415	1096.649	2
LSIQQPLK	463.8407	925.5597	2
LSISLLIIKLH	625.4258	1248.817	2
LSKLVLRKDIKN	713.9822	1425.903	2
LSKPIPDGSSPDYERSTVEEALKVKSDRQ	808.5803	3230.642	0
LSKRKNAF	482.3006	962.5662	2
LSKVAVHK	441.3725	880.5494	0
LSLAIIAIVVAAVGG	683.9713	1365.86	2

LSLLIKKHQ	540.354	1078.686	2
LSLLIKKHQ	540.354	1078.686	2
LSLLIKKHQ	540.354	1078.686	2
LSLLIKKHQ	540.354	1078.686	2
LSLLIKKHQ	540.355	1078.686	2
LSLSNNSF	441.2074	880.4291	0
LSPSLSHFHQHSH	505.3489	1512.722	0
LSQLPLNPAPKELQ	782.5003	1562.867	2
LSRKISTRVF	603.9157	1205.725	2
LSRSTMPVLELQILTI	605.4382	1813.038	0
LSSHKQSLTT	551.3778	1100.583	0
LSSSNKSLAASAIEYTNPA	962.4972	1922.959	2
LSTAAAKAVKKLLPKS	813.5301	1625.024	0
LSTEAPNPGHGQGSNK	797.3939	1592.754	2
LSTIVIGFAYN	599.3674	1196.644	2
LSYLGMRN	477.3172	952.48	0
LTFPAHLTH	518.844	1035.55	2
LTGSSPFAGIAIGSETSGGVENVLAEHINLF	772.585	3086.556	0
LTKKGLIPNAF	601.4624	1200.723	0
LTKVFTSKTKKKKTK	442.3512	1765.119	0
LTLVEKDENNELMEIDH	681.3101	2040.967	3
LTREVFHGRLRN	499.9799	1496.832	3
LTRKRRQFSM	661.9582	1321.74	2
LTSAKVSLLDH	592.4264	1182.661	2
LTSDVIARSA	516.8352	1031.561	2
LTSGLI	302.1955	602.364	2
LTVVIHGS	413.2659	824.4756	0
LTYFIV	378.2116	754.4265	2
LTYIFVII	491.3318	980.5947	0
LVDSERGHVTTQVKGTMGYLDP	601.463	2402.19	0
LVGCLALIRR	557.4376	1112.685	0
LVIAVGISIFKIII	750.0113	1497.99	2
LVIAVGISIFKIII	750.0111	1497.99	2
LVITIALILVLFLF	794.5629	1587.042	0
LVKILTIFTCIDF	763.5146	1524.863	2
LVKILTIFTSIDL	738.4732	1474.901	2
LVKKKPPK	469.3493	936.6484	0
LVLFVVVVAHQ	612.4204	1222.744	2
LVLPVVF	786.597	785.5051	0
LVNGKNTIY	511.3148	1020.56	2
LVNNTASY	441.2663	880.4291	2
LVPKGMNIQIPVPLLN	873.5996	1745.027	2
LVPLGYRSA	496.3288	990.5498	2
LVPLGYRSA	496.3288	990.5498	2
LVPNIKILKQCG	663.4349	1324.79	2

LVPPQESQRR	403.8945	1208.663	3
LVRVEVKGCGKFG	696.4341	1390.776	2
LVTSTMKKRRNN	724.4485	1446.809	2
LVVANNSALSLLITVIGA	590.0622	1767.051	3
LVVHFDKPQL	598.3967	1194.676	2
LWRILGVN	485.8537	969.576	2
LYHPLFKRLLDK	772.024	1541.908	2
LYHVLLSLGS	551.3763	1100.623	2
LYIVKNLL	488.3311	974.6165	2
LYRIQKILM	393.2955	1176.705	0
LYRPVHSRSMGNMS	409.362	1633.782	0
LYYAVWFNSQSSQ	797.3401	1592.715	2
MARYVYGLIFLVAN	544.0082	1628.875	3
MEDTHIDELQSSSFRNTCN	557.4378	2225.932	0
MENSSEKCNFEGNKELNAS	533.4527	2129.899	0
MERREKGEK	581.9034	1161.592	2
MESRDLPSSSPSSTANRDASSATDAEDGVF	772.5813	3086.337	0
MFEM	279.1586	556.2025	2
MGIQKYVFYSIHNCD	455.221	1816.828	0
MGITHLQRIGTGLF	772.5015	1542.834	2
MGPMGQMGPMVQRGPMNQMGPMDHMRNMNINM	909.6477	3634.517	0
MGSKILLTTRN	617.4287	1232.691	2
MGTTTTPLSH	523.3564	1044.491	2
MHNVHQ	383.166	764.3388	0
MISFINSTNNNCLMMFDEAQETTTQLF	786.5969	3142.376	0
MITGRRAIDQSRPSEEQNLVTWAQPLF	786.5992	3142.598	0
MITGRRAIDQSRPSEEQNLVTWAQPLF	786.5974	3142.598	0
MKLHSATELPKADQNCKNVESSIEHP	727.4502	2905.406	0
MPLCLLDLQTESWMCTMGLNGFCRSLF	786.0979	3140.397	4
MPVPVCTC	425.2126	848.3594	0
MQLKQMC	441.2066	880.3969	0
MREIQAIMRQIASSITYLPCLDEPCVF	786.5979	3142.533	0
MRLPVRSVLGFKC	753.4737	1504.837	0
MSSSNFE	409.1614	816.296	0
MSSSNFE	409.1615	816.296	0
MTEPRAVRK	363.2135	1086.597	0
MVKEELVK	488.3573	974.5471	0
MVKEELVK	488.3574	974.5471	0
MVLLGLLLVGFLSQLGSSV	649.4465	1945.132	0
MYEF	295.128	588.2254	2
MYGNCKSM	467.1009	932.3554	2
MYKKRRAIK	597.3957	1192.723	2
MYMEEM	425.1341	848.2755	0
NAGVF	507.3288	506.2489	0
NAGYAIFVV	477.3163	952.5018	0

NALRSMFA	455.3089	908.4538	0
NASVNFLDEEKPLYPPTIKNDDLHKVF	786.5956	3142.597	0
NATVF	551.3575	550.2751	0
NCELLTNNQP	573.2201	1144.518	2
NCESDHICYDEHGDAC	453.3902	1809.603	0
NCSNLGGAGLPPDADSLSDKQHAGGTTASIPSS	782.1004	3124.437	4
NDFRNNSMQSEPTTYNGTS	541.4411	2161.897	0
NDFYSQGVLQAPRSFLFPCK	773.0081	2316.136	3
NDPNHWV	441.207	880.3828	0
NEAGTGTYKGPNNLPASFMTATESGDRYEP	794.5621	3174.42	0
NEFPSEGVDPGRSSSSRWTEMEISNLMQLR	860.6646	3438.593	0
NEGSKRVKN	516.3866	1030.552	0
NEGSKRVKN	516.3874	1030.552	0
NEGSKRVKN	516.3872	1030.552	0
NEVVLPMEYFSSNPSSKSK	536.4853	2142.03	0
NFGRYKQTYTRKRQK	494.3168	1973.071	4
NFIVLNDL	474.3152	946.5124	2
NFLKFWRNAT	648.9142	1295.678	2
NGDTLVVKVTN	580.4022	1158.625	2
NGFLKK	706.506	705.4174	0
NGFLKK	706.5064	705.4174	0
NGPKPVVAFVTG	593.4408	1184.655	2
NGPKPVVAFVTG	593.441	1184.655	2
NGRPDSGF	425.2117	848.3777	0
NHKKIFAAS	508.3682	1014.561	2
NHTGGGY	353.1551	704.2878	0
NIACHVAVFFLHFFLKGGCNGIGAWLFNSILNLAL	955.7521	3818.995	0
NIACHVAVFFLHFFLKGGCNGIGAWLFNSILNLAL	955.7546	3818.995	0
NIFLNKLKKS	602.9101	1203.734	2
NILKPGPQA	469.3508	936.5392	0
NIPVGGGSRKNKRSSASC	455.3111	1816.933	0
NIRSQWRHLTIF	786.4221	1570.837	2
NIVINNNDPSSSSVSYNNPSPRLA	640.5334	2558.236	0
NKGPPAK	711.4627	710.4075	0
NKGYYGRGPIQL	683.427	1364.72	0
NKHH	535.3589	534.2663	0
NKHH	535.3595	534.2663	0
NKHH	535.3595	534.2663	0
NKHH	535.3589	534.2663	0
NKKKVNF	439.2902	876.5181	2
NKKRKKGRRE	650.4207	1298.801	2
NKKRKLPDGISRGVELLKN	722.5025	2164.281	0
NKKRPRGLKA	584.3801	1166.736	2
NKKSLEVNLP	571.4282	1140.65	2
NKPRKSSNKPK	642.4225	1282.747	2

NKVLSVVG	815.5104	814.4913	0
NKYTGVLGVF	549.4149	1096.592	2
NLCELQRQCPSSDSICPYEVNYLSNGTSTTGFL	910.665	3638.633	0
NLCSKCYKDYLKVTKSNECETKNLNDQVFVL	910.6652	3638.778	0
NLEGLKFPKSL	623.4519	1244.713	2
NLESSSHN	444.3052	886.3781	2
NLIKAQEQLKKLKTE	595.3887	1783.057	3
NLKDSCRDTAQASEESGACDFTMAINDF	760.5803	3038.269	0
NLKYLILRN	573.9063	1145.692	2
NLKYLILRN	573.9063	1145.692	2
NLLRLALSNGYQ	341.2654	1360.746	0
NLNIAYKMSVREYQNLTPYATALEENW	808.58	3230.571	0
NLQKKVLSFVK	652.4234	1302.802	2
NLRLLDLH	497.3201	992.5767	2
NLTGEIPATLNAVPAL	399.3069	1592.877	0
NLTKLKVKS	515.8644	1029.655	2
NLVNRYKGKET	441.2967	1320.715	0
NMIVGGLKLHVN	647.9425	1293.723	2
NMKAKTRSEACQRKRSQAFDSNKVTSME	808.5816	3230.571	0
NMMFDVSNGDDYLIHQYVGP	772.3241	2314.003	3
NNGGNAIYMS	529.197	1056.418	2
NNGVLPLKPPPGRPDLLPHE	540.8618	2159.185	4
NNHNYA	367.1703	732.2827	0
NNHNYA	367.1682	732.2827	0
NNHNYA	367.1707	732.2827	0
NNKKNTM	425.2119	848.4174	0
NNKKNTM	425.2136	848.4174	0
NNKSKLSG	424.2731	846.4559	2
NNNDDNDNNNNDPNNN	458.3471	1829.643	0
NNNDDNDNNNNDPNNN	458.3469	1829.643	0
NNNRRHNPNVLTFRDFVNHVHNTRRGGDC	865.6208	3458.674	0
NNPAGSPH	397.1816	792.3514	0
NNPFSFLVPPQESQRR	639.331	1914.97	3
NNPFSFLVPPQESQRR	639.331	1914.97	3
NNVLYILKVM	603.9157	1205.684	2
NNVRVKDTFSCRAWGYVSNDYRPREFFLSL	910.6629	3638.784	0
NPANIHQ	397.1809	792.3879	0
NPANIHQ	397.1804	792.3879	0
NPFSFLVPPKESQRR	601.3165	1800.964	3
NPFSFLVPPQESQRR	601.3165	1800.927	3
NPIKRK	755.4882	754.4814	0
NPSSC	254.1608	506.1795	2
NPVCHFLGSK	551.3547	1100.544	0
NQENGAISFFWSWSYWSTF	786.337	2356.023	3
NQFKTKGGLKVGT	689.4928	1376.778	2

NRIPIV	711.4609	710.4439	0
NRKKNKHNNLS	676.9483	1351.743	2
NRKN	531.3848	530.2925	0
NRKSLPV	813.5289	812.4868	0
NRLVKKSEK	551.3509	1100.667	0
NRNKTH	385.1797	768.3991	0
NRNNKKGNHKVLK	517.3678	1548.896	0
NRRVHVILN	560.8615	1119.663	2
NRSWQLRK	544.413	1086.605	2
NRWRKR	458.3033	914.5311	2
NRYLTAWK	526.3363	1050.561	2
NRYLTAWK	526.3363	1050.561	2
NSMVWMVLAMIF	481.2378	1440.697	0
NSNNNNDNLNNPQRPTSSGMS	569.4718	2273.968	0
NSSELVLWN	531.2719	1060.519	0
NSSTGLVVPTIDL	658.408	1314.703	2
NSVTVKR	402.2588	802.4661	2
NTLNQGF	397.1802	792.3766	0
NVDDINNYADLGFYSQIS	683.3071	2046.917	3
NVHDVPSQGAKRTSYSGNLT	533.4504	2130.045	0
NVIEETASM	497.2327	992.4485	0
NVINHER	441.2663	880.4515	2
NVIPVLA	725.4797	724.4483	0
NVLNKISSGKEVSF	761.483	1520.82	2
NVNQPRHFCKNCQRYWTAGGVMRN	720.525	2878.344	0
NVSGLNNALKPLLRWAKK	1011.67	2021.19	0
NVTNNGF	383.1674	764.3453	2
NVTVLEKPVPLL	441.2802	1320.802	3
NVVVRCSVSSS	568.878	1135.566	2
NVYVNSTFDGPTFSYGSGNQNLTLFYECEASSRITETPENLF	786.172	4711.134	6
NWDTGHVISEAGIF	773.3507	1544.726	2
NWVSRNVV	487.3246	972.5141	2
NYCPGDKSCPHQSS	381.3314	1521.598	0
NYLRKRYREDLFKD	639.4132	1915.007	3
NYSSS	279.093	556.2129	2
PAAPPPQKKK	531.3836	1060.639	0
PAIAVALGV	810.5554	809.5011	0
PALASRIIQGNK	423.3618	1266.741	0
PAPGPGGPKHHFNGERQVKDFIHTLLHYGG	816.6364	3262.654	0
PARSDST	367.1707	732.3402	0
PASHVTRKKK	576.4109	1150.694	2
PASKAQVVGWPPIRSFRKNSMATTT	683.4297	2729.444	0
PAVLAYLY	455.3104	908.5007	0
PAVPDPKTALMFYALLLKFGADYANDVF	772.5826	3086.583	0
PCAHN	271.0604	540.2115	2

PCAHN	271.0602	540.2115	2
PCAHN	271.0602	540.2115	2
PDDSPALD	415.2109	828.3501	2
**PDIGGFGC**[d]	383.1668	764.3163	0
**PDIGGFGC**[d]	383.1661	764.3163	0
**PDIGGFGC**[d]	383.165	764.3163	0
**PDIGGFGC**[d]	383.1653	764.3163	0
**PDIGGFGC**[d]	383.1654	764.3163	0
**PDIGGFGC**[d]	383.1656	764.3163	0
**PDIGGFGC**[d]	383.1672	764.3163	0
**PDIGGFGC**[d]	383.1672	764.3163	0
**PDIGGFGC**[d]	383.1658	764.3163	0
**PDIGGFGC**[d]	383.1653	764.3163	0
**PDIGGFGC**[d]	383.1666	764.3163	0
**PDIGGFGC**[d]	383.1659	764.3163	0
**PDIGGFGC**[d]	383.1651	764.3163	0
**PDIGGFGC**[d]	383.1658	764.3163	0
**PDIGGFGC**[d]	383.1657	764.3163	0
**PDIGGFGC**[d]	383.1657	764.3163	0
PEEVQQGF	467.2041	932.424	0
PEFDVRN	438.787	875.4138	2
PEGVNNIHL	497.2312	992.4927	0
PEHFCECYSD	615.1371	1228.417	0
PEIQGRFIGTKLHTLKADFYEVAKMF	760.5829	3038.605	0
PEPFIDERVERYGSIFTTHVFGEATVF	786.5981	3142.54	0
PEQRIIAVKHLGI	737.4985	1472.883	0
PESESHPHAQPTIEQQQ	486.3774	1941.882	0
PFDSDSIQFKLDDNFTAGNNN	590.4246	2358.04	0
PFDSGRDGFVIGEGSGVLVLEE	760.3191	2278.112	3
PFIIAAHRQLSAM	727.998	1453.786	2
PFPPSLLLMPSINYLDV	639.413	1915.017	3
PFSFLVPPQESQRR	563.3025	1686.884	3
PFSFLVPPQESQRR	563.3025	1686.884	3
PFSFLVPPQESQRR	563.3014	1686.884	3
PFSIGLSCDGSPICEGSDGVLFPKGQPIPSVK	808.5804	3230.599	0
PGHHGADTYIVQLPKDQVYRVP	623.4104	2489.282	0
PGKKGH	623.4095	622.3551	0
PGNFPSSPF	475.2192	948.4341	2
PGNNYSGG	383.1672	764.309	0
PGNNYSGG	383.1671	764.309	0
PGNNYSGG	383.1652	764.309	0
PGNNYSGG	383.1653	764.309	0
PGQEDPPLTWALRLKI	612.0539	1833.015	3
PGRNRHGA	440.7751	879.4423	2
PGVFTELIQ	502.3713	1002.539	0

PGVKGCYVATGHNCWGILNGPAT	772.3217	2314.099	3
PHALALVSSFGI	606.3938	1210.671	2
PHKFKLNFMGS	441.279	1320.665	3
PHKKPRGK	474.3152	946.5825	2
PHNKAFNTTSP	405.2602	1212.589	0
PHNYGET	409.1613	816.3402	0
PHSHVPPQPYPQEWGNPNWPPN	644.4922	2574.183	0
PHSHVPPQPYPQEWGNPNWPPN	644.494	2574.183	0
PHTFVIISLDPPI	483.6518	1447.808	3
PIAVKRLSRTSK	678.4756	1354.841	0
PIGCDGSEKDEFAD	371.3255	1481.598	0
PIGCDGSEKDEFAD	371.3249	1481.598	0
PIGGIEAPK	441.2999	880.5018	2
PIIIPR	708.4887	707.4694	0
PILPSLLSS	463.8406	925.5485	2
PIMHRAVTSSNIL	360.3623	1437.776	0
PINRKPHRSG	581.3605	1160.653	0
PINTPKQQKR	605.4205	1208.699	0
PIRKALNRNFIVVGGYN	644.4693	1930.09	3
PIRKQLNSSLFSF	513.0035	1535.846	3
PIVYPPVVPKPPIVT	808.5789	1614.975	0
PKAIPSRLLLTGACF	794.0362	1585.901	2
PKDLWERVKL	642.4225	1282.74	2
PKDSIVSKVCELKNKK	606.1053	1815.029	3
PKFKAIPLNKKIF	772.5015	1542.965	2
PKKETAMKTYEVF	786.4093	1570.807	2
PKKETAMKTYEVF	786.4079	1570.807	2
PKLKVFHCFHMSDEFKESF	786.0155	2355.118	3
PKLRLLAVLYSG	665.4701	1328.818	2
PKRIYRNN	530.8498	1059.594	2
PKRYFLYC	545.3703	1088.548	2
PKSKPAKVAAKKVAAKPAK	959.6294	1917.225	2
PKVDIPSCFVGLVLENCE	981.4646	1960.964	2
PKVESWSSEFSMIDWVKIF	772.3241	2314.134	3
PKVPELSKPDMS	664.4361	1326.685	2
PKVSKVLVKTEASNNSL	605.4189	1813.031	0
PLAVPKVKPTLTF	705.9836	1409.865	2
PLCIRHGS	441.8273	881.4542	2
PLCSQAF	383.166	764.3527	0
PLDFSHSLLPAFFKHNNFS	555.2731	2217.101	0
PLGQQSMAVLRKPE	523.9974	1568.835	3
PLIQSKENPNEF	708.4612	1414.709	2
PLITCVFG	425.2091	848.4466	0
PLKALPLW	469.3271	936.5797	0
PLKRIKYVNQSRN	808.5805	1614.932	0

PLLLQRRPSK	604.4391	1206.756	0
PLLPISDPPKN	595.9193	1189.671	2
PLLPKLHYIHP	664.4361	1326.781	2
PLLVTLIIRNN	633.4252	1264.787	2
PLNKSFTC	455.3122	908.4426	0
PLNMFPQETISS	455.3122	1362.649	0
PLPLSSKHH	508.3344	1014.561	2
PLPVVLQDNRN	632.9174	1263.694	2
PLQAPQNIQVASGVAARPG	469.3266	1873.017	0
PLQPSPPSS	455.3112	908.4603	0
PLRKQISGATTGV	664.4361	1326.762	2
PLSFSATYNSC	397.1811	1188.512	0
PLSKGLLVFGLLLVF	808.5802	1615.011	0
PLVLYLIASH	563.3702	1124.659	2
PNAWNTHQCNKNKSLTLTLPQNHGGSD	744.5803	2974.41	0
PNFELNKN	488.3311	974.4821	2
PNFESILR	488.3311	974.5186	2
PNGDDEVSMTM	399.1389	1194.453	0
PNLHTKETLA	562.3672	1122.603	2
PNLSKLLPAK	540.9018	1079.67	2
PNREMTVF	497.3217	992.475	2
PNVIGGKPRILLLTGSPPKP	685.4319	2053.241	0
PNVVFQSSKVHK	685.4283	1368.751	2
PNWREIHR	554.3709	1106.573	2
PPCAKFVVAKYG	427.2796	1278.679	0
PPDGNSY	383.1649	764.2977	0
PPGGVWQGDTTQDGFACPDYGF	772.3166	2313.964	3
PPGMHKD	399.1414	796.3538	0
PPGMHKV	383.1663	764.4003	0
PPIIIPPRPTRPT	727.998	1453.877	2
PPKCTKSS	424.2705	846.4269	2
PPKECCG	367.1715	732.2935	0
PPKIPNMS	442.2947	882.4633	2
PPKK	469.3496	468.306	0
PPKKVIIKSAD	598.3967	1194.734	2
PPKPPPQTTA	517.3689	1032.56	0
PPLMRFLRN	572.4127	1142.638	2
PPLPPHQSH	505.3491	1008.514	0
PPNFETKSKAPPTEEVSE	497.4152	1985.958	0
PPNFETKSKAPPTEEVSE	497.416	1985.958	0
PPNGKKNWG	499.2258	996.5141	2
PPNGQSDQNALTHS	367.1709	1464.659	0
PPNKKKRN	491.3723	980.588	2
PPNKKVVLV	497.3306	992.6382	2
PPNKKVVLV	497.3335	992.6382	2

PPNKVNTI	441.8273	881.4971	2
**PPNNNPASPSFSSSS**[d]	497.2326	1488.648	0
**PPNNNPASPSFSSSS**[d]	497.2341	1488.648	0
**PPNNNPASPSFSSSS**[d]	497.2299	1488.648	0
**PPNNNPASPSFSSSS**[d]	497.2332	1488.648	0
**PPNNNPASPSFSSSS**[d]	497.2327	1488.648	0
**PPNNNPASPSFSSSS**[d]	497.2337	1488.648	0
**PPNNNPASPSFSSSS**[d]	497.2337	1488.648	0
**PPNNNPASPSFSSSS**[d]	497.2328	1488.648	0
**PPNNNPASPSFSSSS**[d]	497.2313	1488.648	0
PPNPTSLFGRMT	439.8961	1316.655	3
PPNQDTPTSPTAFT	497.2329	1488.673	0
PPNRKRPKCSAEVEQKQNN	556.4412	2222.134	0
PPNRYACLVAETAIDDPDS	512.414	2045.936	0
PPNSFHHK	482.3031	962.4722	2
PPNSGNVVVAPP	383.1674	1146.603	0
PPNTSFGKRRK	644.4143	1286.721	2
PPNTSFGKRRK	644.4156	1286.721	2
PPNVFKPPN	505.3889	1008.539	2
PPNWAQASDN	367.1708	1098.473	0
PPPHSS	311.1871	620.2918	2
PPPPNLEEI	503.3355	1004.518	2
PPPPPPAAAGGPPPHP	727.4573	1452.751	0
PPPPSPDSNAVSKSTTPNAS	492.4591	1965.928	0
PPPSPSTNNCPPPPSP	397.1791	1584.724	0
PPPSQSLQNSS	381.1859	1140.541	0
PPQIFQHELLQNFS	425.2116	1696.857	0
PPQKVA	639.4075	638.3752	0
PPQNDKEET	353.1562	1056.472	0
PPQNILMMPTISDVSMVEE	533.4517	2130.005	0
PPQQQQQQPPQSDDCS	453.3892	1809.759	0
PPQQQQQQPPQSDDCS	453.3881	1809.759	0
PPQSTPGRQSS	381.1851	1140.552	0
PPQSTPGRQSS	381.1861	1140.552	0
PPQSTPGRQSS	381.1861	1140.552	0
PPQSTPGRQSS	381.1857	1140.552	0
PPQVGKKLVH	551.893	1101.666	2
PPQWEFSCDMEMYFGSE	525.4462	2097.78	0
PPRANSSD	422.2924	842.3882	2
PPRKKKK	441.3724	880.597	0
PPRNGI	653.3973	652.3657	0
PPRPDWYEEFYASVMNSATKDYEAE	749.5345	2994.302	0
PPRQQNSTGPGCLEGCLAALC	533.4518	2129.966	0
PPRRSVRRRLVQSTLFPH	551.3509	2201.277	0
PPRSFLPRYILSKLP	595.3887	1783.051	3

PPRVGPTSGCFSC	425.212	1272.592	0
PPSASGLPSA	442.2947	882.4447	2
PPSDMTFAYSDPDRSPYYN	556.4403	2221.926	0
PPSKFLKIAL	557.4118	1112.696	2
PPSPLALPIIGH	606.3935	1210.707	2
PPSSPSFGAS	467.305	932.424	2
PPTFK	295.1898	588.3271	2
PPVGRSA	683.433	682.3762	0
PPVYN	295.1898	588.2908	2
PPYFNDKNMASDKQYL	483.3997	1929.893	0
PQAASALKK	913.5153	912.5392	0
PQGKIVSKVYLCA	469.3268	1404.78	0
PQGNCYNPDTVVAHA	397.1801	1584.699	0
PQMTEGLKNWVAPTREFSTHQ	615.1365	2456.191	0
PRAGRKGRIS	549.4149	1096.658	2
PRERKKHE	540.354	1078.6	2
PRIRLVRNR	590.423	1178.747	0
PRLLVLLILFVS	691.9681	1381.906	2
PRPIVGIHRIVFVLF	588.4114	1762.077	3
PRRVPKKAPK	588.8909	1175.762	2
PRYKRN	417.2957	832.4668	0
PRYKRN	417.2968	832.4668	0
PSALDLKRSS	537.4109	1072.588	0
PSAPNYG	353.1553	704.3129	0
PSAPNYG	353.1556	704.3129	0
PSAPNYGNN	467.2048	932.3988	0
PSDDQSPQLTYNPQLDHGGWSRRGREF	786.5978	3142.46	0
PSEDTKTENQDTSVSEKRSDSDESQQKSDSD	865.6203	3458.468	0
PSGAAGWHV	441.2091	880.4191	0
PSGNFMMNSMESFGGYGG	468.3885	1869.701	0
PSHPPRRPS	515.8631	1029.547	2
PSLPKLHSH	508.3344	1014.561	2
PSNMINNAKNFAQPIMTVDDAPICGVLPQSN	825.5536	3298.578	0
PSSHN	271.0604	540.2292	2
PSSKAIMAEKIFNLAAQKLGFEFVTKKF	786.5971	3142.725	0
PSSVDPDDGSGEHNLSENYGTC	760.584	2278.892	0
PSVLLLVYMALAFGS	527.6783	1579.868	3
PTDILAHIPC	540.3442	1078.548	2
PTGSHNSSPPSHGHGSSPPSH	683.3071	2046.889	3
PTGSHNSSPPSHGHGSSPPSH	683.3071	2046.889	3
PTKKFKLPIKFL	730.4748	1458.933	2
PTKLHELILDINNNNAL	966.5973	1931.048	2
PTKLHELILDINNNNAL	966.5973	1931.048	2
PTKNAKKVVL	549.415	1096.697	2
PTKRKRVP	491.3724	980.6243	2

PTKRKRVP	491.3723	980.6243	2
PTKRYAGNL	510.345	1018.556	2
PTPPPPIGFGG	518.8879	1035.539	2
PTRLLENKSSSFAQ	789.4372	1576.821	0
PTSLKQFSH	522.8693	1043.54	2
PTVDQLF	819.4501	818.4174	0
PVATKILSIPLRRR	810.557	1619.036	0
PVDSGF	311.1454	620.2806	0
PVDSGF	311.1444	620.2806	0
PVDSGF	311.1442	620.2806	2
PVDSGF	311.1442	620.2806	2
PVDSGF	311.1452	620.2806	2
PVDSGF	311.1452	620.2806	2
PVDSGF	311.1428	620.2806	0
PVDSGF	311.1451	620.2806	0
PVDSGF	311.144	620.2806	0
PVEGPECGNMD	383.1659	1146.432	0
PVERLTFEEFFNHP	441.2075	1760.852	0
PVGLLDVVPQRLNWR	441.2961	1761.005	0
PVIGKIGPYTVF	645.9706	1289.738	2
PVIKNKLAIGSETK	499.9795	1496.893	3
PVILVVNK	441.3714	880.5746	0
PVKLSIGILPKN	639.9449	1277.807	2
PVKVNSLLVFTF	455.3098	1362.791	0
PVLTIPQN	441.3113	880.5018	2
PVNCPLSAM	311.1455	930.4303	0
PVNFEYI	441.2073	880.433	0
PVNFEYI	441.2068	880.433	0
PVNFEYI	441.2061	880.433	0
PVNFEYI	441.2062	880.433	0
PVNFEYI	441.2947	880.433	2
PVNMALFVWIICVAVS	441.297	1760.936	0
PVNMLNVP	442.2947	882.4633	2
PVNMVNHA	441.3091	880.4225	2
PVNMVNHA	441.3157	880.4225	2
PVNNGEMS	424.2741	846.3542	2
PVNNGEMS	424.273	846.3542	2
PVNNGEMS	424.2746	846.3542	2
PVNNGEMS	424.2741	846.3542	2
PVNQTSFS	440.2719	878.4134	2
PVNTLNPQ	441.7656	881.4607	2
PVNTNFAS	425.2125	848.4028	0
PVPVPKRRRSS	639.9449	1277.768	2
PVQNLGSCCFLQSGNLDYSSS	555.4558	2217.967	0
PVQNLLTP	441.3091	880.5018	2

PVQSVSSPDAKRV	685.4325	1368.736	0
PVRCFTCG	441.7656	881.3888	2
PVRCS	561.3957	560.274	0
PVREEDDDDDKSEVYSWFYMGMGIGF	772.5829	3086.295	0
PVRFSWIKRCTELIFRN	722.5042	2164.173	0
PVRIPSTAGPVLY	457.2705	1368.777	0
PVRQHCQEL	555.2743	1108.545	0
PVSKSRLQLLGVSS	735.9951	1469.857	2
PVVERLYFH	580.4083	1158.619	2
PVVTKEGGGEGEEGNK	397.3808	1585.758	0
PVWNFC	383.1672	764.3316	0
PVWSCSS	383.1656	764.3163	0
PWPASGF	381.1863	760.3544	0
PWSEELKLVSHG	691.4706	1380.704	2
PYKLKLNKFA	611.4013	1220.728	2
QAAAKLKAG	857.5595	856.5131	0
QACASPTAVKIVIPAG	746.4618	1490.846	2
QAFVGKKPHL	562.8704	1123.65	2
QAFVVGATSHCST	430.9042	1289.571	3
QAICMSEYRKPELYYDLLRRKNSSNGMSF	875.6532	3498.685	0
QAIQFFLGDIQQTKIHLFFSLSHTHTFPIAN	904.6905	3614.867	0
QAKKK	293.2102	584.3646	2
QALPAPPPGHMAIRS	772.0238	1541.814	2
QALSSHVWRHVSH	772.5028	1542.78	2
QAMLNAGLLPNVP	669.4155	1336.717	2
QAPSGYGS	383.1666	764.2977	0
QAVALRVG	813.5287	812.4868	0
QDESSETSSTSLFFTTSLGSVF	786.3396	2356.059	3
QDMRNGFYEKLESELQGS	533.4515	2129.969	0
QEGEQPLVHDGP	441.2067	1320.595	0
QEGEQPLVHDGP	441.2067	1320.595	0
QEGEQPLVHDGP	441.2077	1320.595	0
QEGGSTSPIQPRKCRTNRRRHGNDFQSEILPSLASL	1009.748	4035.057	0
QEKLREQVSSSESSEASQQQYITIRQ	760.5815	3038.491	0
QENGKPGAELHLKV	760.4857	1518.815	2
QFGCLKTYNTF	441.2058	1320.617	0
QFPERYF	493.8504	985.4658	2
QFPERYF	493.8508	985.4658	2
QFVIQKVN	488.3311	974.5549	2
QGAHDRSRDEVTKVFDQQHFG	615.1382	2456.158	0
QGCRASDACRLYFQSVGDANNSVKPSKCF	788.6054	3150.443	0
QGDIGVSKSTDKGATW	413.2628	1648.806	0
QGHITPMLKLAK	668.9478	1335.77	2
QGKKTPVVAKLETK	772.0228	1541.914	2
QGLAAFTSS	441.2973	880.4291	0

QGLAAFTSS	441.2999	880.4291	2
QGLAAFTSS	441.2962	880.4291	0
QGPRTYLSPACHTMLTNEKRSFCHCLRN	816.6397	3262.537	0
QGYMTSTTETWELSSLAH	681.3088	2040.91	3
QHGVDTNSVNNRDNGELINIGNHLNFNDASNDM	910.6647	3638.604	0
QHTNYA	367.1706	732.3191	0
QIGVALYLLYTQVK	537.0204	1607.929	3
QIIVMRM	437.2351	872.4612	0
QISISRSS	439.2769	876.4665	2
QITTNSS	367.1714	732.329	0
QIVKLGGRILEI	669.9557	1337.84	2
QKHPTFSH	491.3338	980.4828	0
QKQEAMWERQHELAADKIF	786.6649	2357.159	3
QKQECSVDSLSPNHGNELKNM	786.337	2356.043	3
QKVAKMFKN	538.8488	1075.585	2
QLAAKVAEFLR	623.4512	1244.724	2
QLGDEVRKKEGK	685.4283	1368.736	2
QLKVHILYTNV	664.4351	1326.766	2
QLPKLPF	421.7935	841.5062	2
QLPNCC	339.1393	676.2673	0
QLRLHEVAIAELNNQPS	966.5973	1931.023	2
QLWFLKRFFRKKKL	969.6662	1937.188	0
QMFIVNAGSGFKLLWNTAKGFLDPMTTAKIHVLGN	955.7546	3819.001	0
QMKKKRVN	516.3864	1030.607	0
QNELGFVSG	467.2031	932.424	0
QNKHRGTASTL	606.8957	1211.637	2
QNLLLGFSFL	576.3834	1150.639	2
QNRPVSLTFK	595.3785	1188.662	0
QPCALGDNLPH	383.165	1146.513	0
QPGQNTG	351.1754	700.314	0
QPGVKKSVRFAKNRN	571.3785	1710.964	3
QPHALPQATRATPR	772.4838	1542.838	2
QPHLQGC	383.1648	764.3276	0
QPIKPNNAHQNSA	351.1748	1400.68	0
QPKVP	551.3546	550.3115	0
QPLLVLGPPFMFPTFEAQNLHNYRFL	773.1239	3088.6	4
QPPNPVATLQLCIGHN	568.0233	1700.867	3
QPRKVHK	438.2853	874.5137	2
QPSVKAALLAKLR	689.4901	1376.85	2
QQHHKLIRSHLF	772.5015	1542.853	2
QQKCGNVIIPFPF	369.1832	1472.749	4
QQSFVVAILLGVFV	760.4921	1518.881	2
QQWQKPA	443.3009	884.4504	0
QRKSKQKKKLKN	758.0082	1513.942	2
QRQEQH	413.2633	824.3889	0

QRTLHGLQVGESNKAKQHE	540.8618	2159.12	4
QSPEQSLSVSSSDSSSSCSTNSTS	595.4483	2377.966	0
QSTFDSIKCYLWKEECECLKWLDSQELNLVL	941.6965	3762.798	0
QSVVLETR	466.3185	930.5134	2
QTEQGVKYLLSYL	771.4802	1540.814	0
QTHHSSFYTNGGIDWHPNLRFEQNLVTRGDL	910.666	3638.74	0
QTHRFRKRKLLF	815.5097	1628.974	0
QTKKKRNLPGTP	684.4717	1366.804	2
QTLHAHLLKIGFHS	801.4944	1600.884	0
QTVLDFE	426.1975	850.4072	2
QTVSSSSTS	295.1498	882.3931	0
QVAKGRHAPCSGNNGTKSSFRN	766.9974	2298.104	3
QVGVPSLV	399.7835	797.4647	2
QVVAVAVFLALGF	667.4779	1332.781	2
QVVAVEFDS	497.2374	992.4815	2
QVYDPCTVLTVGVF	762.3184	1522.738	2
QVYDPCTVLTVGVF	762.3227	1522.738	2
QWFQSIPVN	559.8896	1117.556	2
QWIVRKRKR	635.4152	1268.794	2
QWQLSALGEIALV	714.4522	1426.782	2
RAATPSLLICAESQNGTDTTSAHYNFDLF	786.5955	3142.467	0
RAFATGRKF	527.4024	1052.588	2
RASDITENNNTS	441.2061	1320.591	0
RCDPNWEKNRKRLADCALGFGHGTTGGK	772.5842	3086.504	0
RCRLRLLK	529.371	1056.67	0
RDAAAALSTK	502.3734	1002.546	0
REKGPPMK	471.8371	941.5117	2
REVTGASHVF	551.893	1101.557	2
RFDGSQK	419.2513	836.4141	2
RFDGSQK	419.3142	836.4141	0
RFDGSQK	419.2497	836.4141	2
RFDGSQK	419.2513	836.4141	2
RFDGSQK	419.2513	836.4141	2
RFDGSQK	419.2499	836.4141	2
RFGRITKHKL	628.4067	1254.767	2
RFHHFRDGFC	441.2087	1320.593	0
RFHHFRDGFC	441.2097	1320.593	0
RFKAS	608.3858	607.3442	0
RFKEMVASK	548.3518	1094.591	2
RFKSKFPK	519.3438	1036.618	2
RFSFIGK	854.5789	853.481	0
RGFRFLCQAET	664.4351	1326.65	2
RGGKLMKLKSVLK	729.4785	1456.928	2
RGLSSLYKEIIDTVRKEA	520.3332	2077.153	0
RGPRSPSWKCSDVF	811.4166	1620.783	2

RGSCSSLSAN	491.2396	980.4346	2
RGVQQNQQGSPGLSWWSPTTAKSGGEQDERGRSSP	939.7074	3754.78	0
RGYKKLMN	505.3503	1008.554	2
RGYKRKSI	504.3285	1006.604	2
RHFDSYG	441.2053	880.3828	0
RHFDSYG	441.2056	880.3828	0
RHLELPSDSSLLFNLDDVADESLRKN	746.5642	2982.505	0
RIATVKNAHA	540.9015	1079.62	2
RIGTDAHL	441.8273	881.4719	2
RIHEKAAPKKPS	681.4926	1360.794	2
RIPEIKSH	490.3563	978.561	2
RIPNFS	367.1715	732.3918	0
RIRGGIVVIFKK	693.459	1384.903	2
RIRKLADNCT	595.3817	1188.64	0
RIVLIKIRN	562.9143	1123.755	2
RIWNKLRLCK	665.4701	1328.786	2
RKERKGDMI	378.2113	1131.618	0
RKKKERVKKK	664.4619	1326.894	0
RKKKKRSSSSKSRS	825.551	1648.981	0
RKKLPFCVGLLLRLLRKNLRVFVTCTSG	808.5802	3229.915	0
RKLRILCLHGFRQNASSFKGRTASLAKK	797.4668	3185.82	0
RKLRILCLHGFRQNASSFKGRTASLAKK	797.4684	3185.82	0
RKRSVVH	441.2956	880.5355	0
RKTKHVKKNR	647.9425	1293.811	2
RKYRTKKK	554.3709	1106.704	2
RLARPDAVFKIVH	761.483	1520.894	2
RLHQQIYKLE	664.4351	1326.741	2
RLLASKSCNHISM	487.3219	1458.744	0
RLLLRRLKSLN	461.3217	1380.904	0
RLPAVIGGSC	486.8235	971.5222	2
RLPNGELPSGFLGYAVTM	481.2364	1920.977	0
RLPNNSF	424.2717	846.4348	2
RLPSSSS	367.171	732.3766	0
RLPVKSLLQFKTVCKSWLSH	593.4015	2369.341	0
RLQTLITLGNYLFG	537.0205	1607.904	3
RLQTVYF	463.8406	925.5021	2
RLSGHQK	413.2555	824.4617	2
RLTSWKSWDDPSSGDIVWEVVI	644.4945	2574.275	0
RLVKRVIKHQEVL	809.5364	1617.02	0
RNARGIIIAGHRGG	483.3206	1446.828	3
RNEQQCSFVEHRN	412.3403	1645.738	0
RNEQQCSFVEHRN	412.3399	1645.738	0
RNEQQCSFVEHRN	412.3402	1645.738	0
RNEQQCSFVEHRN	412.3411	1645.738	0
RNGKSRIVSKEVF	760.4836	1518.863	2

RNGLAPKK	442.351	882.5399	0
RNIQQSM	438.787	875.4283	2
RNRLMRK	487.329	972.5764	2
RNTAFI	721.4831	720.3918	0
RNTTLTFSRRN	455.9526	1364.727	3
RPAKVLGKTSIN	642.4225	1282.772	2
RPFKPLLNAIS	628.4067	1254.745	2
RPGPPLKIIPF	617.9318	1233.76	2
RPHPYTKRKLGCN	523.9974	1568.836	3
RPHVAVH	815.5113	814.4562	0
RPINCM	367.1697	732.3411	0
RPINCM	367.1705	732.3411	0
RPINCM	367.1708	732.3411	0
RPKKKGYRRHT	713.9824	1425.843	2
RPKNNKVPVGPRL	738.4732	1474.873	2
RPRVPIITFI	606.3935	1210.755	2
RQKSSLENTEHNMNEVRTGS	773.0081	2316.088	3
RRALRALKGVVRIQALVRG	711.4614	2131.366	0
RRDILIGLGG	535.3994	1068.64	2
RRDRRAVVN	571.4282	1140.659	2
RRFSKMIR	547.3317	1092.634	0
RRKGLIKKA	535.3997	1068.724	2
RRKGSNK	423.3621	844.4991	0
RRKHKVF	485.854	969.5984	2
RRKPRVCKNKEEAETQRITHITVERNRR	876.6541	3502.924	0
RRKRRKGLK	599.3921	1196.805	0
RRKSEN	789.4375	788.4253	0
RRKVVKLV	997.6912	996.692	0
RRLASIFM	497.2996	992.559	2
RRLRPSRR	548.8989	1095.685	2
RRNFKGNKK	574.4278	1146.674	0
RRRVGLTGFK	595.3771	1188.72	0
RSFLKRGIYIAN	479.9712	1436.825	3
RSSLNVVAAEKGVVVGR	581.0477	1740.001	3
RSSTKPHNHKNGC	367.1708	1464.7	0
RSVKVLPVSRGDG	685.4283	1368.784	2
RSYYNNEEGGFA	352.3559	1405.59	0
RTPRHGKHSRNNNPFHLVPEEG	645.49	2578.301	0
RTPRKKMTKQ	637.4669	1272.745	2
RTSLFV	722.5027	721.4123	0
RVKLGFKIF	554.371	1106.696	2
RVLPLRRLH	580.4082	1158.746	2
RVLPLRRLH	580.3847	1158.746	2
RVRLKPGF	486.823	971.6028	2
RYIW	319.2236	636.3384	2

RYKGKTSQ	484.2976	966.5247	2
SAFAKPVQRRRKKGLDFRKW	825.5524	2473.429	0
SAFGKTGEKG	491.3311	980.4927	0
SAKGYKMKT	507.3645	1012.538	2
SAKPRLSPKP	540.9015	1079.645	2
SAQILAKVQAAAKNNKKQASVKVKPKAEEVIDIEAGPDKEVQKN	943.5485	4712.619	5
SAQILAKVQAAAKNNKKQASVKVKPKAEEVIDIEAGPDKEVQKN	943.5484	4712.619	5
SAQILAKVQAAAKNNKKQASVKVKPKAEEVIDIEAGPDKEVQKN	943.5472	4712.619	5
SAQILAKVQAAAKNNKKQASVKVKPKAEEVIDIEAGPDKEVQKN	943.5472	4712.619	5
SARLIKGMK	502.3724	1002.601	0
SASAKRSHESWISASSGGEELNFRQRSNSPGGQSVEPA	997.6902	3986.886	0
SASKKTK	375.2504	748.4443	0
SAVIKLLKKFIR	708.4626	1414.939	2
SAVKERPTLGGDTIGIIDIAFGS	773.0081	2316.232	3
SCLKKK	706.507	705.4207	0
SCNLDDDFFSIDFTWFA	511.4301	2041.841	0
SCPKSNCCKEGCGFG	760.198	1518.572	0
SCRKK	311.1849	620.3428	2
SCSPCVR	376.259	750.3153	0
SCYAVGLH	425.1352	848.3851	0
SDFLALNNAS	351.1757	1050.498	0
SDNKLVGS	819.449	818.4134	0
SDPPKSHDADDKLF	786.3582	1570.726	2
SEEGQALMAMKALF	763.3296	1524.732	2
SEKVTRTRI	545.371	1088.63	2
SENAGNKQHQNIASNGNS	935.4463	1868.836	2
SENKPTLLWKMPQNPS	935.4949	1868.945	0
SERRSRFAS	548.3516	1094.558	2
SESAYSDPRVNTESSEVNEPGDNSYKESNTPHI	910.6645	3638.588	0
SEVVDLVLSLDVSDDDVDGLLKRWIGRFA	808.5792	3230.682	0
SFGKSLFPM	507.3619	1012.505	2
SFNQHFDDIC	409.1613	1224.487	0
SFQDPGLIHGQTQAGANQTQTPSNDQSLF	772.5836	3086.433	0
SFQDPGLIHGQTQAGANQTQTPSNDQSLF	772.5839	3086.433	0
SFSNRASRLSSN	663.3461	1324.648	2
SFSSSSIC	409.1614	816.3324	0
SFTLKKTITL	576.3834	1150.696	2
SGDPLEDFCETNPADEEC	493.3848	1969.719	0
SGGVGLLLLIFSF	661.9582	1321.765	2
SGILIAGGTAAYVQSRFRVNKHDLFGHCNGHN	860.6646	3438.712	0
SGLDSTTA	376.2608	750.3395	2
SGMRKTRRK	560.4135	1118.646	0
SGNGNHQHNNKNKNK	423.361	1689.804	0
SGQCD	255.065	508.1588	2
SGQYTAKSAYGVLWGEMFEEQQDGVF	732.5475	2926.312	0

SGRHALRKRLEELGYELNDDQVQTLF	772.5821	3086.59	0
SGRKALQT	430.8365	859.4875	2
SGRNKNSS	425.2151	848.41	0
SGRYGGGRR	965.5078	964.4951	0
SHASSNY	383.1667	764.309	0
SHFLKLVHPGGF	669.9557	1337.725	2
SHGHNHTSHYPMH	771.3309	1540.638	2
SHKNEESC	467.2024	932.3658	0
SHKRWFL	487.323	972.5294	0
SHKTTSLFL	517.3697	1032.56	0
SHMPLIKQGPDH	680.4198	1358.677	2
SHNNFKAS	452.8032	903.4199	2
SIKILPVGIHIGQLQ	808.5786	1614.982	0
SIKRNFTNSM	599.3905	1196.597	0
SILLTSHGVDHLSSKF	581.0463	1739.921	3
SILYPKSELP	573.9063	1145.633	2
SIQALADDAELKQFRDPR	519.1139	2072.065	0
SIQKLLEKLKPN	705.9836	1409.861	2
SIQKQLV	408.2761	814.4913	2
SIQNQCT	397.1806	792.3436	0
SIVLTCVLELRW	716.4594	1430.796	2
SKAEGGPDKKRNAAADFLKRIKRNTSAEASKGGSGGSGGGGSGGGGGSSSSSKGGGG	857.219	5137.523	6
SKFKPVKN	474.3152	946.56	2
SKHVRSIVIGVGLMVCQ	609.4097	1825.007	3
SKKEEQDKLNCCEECASSYEKEAQLF	760.5816	3038.331	0
SKKRNKGGR	515.8631	1029.616	2
SKLAKEIAATVLLN	735.9954	1469.882	2
SKLGVSEKLAM	581.9034	1161.643	2
SKLIAGDSDFFAV	685.4317	1368.693	0
SKLKSLDLRRSKI	772.5015	1542.957	2
SKSISNGT	397.3027	792.3978	2
SKSTSKVKASSK	619.4356	1236.704	0
SKSWNVFAEILEKTHIITVPGSGFGPGGE	765.1537	3056.561	0
SKVRQVIN	472.3359	942.561	2
SLAALKLLKLLPGDVM	421.3226	1681.021	0
SLAMADEPVSRGDSPS	405.3501	1617.731	0
SLAPEQIDAEGETVSWTSGLIFGKGEGIVGQ	794.5634	3174.572	0
SLCDDLRVLGKQDF	537.0204	1607.798	3
SLGIIFLQILTAS	688.4383	1374.812	2
SLIVRAPITPIV	639.9449	1277.807	2
SLKTLGNKAMQSKK	767.4547	1532.871	0
SLLQIQLLVALR	683.9714	1365.871	2
SLLSDPASKP	507.8671	1013.539	2
SLLSDPASKP	507.8672	1013.539	2
SLNIPAQN	428.8003	855.445	2

SLNPHPPR	459.3455	916.4879	2
SLRTINSS	439.2769	876.4665	2
SLRTINSS	439.2769	876.4665	2
SLSFSSNCHVSSSAQATLQQQHEQQPLP	760.5832	3038.415	0
SLSMLEDIR	532.3572	1062.538	2
SMDLFCKKAF	595.376	1188.567	0
SMKSAYLADQRHR	781.8971	1561.778	2
SMRCVR	376.2591	750.3629	0
SNGYIFVGMETSAVE	535.3579	1602.724	0
SNIVTTTSS	455.2217	908.4451	0
SNIVTTTSS	455.3106	908.4451	0
SNKGKVSVAKKRK	477.2957	1428.889	0
SNKINNCG	425.2108	848.381	0
SNLTFNAMICGF	439.8803	1316.589	0
SNLVK	560.4137	559.3329	0
SNRLNSLNEM	393.2851	1176.556	0
SNSQNRAG	417.292	832.3787	2
SPCEPEESSLELTDNYPL	506.4773	2021.878	0
SPIIETRSRYPFQ	797.4004	1592.831	2
SPKGGSG	295.2259	588.2867	2
SPKPHIQTRFDVAVAIGFALLFFMLVFS	788.6061	3150.709	0
SPLFWIKLKNLF	761.483	1520.876	2
SPPPPSPPKAPKK	443.3026	1326.766	0
SPQKGNHL	441.2632	880.4403	2
SPRMKILQRIAYL	795.0384	1587.928	2
SPSHN	271.0602	540.2292	2
SPSIMKQDVAQDNTNSYSKNS	772.313	2314.038	3
SPSPQKPPPDVVRKERKK	519.1117	2072.186	0
SPSPQKPPPDVVRKERKK	519.111	2072.186	0
SQKIIYLLECNQQIHQQNAQLKEKVF	786.5976	3142.66	0
SQLLTILKHNIKL	507.6702	1519.945	3
SQVQPKYLI	538.3389	1074.607	2
SQYQDFLL	507.3536	1012.487	0
SRALLTLRGK	557.9144	1113.698	2
SRCFDVGCSSS	383.1672	1146.443	0
SRCFDVGCSSS	383.1653	1146.443	0
SRHKMC	381.1854	760.3472	0
SRKGLNKGKRVILP	783.5668	1564.989	2
SRPKLSFALGS	581.9046	1161.651	2
SRSRSRSRSGSPRHRRE	685.4126	2053.086	3
SSASHQPNGGDNNYNLNFSISTSTGLAGYN	772.6086	3086.36	0
SSCNCGSNCG	311.1457	930.263	0
SSDHVKAFDSAESWQSYWKEIKDSPK	764.549	3054.436	0
SSGEQYRLHVTTVSADSDMEMEDDITLSD	808.5802	3230.387	0
SSGSPQHTENAFGRVEWSDGKHLVGSPISVN	820.5942	3278.571	0

SSILQQQPWSIPPVPSFNPNP	778.3344	2332.185	3
SSIRARKIYVGLPAS	405.3499	1616.936	0
SSKGHKGISGF	552.8816	1103.572	2
SSKSSNLKFTGHH	477.3175	1428.711	0
SSLFNKSS	435.3061	868.4291	0
SSLKSSTLNAPGCSNATGACVYKASYGDTSF	772.5838	3086.396	0
SSNCDFTAQLAGFMAS	550.5015	1648.686	0
SSPDIYNPQAGSITTATSLDFP	766.9974	2298.054	3
SSPKPKLGQGC	551.3507	1100.565	0
SSRIALGNILKFRKKNWQ	720.5242	2158.249	0
SSRKRRKRR	615.4544	1228.77	2
SSRSRASDITENNNTS	435.3584	1737.788	0
SSSALAELDSQEDALYALKVSLNASPNQLTNWNKN	948.716	3790.865	0
SSSFSVSCSLMENQETHN	497.415	1985.809	0
SSSTNGT	327.2006	652.2664	2
SSVERAFSAMKIIKSKLRN	722.5022	2164.215	0
SSVNLKK	388.2538	774.46	0
SSVNLKK	388.253	774.46	0
SSVRV	274.2739	546.3126	2
SSVRV	274.2738	546.3126	2
STFVKSGSEAI	563.37	1124.571	2
STLKSTVNSP	517.3678	1032.545	0
STQIRNCSK	518.8427	1035.513	2
STRQYIK	895.5025	894.4923	0
STVANILSVAS	531.384	1060.576	0
SVKGSKTEWMSMSRNWGQNWQ	632.4692	2526.153	0
SVKGSKTEWMSMSRNWGQNWQ	632.4711	2526.153	0
SVKSKQPASK	530.4028	1058.608	0
SVLIFSIILN	559.8898	1117.675	2
SVSGKTGNKNNK	617.4026	1232.647	0
SVVYNNTT	449.2876	896.424	0
SWKPVGVSRSS	595.3807	1188.625	0
SWRVVVN	430.2895	858.4712	2
SYDGQFLASGC	383.1654	1146.465	0
SYKNAFKK	493.3102	984.5392	0
SYKPLKKKTYRDEKDGSGRFQRIEDGDHKKLIEA	1009.751	4035.129	0
SYNDFCNA	467.0997	932.3334	0
SYNENVFKGLDFIISEAGKNGIRLILSLVNNWN	935.497	3737.942	0
SYNKLQVSHNK	439.8961	1316.684	3
SYNLFTC	424.2713	846.3582	2
SYNVNQNVSQN	423.1957	1266.548	0
SYNYVCV	424.2705	846.3582	2
SYPDPNSGYGQGGYTYEQQQ	560.4126	2237.914	0
SYQNWRWQPHHCDLT	493.3835	1969.864	0
SYSRDRNYS	383.1654	1146.505	0

SYVSSTQCASAIRRRVMA	497.2326	1984.994	0
TACIPNSKDSSSHKDDETSLQRELRNAECMAS	881.6138	3522.577	0
TAHHR	311.1849	620.3143	2
TARAAPYAKARTQFSRDIQMTRHNSNNYGN	860.6659	3438.671	0
TARTMGSNGDANMKYNLTW	533.4523	2129.962	0
TDHDLILLSH	582.3703	1162.598	2
TFKPRFFQKKPL	513.0035	1535.898	3
TFRK	551.3523	550.3228	0
TFSSSLHNRNPLF	760.4921	1518.758	2
TFVLYLGPHIALFTL	569.0659	1703.965	3
TGPPKWIKVNERRTLHDVLK	796.5398	2386.36	0
TGREIVFQGFNWESWRRRWYL	697.4454	2785.399	0
TGRYEAHLWDNSCRREGQT	760.319	2278.03	3
THARWFN	466.3185	930.446	2
THILRSLASQN	620.4099	1238.673	2
THMRAKSG	444.3045	886.4443	2
THNNQTDIVFVRNSGFCQDGTLI	645.4875	2578.223	0
THNSILCCDFNYVNLSNQRGT	600.4679	2398.079	0
THNSSNHH	467.2049	932.3849	0
THQNSF	367.1709	732.3191	0
THQNSF	367.1715	732.3191	0
THQNSF	367.1711	732.3191	0
THQPLASPHNPNDKAHASSSTSTRHMAQWE	831.6277	3322.529	0
THQQILEQQMNC	369.1832	1472.639	4
TIGNTLSSVPFLLLMSLIPG	519.1119	2072.159	0
TIMVKLGREV	573.4029	1144.664	2
TIPLNSTC	425.2087	848.395	0
TIPLNSTC	425.2111	848.395	0
TIPRALHLGVN	595.9194	1189.693	2
TIYARYI	450.2958	898.4913	2
TIYKAVSEV	505.3503	1008.549	2
TKGFYLLGLEKSSPTFAAAM	711.4603	2131.102	0
TKGRRSMK	963.5951	962.5444	0
TKHVVGTYGYLPPEYIIDGAYSTKSDVFSFGVL	910.6619	3638.818	0
TKKPKRVDS	529.8798	1057.624	2
TKPFNINN	474.3148	946.4872	2
TKVLKPSSKKAF	667.436	1332.813	0
TLALCYN	399.3061	796.3789	0
TLGEASLRVKRN	672.4336	1342.768	2
TLIKGLGSH	463.2991	924.5392	2
TLIKGLGSH	463.2986	924.5392	2
TLIMLSVIRN	588.3919	1174.674	2
TLKAKPTHDRKK	712.0105	1421.847	2
TLKNQSTDPSIYSEAQYVGSS	569.4708	2274.065	0
TLLEKSQAKLVN	672.4336	1342.782	2

TLLLALAGKLGK	599.3936	1196.786	0
TLLTSKASAIAKALSAPLQ	628.7575	1883.109	3
TLNKKKN	423.3555	844.5131	2
TLNKKKN	423.3623	844.5131	0
TLPQDSTCTLPCSLTETDPSTF	786.3353	2356.045	3
TLRRIPSSPSLLLGIKK	627.1062	1878.178	3
TLTGDPADNRRNVFNILSGTSM	595.4483	2378.165	0
TLTLVFKRN	546.3964	1090.65	2
TLTLVFKRN	546.3964	1090.65	2
TLVSLLYLIKPLF	760.4877	1518.943	2
TNDIEILHTDTLIKEVEKVF	786.3332	2356.253	3
TNLFYYLIVLVSV	772.5015	1542.87	2
TNVLLKTKS	502.3716	1002.607	0
TPAPAPAPAPLLPP	663.3461	1324.739	2
TPAPAPAPAPLLPP	663.3461	1324.739	2
TPFYCSQFQLPSNSNN	462.4487	1845.799	0
TPKSLIGNLTN	579.4249	1156.645	2
TPLIPVPPKSLIF	711.504	1420.869	2
TPLLRERIFH	641.4213	1280.735	2
TPLVGIYSIIH	606.8972	1211.691	2
TPNKAFAQTVIK	659.4802	1316.745	2
TPPPVIGKI	461.3621	920.5695	2
TPQWRAGPL	513.3854	1024.545	2
TPSSNNAPVSGFGS	441.2802	1320.595	3
TPTMGTTPTTGTPSTSTGTGTGTGTTTGTTPYSTTPGVLGGIG	983.7122	3930.853	0
TPYFPLPKPY	611.9134	1221.643	2
TQKILLRSPPVTRRR	607.7579	1820.122	3
TRTKGVRRN	544.413	1086.637	2
TSKGKIPLSKAPIPRNNGK	502.3714	2005.18	0
TSRKGYLAAISA	413.2647	1236.683	0
TSVGKDRPRQKN	693.3832	1384.754	2
TSYTCLISAYGKQK	781.8971	1561.781	2
TTANGRVG	388.2536	774.3984	0
TTFIQKST	463.3267	924.4916	0
TTFIQKST	463.3273	924.4916	0
TTHVMYINFYLLDYGWNMIVCVVMAM	786.599	3142.45	0
TTPVTT	619.4379	618.3224	0
TVLKSKKKKS	573.9063	1145.75	2
TVLKSKKKKS	573.9063	1145.75	2
TVPVVSSTTT	496.3276	990.5233	2
TWHANGRNCDGMGHHAC	467.4047	1865.726	0
TWIKLEKKIS	623.4512	1244.749	2
TYQKGKKSKNP	639.9449	1277.709	2
TYSIVMEVLGYCGYLEEAEAVFFEMKQN	816.6381	3262.491	0
TYWNLKGQNSG	423.197	1266.599	0

VADLISVVEVAASD	694.4468	1386.724	2
VAIPSPISPVLNDKPPKN	472.3601	1885.067	0
VAIYTDIV	447.3069	892.4906	0
VAKASSTASAAAAAAAADRKQKASVFSRISF	760.5821	3038.626	0
VALIFYGIIARVVAFFCLV	705.51	2113.216	0
VALPCRVCRGKGF	469.3265	1404.748	0
VALPQEC	380.2466	758.3633	2
VANRIVLP	441.2967	880.5494	0
VAPIRLPVDLALSL	492.9949	1475.908	3
VAPLLIPHQ	494.3191	986.5913	2
VASSGIVSLLL	529.8798	1057.638	2
VAVKRIHSVLVL	667.4361	1332.861	0
VCLVMTIGN	475.3274	948.4772	2
VCQGGNLRKKFYRKSCPQAEQIVRTK	760.1988	3036.623	0
VCWRTL	389.2645	776.4003	0
VDDHCGLLLPGNLFHIFFKNNSAYHDVHHQLY	941.7025	3762.815	0
VDEGQCD	383.1659	764.2647	0
VDHLIVRGG	483.3032	964.5454	2
VDRFLNSGILIEILSH	609.3945	1825.01	0
VDYVLVVF	477.3173	952.527	0
VENMCHFDADGKRYYPFGRGSGSQVVQQ	794.5618	3174.44	0
VEVIPIPN	440.8135	879.5066	2
VFALNSMLPHTHAGTSSRELF	772.3231	2314.153	3
VFEYRIPKQ	590.4227	1178.645	0
VFLWNKKHNAKEAKQVQN	546.3996	2181.181	0
VFQRERLN	531.3856	1060.578	0
VFRRGGALCVVG	617.418	1232.681	2
VGFYECGRVERHHVLVGKT	547.3971	2185.122	0
VGGGVVGPLLAAGP	582.3694	1162.671	2
VGGTIKHISWVHN	483.3206	1446.773	3
VGLLCGCT	383.1647	764.3561	0
VGLLGFGC	383.1656	764.3891	0
VGPVAVGVKSG	485.353	968.5655	2
VGRGIMGYL	483.3032	964.5164	2
VGVPMAFTFEIYGDGTASSRDCFKMFNP	772.5826	3086.398	0
VHALVTASCRGLVDVVETLIKCGVDAS	689.5168	2754.441	0
VHGFYKM	441.2617	880.4266	2
VHGFYKM	441.3157	880.4266	2
VHGFYKM	441.2068	880.4266	0
VHGFYKM	441.2963	880.4266	0
VHIRKRVKASK	441.2802	1320.847	3
VHKKLQK	440.8135	879.5654	2
VHLIIPKKQ	538.3779	1074.691	2
VHNNAF	351.174	700.3293	0
VHNNAF	351.175	700.3293	0

VHNNAF	351.1764	700.3293	0
VHNNAF	351.1763	700.3293	0
VHNNAF	351.1754	700.3293	0
VHNNGVFVTLPATSQIS	595.3783	1782.926	0
VHNQLGSSQLH	610.3956	1218.611	2
VHNTPCNVNVGFG	453.2064	1356.625	0
VHNTTVFS	452.8041	903.445	2
VHPGSVTGTGTGTVSFMQERNNNAGYSLAVPTTGNEI	941.7019	3762.791	0
VHRIIKMKKLYGGVLI	623.4103	1867.159	0
VHRPFIRKA	562.356	1122.678	2
VHSFR	323.1452	644.3394	2
VHSSIFSLKKLEK	758.4735	1514.882	2
VHSVFATS	424.2705	846.4236	2
VHVDCHSTMSNETGDICDLGQFRRLILSPLYVKE	969.7344	3874.88	0
VIAIQGKISFGSF	456.288	1365.766	3
VIGIQYLQYAHKK	780.9461	1559.882	2
VILEFH	757.469	756.4171	0
VILLRLPLRN	603.9162	1205.797	2
VILLRLPLRN	603.907	1205.797	2
VILPIQMQKK	599.3929	1196.731	0
VIPLKLPGLKHIILVGDEKQ	737.4807	2209.356	0
VIRLHQNNFS	614.3796	1226.652	2
VIRTSVGGRNG	558.4031	1114.621	2
VITVRHEKKVVF	727.9979	1453.877	2
VIVAHRLNTIRN	703.5068	1404.831	2
VKDFLILNLSTV	681.4929	1360.797	2
VKEKKKPN	485.854	969.5971	2
VKEKTTSH	465.3193	928.4978	2
VKELKPVK	470.8311	939.6117	2
VKKSRKGKIN	579.425	1156.741	2
VKLGTQGLVLLLI	683.9713	1365.896	2
VKPLSLMRSVAVGTG	758.0119	1513.865	2
VKPLSNAFVINVGDM	535.3689	1602.844	3
VKSNSKSKITI	602.9103	1203.719	2
VKSVRPRG	449.8241	897.5508	2
VKTLEDEKIALL	686.4493	1370.802	2
VKVEVTHGKN	555.8443	1109.619	2
VKVGTPIILLRN	661.9583	1321.845	2
VLDDATHHHHHEC	388.3928	1549.648	0
VLDIILVPIASEKQ	513.3752	1536.913	0
VLEEQKWMRYIKLN	925.5143	1848.992	0
VLGAAIVVIGF	529.8806	1057.654	2
VLGSVLLGASV	507.8671	1013.612	2
VLGVDFASVHIYPDSWISQSVADSHLPFI	800.6123	3198.603	0
VLGVKNANSRVGR	685.4283	1368.795	2

VLIEKGKLGKKRVS	778.055	1553.998	2
VLIEPKNALAKQ	662.4816	1322.792	0
VLIPFLKKKM	406.3263	1215.778	0
VLKLAAQN	428.8009	855.5178	2
VLKTLEMDEEY	685.4335	1368.648	0
VLKWLSLAQHPNVV	535.3689	1602.925	3
VLLVVLLAIVGM	620.4098	1238.804	2
VLNHPSIGVFLTHCGWNSTTES	600.4679	2398.138	0
VLQNYKVNFIPG	696.4341	1390.761	2
VLRKKPDFRRK	721.9681	1441.899	2
VLVDNRERDSGSM	493.3084	1476.699	0
VLVGNKVVGEIAGPNK	797.4634	1592.925	0
VLVGNKVVGEIAGPNK	797.4685	1592.925	0
VLVLPFPFQ	538.3403	1074.611	2
VNGTCCEKYCGCSKHCSNRFRGCR	678.4756	2710.123	0
VNHKAEKKKYVLKKI	609.3943	1825.13	0
VNIDSQCSADKFPVMDGKVQSETPCQEIPSIAS	881.6132	3522.632	0
VNMKGLDGIQGPMYVGTGCVFNRQALYGYSPPSMPK	969.7307	3874.867	0
VNPNNYE	425.2139	848.3665	0
VNPNNYE	425.2085	848.3665	0
VNPRWLVIHKLKLALFVDKI	801.4947	2401.473	0
VNPVKYLNLR	608.3841	1214.714	0
VNQLLEQKELTFDVSDYLWYMTSVDIN	816.6375	3262.575	0
VNSHFLERLY	639.4431	1276.656	2
VNVALTCHH	497.2327	992.4862	0
VNYGMHGVTRSCGECAICLEEFEVGQ	708.4871	2830.218	0
VPAVCGFSGLPLFPSQSQRNRDSNNTP	963.489	2887.404	0
VPCIPAII	413.2651	824.483	0
VPCIPAII	413.2628	824.483	0
VPDNSAKALSRYTEMVDDVI	556.4369	2222.089	0
VPGGLAAALL	441.2971	880.5382	0
VPGVDLKRR	520.3324	1038.63	0
VPGVDLKRR	520.3334	1038.63	0
VPHLNWQ	447.3078	892.4556	0
VPKGGVADAQ	941.5076	940.4978	0
VPKKVVAHLLKPRGWKPP	513.4105	2049.273	0
VPKRLNPQTS	570.3664	1138.646	2
VPNAAPPES	441.2065	880.4291	0
VPNAAYAGGPPGG	564.3597	1126.541	0
VPNALIPGT	441.2961	880.5018	0
VPNFFKTSHASS	441.2967	1320.646	0
VPNFFKTSHASS	441.2068	1320.646	0
VPNGGVEIHLVKNRET	441.2969	1760.953	0
VPNIYIY	441.3091	880.4695	2
VPNIYIY	441.3157	880.4695	2

VPNIYIY	441.2967	880.4695	0
VPNIYIY	441.2999	880.4695	2
VPNKLVVL	441.3157	880.5746	2
VPNKLVVL	441.297	880.5746	0
VPNKLVVL	441.3132	880.5746	2
VPNKLVVL	441.2958	880.5746	0
VPNKLVVLN	498.348	994.6175	2
VPNLAMKLEALRDEY	441.2952	1760.913	0
VPNMEVQSCSID	441.2962	1320.569	0
VPNMEVQSCSID	441.2072	1320.569	0
VPNMEVQSCSID	441.2045	1320.569	0
VPNMTHIA	441.7656	881.4429	2
VPNMTHIA	441.8273	881.4429	2
VPNMTHIA	441.8273	881.4429	2
VPNMTHIA	441.7656	881.4429	2
VPNMTHIA	441.7656	881.4429	2
VPNNQFPGAWGS	425.2133	1272.589	0
VPNNQFPGAWGS	425.2127	1272.589	0
VPNNYDMI	499.2258	996.4222	2
VPNPDGIIG	441.2977	880.4655	0
VPNYLFE	441.3132	880.433	2
VPPKPLLIHVASSSAFSFACNFGHDHDKDKGSLSSSDNES	1057.718	4226.997	0
VPPSSTSS	381.1864	760.3603	0
VPPSSTSS	381.1861	760.3603	0
VPPSSTSS	381.1854	760.3603	0
VPPSSTSS	381.1852	760.3603	0
VPPSSTSS	381.1863	760.3603	0
VPPSSTSS	381.1865	760.3603	0
VPQNSTPCA	458.812	915.412	2
VPRKIIR	441.3091	880.597	2
VPRKIIR	441.3113	880.597	2
VPRKRNI	441.8273	881.5559	2
VPRKRNI	441.8273	881.5559	2
VPRKRNI	441.8273	881.5559	2
VPRKSVSFAKIAVP	500.3448	1497.903	3
VPRLREIIN	555.3414	1108.672	2
VPRNDVFS	467.3159	932.4716	0
VPRRIKL	441.2971	880.597	0
VPVPSGGFNFS	554.3718	1106.54	2
VPWNDLGFSVNNTVWLSC	513.4105	2049.962	0
VPWYNS	383.1659	764.3494	0
VQIYKAPWSIRDK	802.5187	1602.888	2
VQLASLIGLLIRH	478.3202	1431.893	3
VQLNLLLP	455.3106	908.5695	0
VREQGGRVLVH	625.4224	1248.705	2

VREVFIAKRRNKTG	419.3139	1672.985	0
VRLLLF	760.5838	759.5007	0
VRPKRVLRQ	576.4109	1150.741	2
VRPPSVLAEKNNAEKTTRRSRTREISS	771.4849	3081.676	0
VRVRIQNNFLSGT	752.4873	1502.832	2
VSASLYK	767.4549	766.4225	0
VSETVAGVVTIRAF	483.6518	1447.804	3
VSIGPE	301.1388	600.3119	2
VSKKR	617.4037	616.402	0
VSMVPT	317.2078	632.3204	2
VSPLNLPSLPIP	623.9572	1245.733	2
VSQGNKWCDFCKIY	423.3622	1689.764	0
VSQVVSENNSLTS	455.3112	1362.663	0
VSYLNRSS	463.2998	924.4665	0
VTEALVNKCQLVFLPRLGADHIINARMFSRKLKVGV	1009.75	4035.275	0
VTGLQLIFSARVPIIKVT	652.4083	1954.198	0
VTILPTLVINNR	676.9495	1351.819	2
VTLRFLCRLN	617.9318	1233.702	2
VTNRKKAP	913.5134	912.5505	0
VTNRKKAP	913.5141	912.5505	0
VTPVVVTT	815.5121	814.48	0
VTSRVAFPKFGTPYVLKPLAHE	615.1364	2456.358	0
VVEGKLRPK	513.3869	1024.639	2
VVEIGPLIVN	526.886	1051.628	2
VVELSVALHH	552.3605	1102.614	2
VVELSVALHH	552.3605	1102.614	2
VVGEAGPLKR	513.3869	1024.603	2
VVHPRNGS	433.2911	864.4566	0
VVIPSAGFSH	507.3645	1012.534	2
VVKKCVEGVIKQSG	737.5008	1472.839	0
VVKKIRPHVLLGLSG	808.5806	1615.03	0
VVKNGT	617.4035	616.3544	0
VVKPPVTIPPIN	637.4669	1272.781	2
VVKVNSNPQENLTKKQ	913.5167	1825.006	0
VVKYLAELPQN	637.4669	1272.708	2
VVLAGH	595.3798	594.3489	0
VVLDELLKLLPLKSRVFVV	727.4583	2179.371	0
VVLLLVWFS	538.3447	1074.648	2
VVLLLVWFS	538.3447	1074.648	2
VVLMK	295.2259	588.3669	2
VVLMK	295.226	588.3669	2
VVNKYKGLGAKSVT	732.4769	1462.851	2
VVNNGPRPAHT	581.3817	1160.605	0
VVNVTTGENEDR	667.2889	1332.616	2
VVQLAQRNSS	551.3518	1100.594	0

VVQQQQMP	957.5045	956.475	0
VVRSS	274.2739	546.3126	2
VVSKKLRFKKT	667.4776	1332.861	2
VVSLGAIGLLKSFRV	780.026	1557.961	2
VVYAGLIM	433.2911	864.4779	0
VVYPVYRN	505.3889	1008.539	2
VVYPVYRN	505.3889	1008.539	2
VVYVILLC	461.3176	920.5405	2
VWIDKIVPRWIF	786.4819	1570.903	2
VWPNHL	383.1649	764.397	0
VYIIYSSH	491.3333	980.4967	0
VYIKSCDKLDKIF	786.4763	1570.843	2
VYIKSCDKLDKIF	786.507	1570.843	2
VYIKSCDKLDKIF	786.507	1570.843	2
VYIKSCDKLDKIF	786.4774	1570.843	2
VYQNNNSSSS	367.1699	1098.458	0
VYQNNNSSSS	367.1714	1098.458	0
VYRNRISPDPIFLTTEAPSVGGFYAINRRGQVLL	955.754	3819.058	0
WAECHQ	387.1737	772.2963	2
WAEHTGTIEACFKE	811.4167	1620.724	2
WALNCVATTDQGENDPSSLWLNPEIKDS	776.5708	3102.424	0
WERILVKYG	582.4087	1162.65	2
WGSGGRGGHGDSGYGGRNNDSGYGGRGNDAGYGG	808.5797	3230.328	0
WIGEP	301.1421	600.2908	2
WIVKFLLQR	601.9441	1201.734	2
WKAKNFS	440.7735	879.4603	2
WKERRL	444.3045	886.5137	2
WKERRL	444.3045	886.5137	2
WKGCFGKKSSLE	685.4283	1368.686	2
WKLVS	632.4721	631.3693	0
WKRMAVSWTG	611.3525	1220.612	0
WKWSLTG	439.2888	876.4494	2
WLLPILA	413.2657	824.516	0
WMFRDQRVTDN	734.3191	1466.673	2
WNKPANK	429.3174	856.4556	0
WNKPANK	429.3156	856.4556	0
WNSTLKKRLKANTSTPS	966.5973	1931.059	2
WRLPALGPSHVLQ	737.5011	1472.825	0
WRWQPKGCDLPRFNATKMLELIRGKR	800.6112	3198.717	0
WSDRGSLWADDCSLSFATN	533.4507	2129.911	0
WSIRRKIAIGAAR	375.3226	1496.905	0
WTGTDCCHNWYGISCDRN	533.4506	2129.814	0
WTHTDCAIREQLICMGPSAKNGAGPSEIVF	808.58	3230.531	0
WVGIALFFLAF	642.4225	1282.711	2
WVHQYL	423.1981	844.4232	0

WVPCTGC	383.1667	764.2986	0
WVPCTGC	383.166	764.2986	0
WVPCTGC	383.166	764.2986	0
WVPCTGC	383.1652	764.2986	0
WVTGLLIKAI	557.412	1112.696	2
WYNHCLLRYNDTPSYSTLNTSSPS	705.5093	2818.266	0
YAECGDIENSRKVFDGVCLGERCEALWNTLL	876.6549	3502.632	0
YAEQQLNAY	367.1722	1098.498	0
YAEQQLNAY	367.1705	1098.498	0
YAKGIGL	721.5011	720.4171	0
YAQELMSLNLEEGSSGQD	493.3846	1969.858	0
YAQKTLPPRKK	665.4701	1328.793	2
YCNNEYGNITTET	381.1883	1520.609	0
YCPATKCPALSEDDNLYDDF	760.5816	2278.94	0
YCVWENNLHQGHNHWN	513.4108	2049.865	0
YDFGMMGE	483.1742	964.3306	2
YDHIIPYSKGGES	367.1717	1464.689	0
YDNLIFHRVIKGFMI	467.3148	1865.002	0
YEKMLENLKKLKD	551.3677	1650.902	3
YFGVDNEVGSLDWASIV	468.4403	1869.879	0
YFKCSYA	441.2058	880.3789	0
YFKCSYA	441.2064	880.3789	0
YFNLQNAPEQNLSPADDDGHGT	601.4633	2402.041	0
YFNYKD	425.2114	848.3705	0
YFNYKD	425.2112	848.3705	0
YFSVKFG	424.2721	846.4276	2
YFVVCGIGPEIRTMDGNKGY	555.4565	2218.055	0
YGGDISRKKK	576.409	1150.646	0
YGGRRDQDPGNQDWNNHRN	766.9986	2298.002	3
YGKNELFDSEYLW	555.2745	1662.757	0
YGNKPVTIKGIK	659.4802	1316.782	2
YGNYGDAYASYGDYGQYGN	525.4464	2097.786	0
YGQALPSYSNSSYGAGYTQTPAYT	637.4777	2546.124	0
YGQLSISLPVARNCL	409.2173	1632.866	0
YGRGSIVRGG	511.3148	1020.546	2
YGRILSQCLVNT	683.9714	1365.708	2
YGRKQQQK	518.3412	1034.562	2
YGRQRKALKM	625.9286	1249.708	2
YGRQRKALKM	625.9286	1249.708	2
YGRQRKALKM	625.9298	1249.708	2
YGTPVLQPFPPPN	476.3064	1425.729	0
YGWVA	595.3766	594.2802	0
YICSRYYRAPELIFGATEYTASIDIWSAGCVLAELLLGQPLF	786.3422	4712.37	6
YIEWGLKEPIKKVTEI	649.4456	1945.092	0
YIGLVADTLRN	617.9318	1233.672	2

YIHLVQKK	514.859	1027.618	2
YIKLHH	405.7843	809.4548	2
YIRNKNIRKLREEEGHFCQKIR	708.4878	2829.53	0
YKDPQERERRF	762.3227	1522.764	2
YKIEEKKK	533.3463	1064.623	2
YKINITRGSK	590.4237	1178.677	0
YLLISKKRYAGLF	786.5389	1570.924	2
YLMKLGL	837.5853	836.483	0
YLQWQQW	351.1758	1050.492	0
YLTRKN	794.5619	793.4446	0
YLYEWKQSLDDPDSSLSSWNNR	676.4982	2702.225	0
YMKNASQ	421.292	840.38	2
YMSFYNVFFTSLPVIALGVF	772.3166	2314.175	3
YMWGNANDS	353.156	1056.397	0
YNCERVISLDKHDGI	441.2957	1760.852	0
YNILSTVKLTFAASLMSNSC	541.4406	2162.075	0
YNRLGRDMAYYPPAENLNEEPLDEAV	760.582	3038.408	0
YNVPSKKAFN	584.3801	1166.608	2
YNYGLNRTSSF	441.2074	1320.61	0
YRECDIYGTVDFIFGNAAAVFQNCNI	736.5298	2942.337	0
YRNSRGSQGNDRNRKSFEDFDGPSRQSY	831.6263	3322.521	0
YRRGAKPRKQGF	732.4827	1462.827	2
YRSQKYHSEK	663.3461	1324.652	2
YRVGSKK	419.2505	836.4868	2
YRVGSKK	419.2998	836.4868	2
YSATCHILGHYG	441.2061	1320.592	0
YSGLLISRALSFSNFS	441.2955	1760.91	0
YSKECQ	379.1707	756.3112	0
YSQSSKSS	437.2106	872.3876	0
YSRAHKPQLVLD	476.3238	1425.773	3
YTEKTGFKTCNAC	367.171	1464.638	0
YTKLIQIH	508.3337	1014.586	2
YTLPYNLLDWNQAKNSC	511.4311	2041.957	0
YTLVASKKMV	570.3698	1138.642	2
YTVDEGSRLQRLFWCDTESQLLYGVFGDIL	881.6153	3522.713	0
YVDAIGVY	450.2942	898.4437	2
YVKMQYCVSCDIHSMLGMMHKGPVSLVS	786.5985	3142.46	0
YWFFFSPVDFKYSRSKRFNRTT	720.524	2878.434	0
YYKQENARKGL	457.2701	1368.715	0
YYPACPEPELTMGTSRHTDGNFMTI	708.5061	2830.24	0

Prec m/z: Precursor mass-to-charge ratio, Theor MW: theoretical molecular weight, Prec z: Precursor charge

[d]Chemically synthesized peptides are labelled in bold
